# The Labrador Sea as a conduit for AMOC lower-limb transport anomalies on multiple timescales

**DOI:** 10.1038/s41467-026-77271-z

**Published:** 2026-08-28

**Authors:** Jia-Jia Chen, M. Susan Lozier

**Affiliations:** School of Earth and Atmospheric Sciences, Georgia Institute of Technology, Atlanta, GA USA

**Keywords:** Physical oceanography, Climate and Earth system modelling

## Abstract

The Atlantic Meridional Overturning Circulation (AMOC) is an essential element of the global climate system, yet its variability on timescales longer than those observed remains unsettled. Recent studies show that transport variability in the lower AMOC limb is maximized on density surfaces well below the density of maximum overturning. To explore the source and impact of this feature, we analyze lower-limb transport variability using climate simulations and observations from the Overturning in the Subpolar North Atlantic Program. We find that enhanced variability at select densities reflects transport redistribution within the lower limb and is not strongly linked with local or downstream AMOC variability on monthly to multidecadal timescales. Furthermore, the strong transport variability in dense waters leaving the Labrador Sea is tightly linked to variability entering the basin, with the opposing inflow and outflow largely canceling in basin-integrated diagnostics. These results underscore the need to account for density-space compensation and cancellation of opposing flows when interpreting the climatic relevance of strong subpolar transport variability.

## Abstract (web summary)

The study shows that, despite the Labrador Sea forming the final leg of the Atlantic overturning circulation’s subpolar ‘relay’, transport changes originating in the Irminger and Iceland basins exert the strongest downstream influence.

## Introduction

The Atlantic Meridional Overturning Circulation (AMOC) plays a central role in the Earth’s climate system by redistributing vast amounts of heat, salt, and carbon globally. Its upper limb carries warm waters northward from the tropics to polar latitudes, releasing heat to the atmosphere along the way, thereby impacting the weather and climate of surrounding regions^1,2^. As these waters lose heat, they become denser and sink, forming North Atlantic Deep Water (NADW), which includes upper NADW formed in the subpolar North Atlantic and lower NADW comprised mainly of denser overflow waters originating from the Nordic Seas. These waters constitute the AMOC lower limb that flows equatorward throughout the Atlantic.

With AMOC changes historically linked to deep water formation on various timescales^2,3^, oceanographers have long been interested in the amount of deep water formed in the Labrador, Irminger and Nordic Seas^4–8^ to understand how variability in each region impacts the AMOC. Over the past two decades, modelling studies have indicated strong links between Labrador Sea deep convection, density and AMOC variability^7–10^. However, summer hydrographic surveys from the 1990s^11^ suggested that the Labrador Sea contributes minimally to NADW volume. Consistent with these earlier estimates, direct observations from the Overturning in the Subpolar North Atlantic Program (OSNAP)^12^ later revealed that overturning across the eastern OSNAP section (i.e., in the Iceland and Irminger basins) dominates both the mean and monthly variability of subpolar overturning, with minimal contribution from the Labrador Sea^13^.

One modelling study^14^ attempted to reconcile the discrepancy between OSNAP observations and earlier modelling studies linking AMOC variability to Labrador Sea processes. It suggested that subsurface density anomalies in the Labrador Sea are strongly influenced by signals originating in the Irminger Sea, implying that the Labrador Sea is unlikely to be the primary source of AMOC variability. Another eddy-resolving modelling study^15^ showed that low-frequency AMOC anomalies at 45°N are preceded by pronounced lower-limb transport anomalies associated with enhanced water mass transformation (WMT) within the Labrador Sea Water (LSW) density range. On this basis, the authors argued that although the Labrador Sea contributes only weakly to the mean and monthly variability of the AMOC, it may play a disproportionately important role in driving low-frequency variability. Recent observational analyses, however, complicate this interpretation. While surface-forced water mass transformation accounts for a substantial fraction of the mean lower-limb transport, its variability explains only part of the AMOC variability on seasonal to interannual timescales, with the remainder arising from basin storage, interior mixing and advective processes^16^. Together, these studies leave unresolved how transport variability within specific density classes, including the LSW density range, relates to AMOC variability across timescales, and which density-space transport anomalies more effectively capture AMOC variability, both at the OSNAP section and farther downstream.

Here, we show that pronounced lower-limb transport variability largely reflects redistribution among density classes and, on monthly to multidecadal timescales, is only weakly related to AMOC variability at OSNAP and farther downstream. In the Labrador Sea, both the dense-water inflow and outflow correlate with AMOC variability, yet their opposing contributions largely cancel upon basin integration, thus substantially weakening the basin-scale relationship with the AMOC.

## Results

**Fig. 1:**
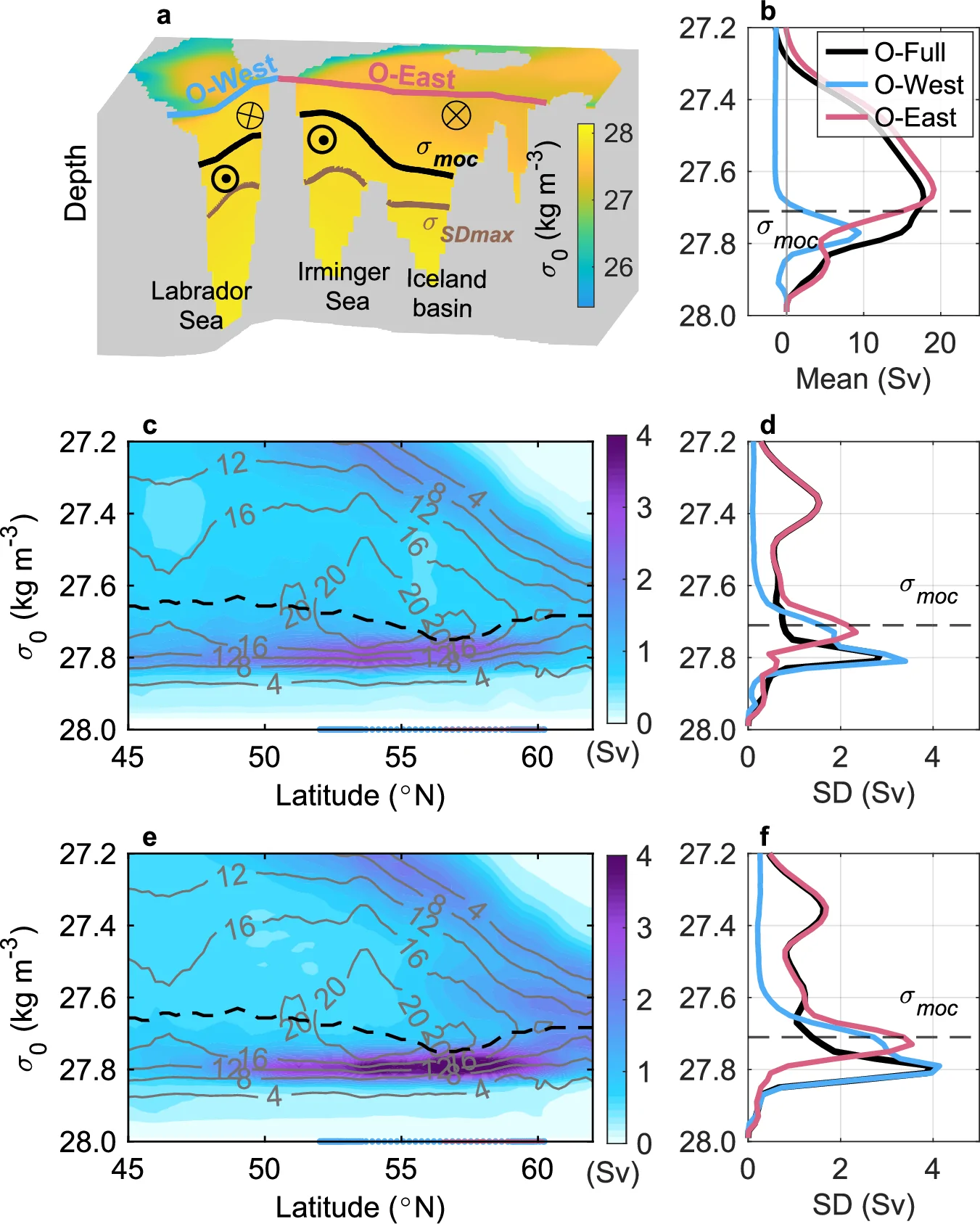
Mean and variability of the overturning streamfunction in density space using CESM2. **a** Schematic diagram of O-West and O-East sections and frequently used isopycnals in this study (${\sigma }_{{moc}}$ and ${\sigma }_{{SD}\max }$). ${\sigma }_{{moc}}$ (black line) is the density of the maximum streamfunction for O-Full. ${\sigma }_{{SD}\max }$ (brown line) is the isopycnal of the maximum standard deviation (SD) of the streamfunction for O-Full. Circles with crosses and dots represent northward and southward flows, respectively, while shading indicates CESM2 time-mean potential density. **b** Time**-**mean overturning streamfunction across the three sections indicated. **c** Latitude-density cross-section of the time-mean overturning streamfunction (gray contours) and low-frequency (LF; variability on timescales > 20 years) SD (colored shading). The black dashed line denotes values of ${\sigma }_{{moc}}$ for each latitude. Blue and red dots alone the x-axis indicate the latitudinal positions of the O-West and O-East arrays, respectively. For reference, the OSNAP array spans approximately 52-60°N. **d** LF SD of the streamfunction across the three se**c**tions indicated. **e,** **f** Same as **c,** **d**, but for high-frequency (HF; variability on timescale <10 years) SD. All quantities are computed from the 300-year CESM2 simulation.

To examine overturning variability, we analyze pre-industrial control (piControl) simulations from nine coupled climate models participating in the sixth phase of the Coupled Model Intercomparison Project (CMIP6) (^17^; Supplementary Table 1) and one high-resolution climate model used in Yeager et al. (2021)^15^. These models provide the required diagnostics and span a range of model formulations and horizontal resolutions. The spatial configuration of our study is mainly based on the geography of the OSNAP observing system, which spans the subpolar North Atlantic across a full section (O-Full) composed of two coast-to-coast subsections, O-West and O-East (Fig. 1a).

Focusing on internal variability, we analyze the first 300 years of each piControl simulation to ensure consistency across models with different run lengths. Unless otherwise indicated, all statistics reported in this study represent multimodel means. Results from two models CESM2^18^ and MPI-ESM1-2-HR^19^ are used as representative examples in selected figures. OSNAP observations^20^ are used to assess whether the structural feature of overturning variability identified in the models are also evident in observations on monthly timescales. While direct model-observation comparisons are limited by differences in external forcings and record length, the observations provide important context for interpreting simulated variability.

### Density structure of transport variability across timescales

We begin by characterizing the overturning streamfunction as a function of density across the OSNAP sections, using CESM2 as an illustrative case (Fig. 1). Here and throughout the manuscript, MOC strength refers to the maximum of the density-space overturning streamfunction, with the corresponding density denoted as ${\sigma }_{{moc}}$. As such, the standard deviation shown in Fig. 1 represents variability of the overturning streamfunction at each density, not variability of the MOC strength.

Consistent with OSNAP observational estimates^13^, overturning at O-West is markedly weaker than at O-East, spans a narrower density range, and peaks at a higher density (Fig. 1b). Turning to the zonally integrated overturning streamfunction, the southward-flowing lower AMOC limb, with waters denser than ${\sigma }_{{moc}}$, reaches a maximum transport of about 24 Sv at 54°N for CESM2 (Fig. 1c).

Low-frequency (LF; multidecadal) streamfunction variability peaks at densities denser than ${\sigma }_{{moc}}\,$(Fig. 1c), consistent with earlier modelling results^15^. This feature is robust across the OSNAP sections (Fig. 1d), individual models (Supplementary Figs. 1 and 2), and an aligned multimodel composite (Supplementary Fig. 3). CESM2 exhibits stronger Labrador Sea variability than many other models and therefore serves only to illustrate the density-space structure of the variability, not as a representative of the ensemble. In CESM2, the LF standard deviation (SD) in O-Full peaks at 2.76 Sv at the O-Full ${\sigma }_{{SD}\max }$ (defined as the density of maximum LF SD), whereas variability at ${\sigma }_{{moc}}$ is only 0.64 Sv, about one-quarter of the peak value. O-West contributes most of this peak, while O-East reaches its own LF SD maximum of about 2.34 Sv at a lighter density. The dominance near ${\sigma }_{{SD}\max }$ therefore reflects strong Labrador Sea overturning variability at these dense densities rather than a dominant contribution of O-West to overall MOC strength variability.

High-frequency (HF; interannual) variability exhibits a similar pattern but with a larger magnitude (Fig. 1e, f). Despite substantial spread in the mean AMOC and its variability across models, a common feature emerges: both LF and HF variability at ${\sigma }_{{moc}}$ are consistently weaker than variability near ${\sigma }_{{SD}\max }$ (Supplementary Figs. 2–4). The contrast shows that the pronounced variability near ${\sigma }_{{SD}\max }$ does not by itself imply strong variability in the overall MOC strength. We next examine more closely the relationship between transport at ${\sigma }_{{SD}\max }$ and MOC variability.

### Impact of strong variability below ${{{\boldsymbol{\sigma }}}}_{{{\boldsymbol{moc}}}}$**on MOC variability**

Given the similar density-space structure of HF and LF variability, we focus here and in the following sections on LF variability and use multimodel results to examine how transport variability at different densities relates to the overall MOC variability. Corresponding HF results are summarized below and shown in the Supplementary Information.

**Fig. 2:**
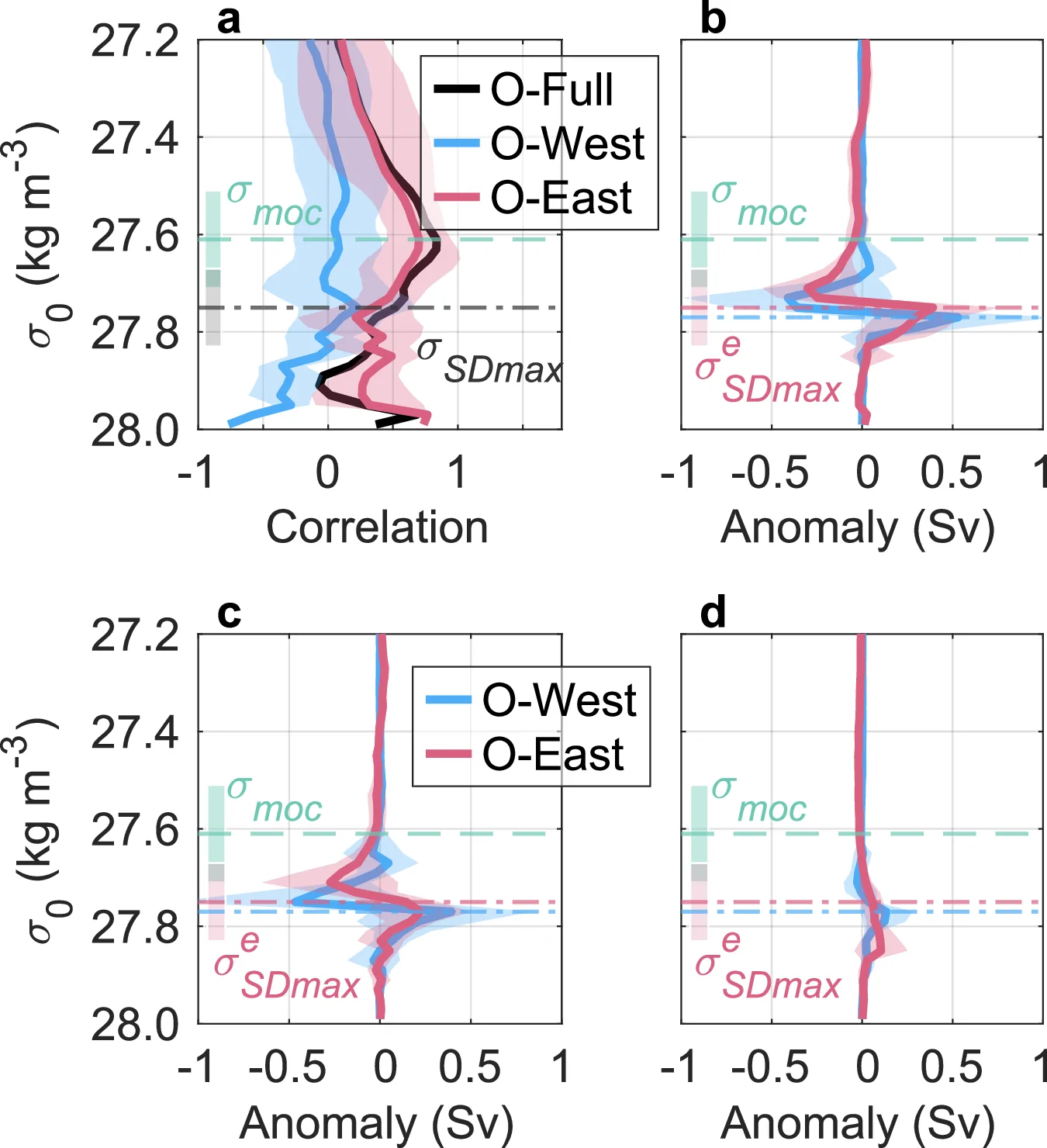
Vertical compensation of low-frequency transport variability and its relationship to the OSNAP MOC. **a** Multimodel mean correlation between the overturning streamfunction at each density and the O-Full MOC, shown separately for O-Full (black), O-West (blue), and O-East (red). The O-Full correlation does not necessarily reach 1 at the multimodel mean ${\sigma }_{{moc}}$, because the MOC is defined as the maximum overturning in density space and the density of this maximum varies in time and across models. **b** Multimodel mean composite transport anomalies on individual density surfaces, constructed by averaging anomalies during periods of positive integrated transport anomaly at ${\sigma }_{{SD}\max }$ in O-East (red) or in O-West (blue). **c**, **d** Composite results as in **b**, but with transport anomalies decomposed into contributions from area anomalies **c** and velocity anomalies **d** on each density surface. In all panels, green dashed line denotes the multimodel mean density of the maximum overturning streamfunction (${\sigma }_{{moc}}$) in O-Full, and black dash–dotted line denotes the multimodel mean density of maximum low-frequency standard deviation (${\sigma }_{{SD}\max }$) of the O-Full streamfunction; colored dash–dotted lines denote the corresponding ${\sigma }_{{SD}\max }$ values for O-East (red) and O-West (blue). Shaded regions indicate ±1 standard deviation across models around the corresponding multimodel mean curves, and vertical bars with matching colors indicate ±1 standard deviation across models in the corresponding characteristic densities.

To identify where in density space transport variability is most strongly linked to overall MOC variability, we calculate the correlation between the full OSNAP MOC and the overturning streamfunction integrated from the bottom to each density level across the OSNAP sections (Fig. 2a). Across models, the correlation peaks near ${\sigma }_{{moc}}$ (around 27.6 kg m⁻³), reaching ~ 0.84 for O-Full and ~ 0.70 for O-East. By contrast, correlations for O-West are weak at ${\sigma }_{{moc}}$ and remain modest across the full density range. At ${\sigma }_{{SD}\max }$ ( ~ 27.75 kg m⁻³ in the multimodel mean), the correlation with the full MOC is substantially lower ( ~ 0.52 for O-Full) than at ${\sigma }_{{moc}}$. Overall, the correlations across density space are dominated by variability in the eastern subpolar basin, with only a limited contribution from O-West, and the strongest correlations occur at ${\sigma }_{{moc}}$.

When transport variability on individual density surfaces is considered, only two models exhibit their maximum correlations with the MOC within one density bin of ${\sigma }_{{SD}\max }$ ($\triangle \sigma$ = 0.02 kg m⁻³; Supplementary Fig. 5), while the remaining models peak at densities either above or below ${\sigma }_{{SD}\max }$. Thus, despite the pronounced variability near ${\sigma }_{{SD}\max }$, variability at this density does not robustly translate into variability of the integrated MOC. This motivates a closer examination of how transport anomalies across density classes combine to shape the integrated overturning.

To reconcile the strong transport variability near ${\sigma }_{{SD}\max }$ with the much weaker variability of the overturning streamfunction at ${\sigma }_{{moc}}$, we construct composites of density-bin transport anomalies (see Methods) by averaging periods when the integrated transport (streamfunction) anomaly at ${\sigma }_{{SD}\max }$ is positive in either O-East or O-West.

The composites reveal a compensating vertical structure in density space: positive transport anomalies near ${\sigma }_{{SD}\max }$ are accompanied by anomalies of the opposite sign at shallower densities (Fig. 2b). When integrated upward, these anomalies partially offset each other, yielding a reduced anomaly near ${\sigma }_{{moc}}$. This compensating structure is evident in both the eastern and western subpolar basins, with comparable contributions to the reduction of the integrated transport anomaly. In the western basin, the compensating structure is shifted toward slightly denser classes relative to the eastern basin, consistent with densification during transit through the Labrador Sea.

To diagnose the nature of this compensating structure across density classes, we decompose transport anomalies into contributions from changes in velocity and cross-sectional area for each density bin (see Methods) and construct analogous composites for each component (Fig. 2c, d). These composites show that opposing transport anomalies across densities are dominated by area-driven contributions in both the eastern and western subpolar basins. The compensation therefore primarily reflects thickening and thinning of adjacent density layers rather than compensating changes in flow speed. As a result, strong variability near ${\sigma }_{{SD}\max }$ mainly represents a redistribution of transport across densities without producing a proportional change in the integrated overturning strength. A similar partitioning between area- and velocity-driven contributions is also found in an analysis of HF variability (Supplementary Fig. 6).

### Linkage between outgoing and incoming transports in the Labrador Sea

Having shown that strong variability near ${\sigma }_{{SD}\max }$ primarily reflects a redistribution of transport across density classes, we next examine how this variability evolves along subpolar circulation pathways, and in particular whether the dense transport anomalies near ${\sigma }_{{SD}\max }$ are transmitted coherently from O-East into and out of the Labrador Sea, or are instead substantially modified within O-West before reaching downstream sections. To do so, we analyze directional density-bin transport anomalies across three connected sections: southward transport across O-East (OE-Out), northward transport into O-West (OW-In), and southward transport exiting O-West (OW-Out). In the primary analysis, these quantities are obtained by integrating transport over the full O-East or O-West section in the specified flow direction within the two density bins immediately denser than ${\sigma }_{{SD}\max }$ specific to each branch, rather than by forming the cumulative density-space integral from the bottom, as used to define the overturning streamfunction.

**Fig. 3:**
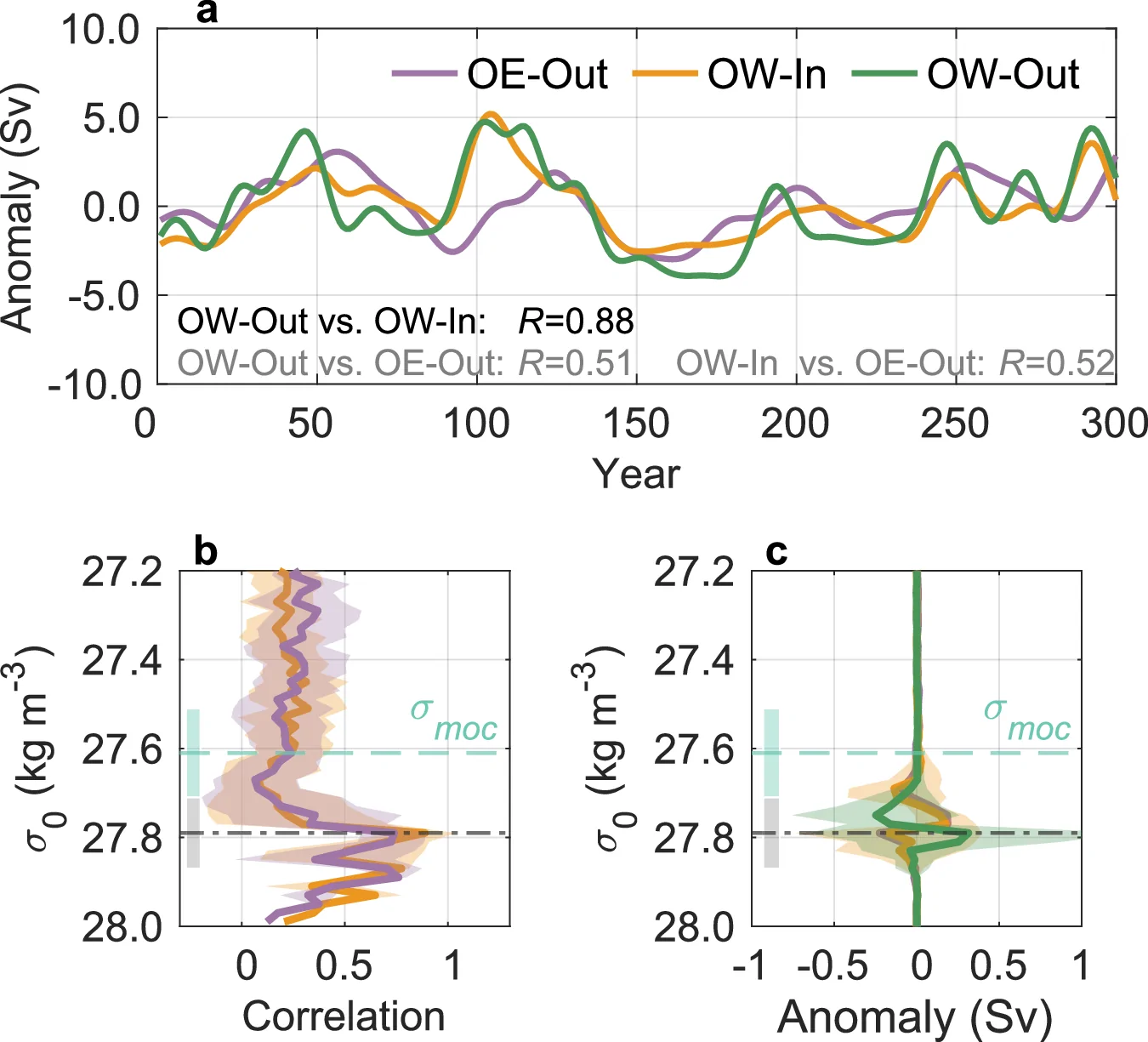
Coherence of transport variability along subpolar circulation pathways. **a** Time series of integrated transport anomalies near ${\sigma }_{{SD}\max }$ for southward transport across O-East (OE-Out), northward transport into O-West (OW-In) and southward transport exiting O-West (OW-Out), shown for the MPI-ESM1-2-HR simulation, selected as a representative example because its OW-Out and OW-In correlation is close to the multimodel mean. Transport anomalies are computed by integrating over the two density bins just below ${\sigma }_{{SD}\max }$. For visualization, the sign of OW-In is reversed to account for its opposite flow direction. Correlation coefficients among the three components are shown at the bottom of the panel. **b** Multimodel-mean correlations of OW-Out transport anomalies at ${\sigma }_{{SD}\max }$ with the corresponding density-bin transport anomalies in OE-Out (purple) and OW-In (orange). **c** Multimodel mean composite transport anomalies on individual density surfaces for OE-Out, OW-In and OW-Out, constructed by averaging anomalies during periods of positive integrated transport anomaly at ${\sigma }_{{SD}\max }$ in each component. Shading denotes ±1 standard deviation across models. The black dash-dotted line denotes the multimodel mean density of maximum low-frequency standard deviation (${\sigma }_{{SD}\max }$) of the streamfunction in OW-Out.

We show results from MPI-ESM1-2-HR (Fig. 3a) because the correlation between OW-In and OW-Out for this model is closest to the multimodel mean. This analysis shows that low-frequency transport anomalies evolve coherently across the three pathway components. In particular, variability in OW-Out (at its ${\sigma }_{{SD}\max }$) is strongly linked to variability in OW-In (at its ${\sigma }_{{SD}\max }$), indicating that anomalies entering the Labrador Sea are clearly expressed in the outgoing flow. This coherence is robust across the multimodel ensemble, with a multimodel mean correlation of 0.87, with correlations ranging from 0.56 to 0.99 across individual models (Fig. 3b). Lagged correlations further show that OW-Out variability at ${\sigma }_{{SD}\max }$ lags OW-In by several months and OE-Out by approximately three years (Supplementary Fig. 7a). For comparison, O-East also exhibits inflow-outflow coherence when OE-In and OE-Out are evaluated at their own ${\sigma }_{{SD}\max }$, but the much weaker correlation (about 0.41) indicates that pathway coherence near ${\sigma }_{{SD}\max }$ is much more strongly expressed in O-West.

As a robustness check, we repeated the pathway analysis using transport integrated from the bottom to the branch-specific ${\sigma }_{{SD}\max }$. This yields the same conclusion and further confirms the contrast between the two basins: the multimodel mean inflow–outflow correlation remains very strong in O-West (0.93) but is still weaker in O-East (0.55). We then used the subset of seven models that provide the variables needed to estimate Labrador Sea surface-forced water mass transformation (SFWMT; Methods) in the dense density range. Using this subset, we compared the extent to which OW-Out variability is linked to upstream inflow versus local surface forcing. We find that OW-Out is more strongly related to OW-In than to SFWMT, with multimodel mean correlations of 0.94 and 0.62, respectively (Supplementary Fig. 8). Consistent with this, OW-In alone also explains substantially more variance in OW-Out than SFWMT alone (multimodel mean *R*^2^ of 0.90 versus 0.46), while including both indicators increases the explained variance only modestly (to 0.92). These results indicate that OW-Out variability is governed primarily by inherited inflow-outflow covariance, with local surface forcing providing a secondary modulation.

The associated composites reveal a consistent density-space structure of transport anomalies across all three sections, characterized by positive transport anomalies at densities slightly denser than ${\sigma }_{{SD}\max }$ and negative anomalies at slightly lighter densities (Fig. 3c). This structure is preserved from OE-Out to OW-Out, although the anomaly amplitude changes along the pathway, consistent with ongoing water mass transformation, either from surface transformation or mixing, during transit. To examine where these modifications are most clearly expressed, we also consider an along-isobath boundary-current version, in which transport is integrated horizontally within 200 km offshore of the 500-m isobath rather than across the full basin. In this boundary-current framework, changes in anomaly amplitude and density structure across the connected sections are more readily apparent (Supplementary Fig. 9).

High-frequency variability shows the same overall behavior, with a similar density-space structure but larger amplitudes (Supplementary Fig. 10), suggesting that the key pathway signature identified here is not restricted to low-frequency variability. Together, these results indicate that transport anomalies entering the Labrador Sea are advected into the outgoing flow and modified during transit, including a modest shift toward denser densities and an amplification of variability.

### Impact on downstream MOC variability

**Fig. 4:**
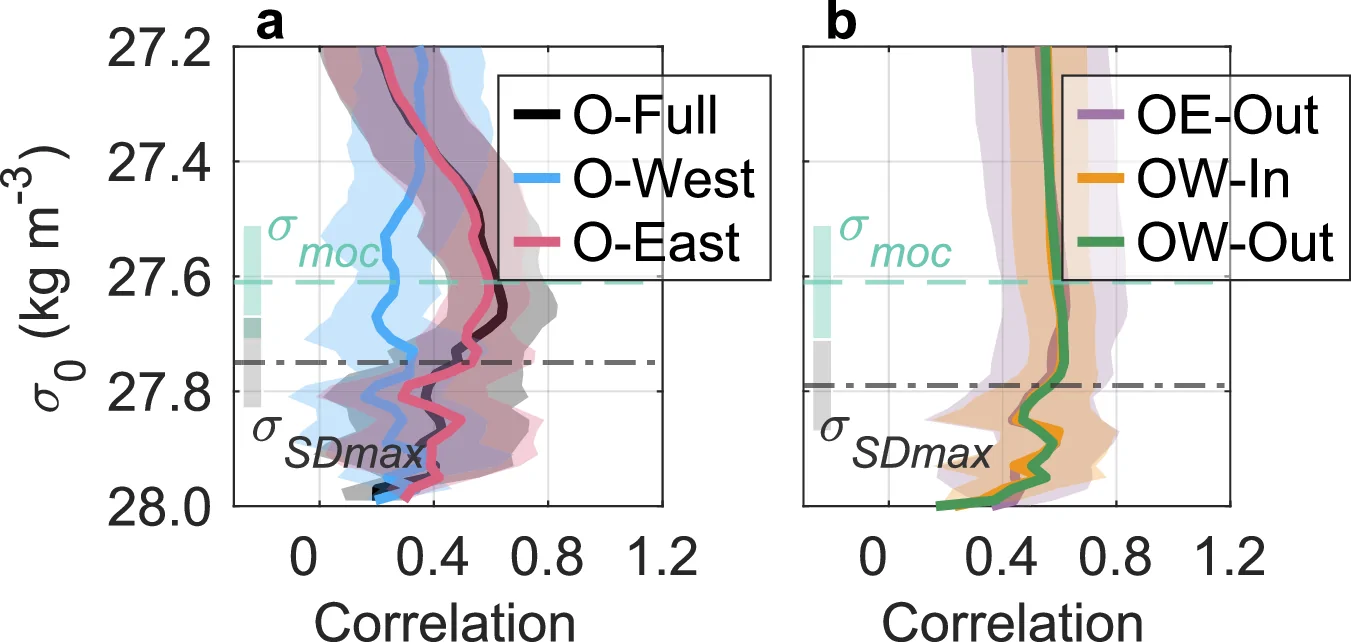
Relationship between subpolar transport variability and downstream MOC. **a** Correlation between the overturning streamfunction at each density across the OSNAP sections and the downstream MOC at 50°N. **b** Same as **a**, but for the OSNAP overturning streamfunction decomposed into its constituent components (OE-Out, OW-In, and OW-Out). The black dash-dotted line indicates the multimodel-mean density of maximum low-frequency standard deviation (${\sigma }_{{SD}\max }$) of the overturning streamfunction for OW-Out, which is slightly denser than the corresponding ${\sigma }_{{SD}\max }$ for O-Full shown in **a**.

Using lead-lag correlations, we next examine how transport variability across the OSNAP sections relates to variability in the downstream MOC. For the basin-integrated overturning streamfunction, O-East exhibits consistently stronger correlations with the downstream MOC across most densities (maximum ~0.59) than O-West (Fig. 4a). O-Full shows a similar structure and a slightly higher maximum correlation ( ~ 0.64), whereas O-West remains weaker ( ~ 0.33).

While our discussion focuses on the downstream MOC at 50°N, these relationships persist over a broader latitudinal range. Correlations between transport variability at the OSNAP sections and the downstream MOC remain consistent from 45°N to 60°N, with correlation strength slightly weakening and associated lead times generally increasing toward lower latitudes (Supplementary Figs. 11a, d).

Decomposition of the western basin overturning into inflow (OW-In) and outflow (OW-Out) components reveals a clearer relationship with the downstream MOC than that inferred from the basin-integrated O-West signal (Fig. 4b). Peak correlations increase to ~0.61 for OW-In and ~0.62 for OW-Out, compared with only ~0.33 for basin-integrated O-West. These directional components also correlate more clearly with the local OSNAP MOC (Supplementary Fig. 7b) and tend to lead the downstream MOC by several years (Supplementary Fig. 11), indicating that basin integration in the Labrador Sea obscures variability that is important for downstream overturning variability.

By contrast, no similar enhancement is found for O-East. The transport exiting O-East (OE-Out) shows a similarly strong relationship with the downstream MOC ( ~ 0.62), but this provides little additional correlation with the downstream MOC beyond the basin-integrated O-East overturning. Together, these results show that, when basin-wide section data are available, O-Full and O-East overturning provide the clearest indicators of downstream MOC variability. O-West becomes similarly informative only after its variability is decomposed into directional components, and even then, OW-In, OW-Out, and OE-Out are broadly comparable rather than showing a unique advantage for Labrador Sea outflow alone. Similar relationships are also found for high-frequency variability, although with systematically weaker correlations (Supplementary Fig. 12).

### Analysis of OSNAP Observations

We now turn to OSNAP data to assess whether the key patterns identified in the model results are also evident in observations. Because the OSNAP record is relatively short (August 2014 to July 2022), the observational analysis is limited to monthly timescales.

**Fig. 5:**
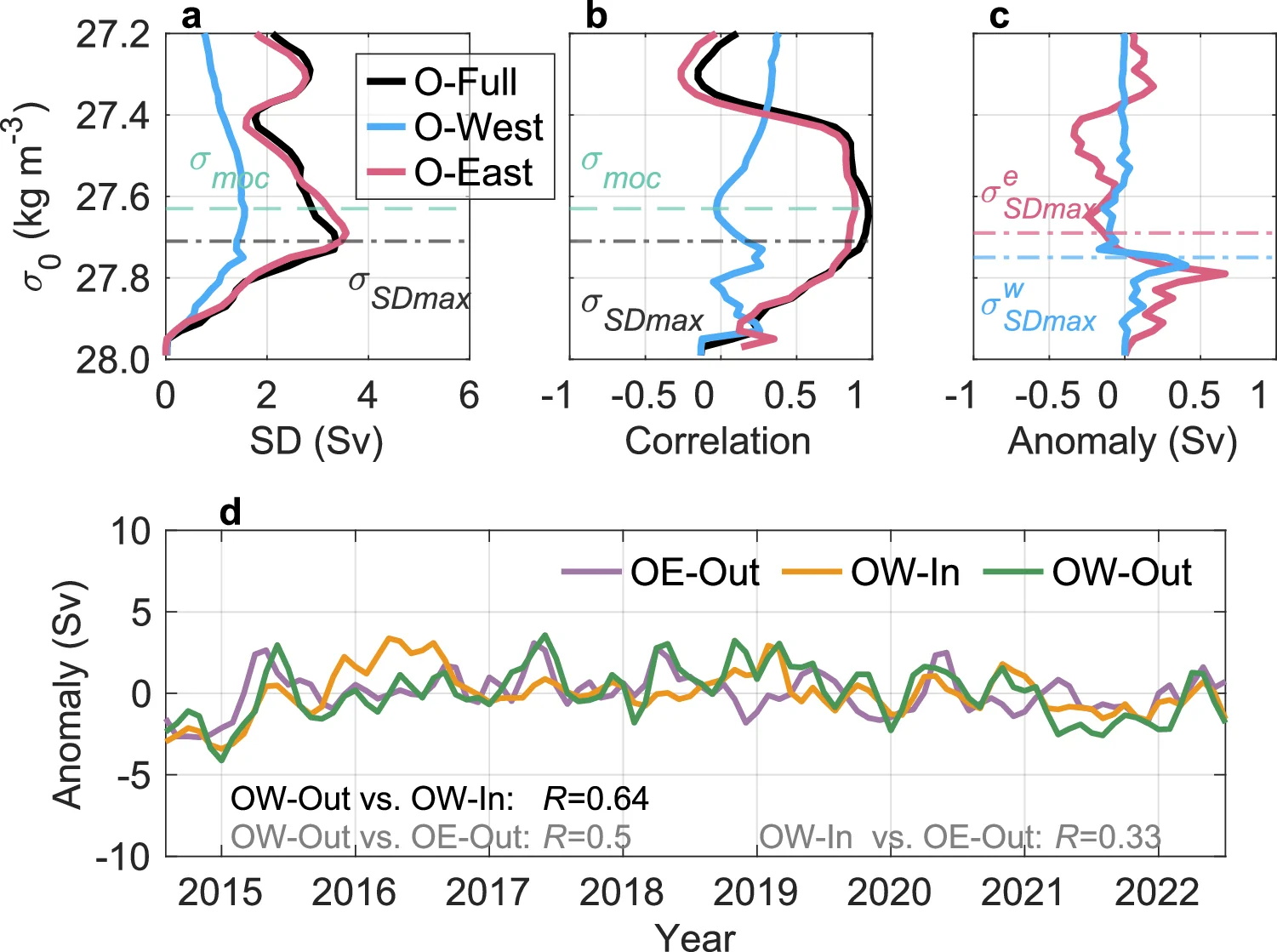
Transport variability from OSNAP observations. **a** Monthly standard deviation (SD) of transport as a function of density across OSNAP sections. **b** Correlation between overturning streamfunction at each density and the OSNAP-Full section MOC (O-Full). **c** Composite mean of transport anomalies on individual density surfaces, constructed by averaging monthly anomalies during periods of positive integrated transport anomaly at ${\sigma }_{{SD}\max }$ in O-East (red) or in O-West (blue). The green dashed line denotes ${\sigma }_{{moc}}$ and the black dash-dotted line denotes ${\sigma }_{{SD}\max }$, both estimate**d** from OSNAP (Methods). **d** Time series of transport anomalies near ${\sigma }_{{SD}\max }$ for southward transport across O-East (OE-Out), northward transport into O-West (OW-In) and southward transport exiting O-West (OW-Out). Transport anomalies are computed by integrating directional transport over the two density bins immediately below ${\sigma }_{{SD}\max }$. For visualization, the sign of OW-In is reversed to account for its opposite flow direction. Correlation coefficients among the three components are indicated at the bottom of the panel. All observational diagnostics are based on the August 2014–July 2022 OSNAP record.

We first examine the density distribution of monthly transport variability across the OSNAP sections. Consistent with the broader framework identified in the model results (Fig. 1 and Supplementary Figs. 2–4), monthly OSNAP transports also exhibit strong variability at densities greater than ${\sigma }_{{moc}}$, and O-East contributes more to the overall MOC monthly variability than O-West (Fig. 5a, b). Unlike in CESM2 (Supplementary Fig. 13), however, observed monthly variability in O-West is more evenly distributed across densities, and O-East is more variable than O-West at all densities. In CESM2, by contrast, variability in O-West is much stronger and is more concentrated in the dense classes.

A composite analysis of monthly transport anomalies reveals a vertical compensating structure in both O-West and O-East (Fig. 5c), characterized by anomalies of opposite sign across adjacent density classes. Compared to the multimodel low-frequency results (Fig. 2b), this compensation is more broadly distributed in density space and leads to weaker cancellation when transport is integrated upward to ${\sigma }_{{moc}}$. As a result, monthly variability projects more strongly onto the integrated overturning, consistent with the enhanced short-timescale variability observed in the OSNAP MOC. A similar compensating structure is also found along subpolar circulation pathways and is most clearly expressed in the outgoing transport from the western basin (OW-Out), while being weaker and less organized in OE-Out and OW-In (Supplementary Fig. 14).

Finally, time series of transport anomalies evaluated near the density of maximum variability for each component show that OE-Out, OW-Out, and OW-In exhibit coherent variability on monthly timescales (Fig. 5d). In particular, the observed correlation between OW-Out and OW-In ( | *R* | = 0.65; sign reversed for visualization) is comparable to that found in the model results on monthly (0.73; Supplementary Fig. 13d), HF (0.71) and LF (0.87) timescales. This supports the interpretation that variability entering the Labrador Sea is reflected in the outgoing transport, even on short timescales.

Together, the OSNAP observations support the key elements of the model-based picture. On monthly timescales, transport variability exhibits partial compensation across adjacent density classes, although this compensation is more broadly distributed in density space than in the low-frequency model results. The eastern subpolar basin contributes most to monthly OSNAP MOC variability, and transport exiting the Labrador Sea remains strongly linked to upstream variability though modified during transit through the basin. These results indicate that, although the relative importance of individual processes varies with timescale, the structural relationships among density-space variability, pathway coherence, and overturning variability are consistent between models and observations.

## Discussion

A key outcome of this study is that, although transport variability in density space peaks at a density (${\sigma }_{{SD}\max }$) denser than the density of maximum overturning (${\sigma }_{{moc}}$), this pronounced variability is only weakly related to fluctuations in local and downstream overturning strength. Similar separations between the depth or density of maximum overturning transport and that of maximum variability have been reported across a wide range of latitudes and models^15,21^. Here we show that this separation persists from monthly to multidecadal timescales in both climate models and OSNAP observations. Pronounced dense variability in the western subpolar North Atlantic should therefore not be interpreted as a direct proxy for AMOC strength.

We attribute this weak linkage to substantial transport compensation within the AMOC lower limb. Composite analyses show that enhanced transport anomalies near ${\sigma }_{{SD}\max }$ are accompanied by anomalies of the opposite sign at adjacent densities, thus reducing their net contribution when transport is integrated across the densities that comprise the lower limb. Decomposition of the transport anomalies indicates that this compensation is driven primarily by changes in layer thickness rather than by compensating flow speed anomalies. This strong thickness compensation essentially reflects the redistribution of transport within the lower limb without producing proportional changes in the overturning strength. The thickness-driven compensation surrounding ${\sigma }_{{SD}\max }$ could be related to strong variability in the surface outcrop area of those densities.

Across the model ensemble, variability in the eastern subpolar basin exhibits stronger correlations with both the OSNAP MOC and the downstream MOC than variability in the western basin across most density classes and timescales. This result is consistent with previous studies highlighting the dominant role of the Irminger and Iceland basins in shaping the AMOC mean state and variability^13,22^.

Although basin-integrated overturning in the western subpolar basin exhibits only weak correlations with the downstream MOC in the multimodel mean, decomposing the western (Labrador) basin into its northward inflow and southward outflow components reveals a markedly stronger relationship. This contrast arises because inflow and outflow anomalies are strongly correlated yet opposite in sign, so their lateral integration across the basin leads to partial cancellation, contributing to the weak relationship between basin-integrated western overturning and overall MOC variability. At the same time, the broadly similar correlations of OE-Out, OW-In, and OW-Out with the downstream AMOC indicate that variability most relevant to downstream overturning is already present in the transport exiting OSNAP East. As shown in previous studies, the signal exiting OE-Out mainly reflects buoyancy-forced transformation in the Iceland and Irminger basins, as well as the broader eastern subpolar basin^23,24^. This variability is subsequently advected into the Labrador Sea, where it is modified, often amplified and shifted toward denser classes during transit^25^. Despite these modifications, its relationship with the downstream AMOC is largely unchanged from that exiting OSNAP-East. This interpretation is supported by the comparison between OW-In and Labrador Sea SFWMT: OW-In explains substantially more variance in OW-Out than SFWMT, and including both increases explained variance only modestly. Consistent with recent work suggesting that dense waters exported from the Labrador Sea reflect cumulative transformation across the broader subpolar gyre, rather than formation within a single localized region^26–28^, these results indicate that the Labrador Sea acts primarily as a conduit for transport anomalies linked to AMOC strength variability. This conduit interpretation refers specifically to the incoming and exported Labrador Sea transport anomalies, which are connected to transport variability exiting OSNAP-East and are significantly correlated with downstream and OSNAP-Full MOC strength. It does not imply that the local Labrador Sea overturning residual is inherited directly from upstream. OSNAP observations, despite their limited duration and restriction to monthly timescales, support this interpretation by revealing similar compensating structures and strong correlations between incoming and outgoing transports across OSNAP-West.

This framework helps reconcile our results with previous studies emphasizing the importance of Labrador Sea water mass transformation for downstream AMOC variability^15^. Our results do not argue against such a contribution. Rather, they suggest that across the broader model ensemble, Labrador Sea SFWMT explains only part of the downstream signal. That downstream signal, expressed as AMOC variability at 50°N, is more consistently reflected in the upstream transport anomalies (OE-Out, OW-In, and OW-Out) than in SFWMT. Thus, although Labrador Sea transformation can modify the dense signal, it does not emerge as the dominant or most robust control on downstream AMOC variability across models. This distinction is important for identifying indicators of the downstream MOC. When basin-wide section data are available, O-East and O-Full provide the clearest measures of downstream AMOC strength, whereas decomposing Labrador Sea overturning into inflow and outflow components helps recover the downstream-relevant variability that is obscured in the basin-integrated western overturning in the Labrador Sea.

These conclusions should be interpreted with awareness of the limitation of CMIP6 climate models in the subpolar North Atlantic. Models differ substantially in stratification, overflow representation, and the location and intensity of deep convection and water mass transformation, particularly in the Labrador Sea, leading to large inter-model spread in density structure and variability^26,28–30^. Our aim is therefore not to quantify absolute overturning or water mass transformation in any individual region, nor to attribute all model differences to a single mechanism. Instead, it focuses on the structural relationship between transport variability across density classes and integrated overturning that persist across models and are also evident in observations. Across the CMIP6 ensemble, key features, such as strong variability in dense waters, compensation across density classes, coherent pathway evolution, and the weak projection of dense variability onto overturning strength, emerge despite model spread. Similar structural signatures are also evident in OSNAP observations on monthly timescales, lending observational support to the mechanisms identified here. In that sense, the value of the multimodel framework here is not to provide a single deterministic picture, but to identify which features emerge robustly despite large differences in model mean state and variability.

An important implication of our results is that diagnosing AMOC variability based on transport at a single density or depth can be misleading. Variability below ${\sigma }_{{moc}}$ may appear prominent, yet we show here that its relationship to local or downstream overturning strength can be tenuous when compensation across density classes is strong. This perspective clarifies why studies focusing on lower-limb variability or transformation often identify pronounced signals that show limited correspondence with overturning metrics defined by the maximum streamfunction. Extending sustained observations such as OSNAP will be essential for further constraining how these structural relationships evolve on longer timescales and for anchoring model performance in simulating AMOC variability.

## Methods

### Calculation of the MOC

The meridional overturning circulation (MOC) streamfunction in density space is calculated as:

1 $$\Psi \left(\sigma \right)=\,-\,\int _{{x}_{w}}^{{x}_{e}} \int _{{\sigma }_{\max }}^{\sigma }v\left(x,\,\sigma {\prime} \right) \, d\sigma {\prime} \, {dx},$$

where ${x}_{w}$ and ${x}_{e}$ represent the western and eastern endpoints of the section, respectively, σ is density, and ${\sigma }_{\max }$ denotes the maximum density. For the streamfunction across each OSNAP section, used with both model output and observations, v represents the velocity perpendicular to the section (positive northward). For the streamfunction across zonal sections, used with model output, v represents the meridional velocity. Model velocity and hydrographic fields are interpolated onto the OSNAP grid, and potential density referenced to the surface, ${\sigma }_{0}$, is then calculated offline using the Gibbs Seawater (GSW) Oceanographic Toolbox^31^. In practice, the streamfunction is computed on discretized density levels with a uniform bin width of $\triangle \sigma$ = 0.02 kg m⁻³. The MOC (in Sv, 1 Sv = 10^6^ m^3^ s^-1^) is defined as the maximum of the overturning streamfunction in density coordinates, and the density at which that maximum occurs is denoted as ${\sigma }_{{moc}}$. In our study, we also identify the isopycnal at which the standard deviation of the overturning streamfunction is maximized and refer to that as ${\sigma }_{{SD}\max }$. Because the streamfunction is defined as the cumulative transport integrated upward from the bottom, the streamfunction value at ${\sigma }_{{SD}\max }$ corresponds to the transport integrated from the bottom to ${\sigma }_{{SD}\max }$. This diagnostic is used consistently throughout the study to characterize the density level associated with the strongest transport variability.

To examine transport variability prior to vertical integration, we also analyze transport on individual density surfaces (referred to as density-bin transport in this study). For a given density, the transport is defined as

2 $$V\left(\sigma \right)=- \int _{{x}_{w}}^{{x}_{e}}v\left(x,\,\sigma \right) \, {dx},$$

which represents the along-section transport associated with that density surface. By construction, the overturning streamfunction $\Psi \,\left(\sigma \right)$ corresponds to the cumulative vertical integral of $V\left(\sigma \right)$ from ${\sigma }_{\max }$ to σ. This relationship allows us to distinguish variability that is prominent on individual density surfaces from variability that contributes to the integrated overturning.

Building on the definition, we diagnose the origin of compensating transport anomalies across density classes by decomposing density-bin transport anomalies into contributions associated with velocity and cross-sectional area variations. For each density bin, transport can be expressed as

3 $$V\left(\sigma \right)=\,A\left(\sigma \right)\,\bar{v}\,\left(\sigma \right)+\bar{A}\left(\sigma \right)\,v{\prime} \,\left(\sigma \right)+A{\prime} \left(\sigma \right)\,v{\prime} \left(\sigma \right),$$

where A denotes the cross-sectional area of the density layer, v the velocity normal to the section, overbars indicate time means, and primes indicate anomalies. In practice, the transport anomaly is approximated by the sum of the velocity-driven component ${V}_{u}$ ($\bar{A}v{\prime}$) and the area-driven component ${V}_{A}$ ($A{\prime} \bar{v}$), with the small nonlinear term $A{\prime} v{\prime}$ included in the residual.

### Temporal filtering

For all model simulations, monthly output from the first 300 years of the preindustrial control (piControl) integrations is used. Annual means are computed from the monthly data prior to filtering. Low-frequency (LF) variability is isolated using a fourth-order Butterworth low-pass digital filter with a 20-year cutoff period, while high-frequency (HF) variability is obtained using a Butterworth high-pass filter with a 10-year cutoff. Sensitivity tests using alternative cutoff periods (e.g. 10 and 15 years for the low-pass filter) yield qualitatively similar results and do not affect the main conclusions. For OSNAP observations, the shorter record length limits the analysis to monthly timescales. No temporal filtering is applied unless explicitly stated.

For diagnostics that depend on variability amplitude in density space, including the identification of ${\sigma }_{{SD}\max }$, all calculations are performed on temporally filtered, mean-removed time series.

### Composite analysis and decomposition

Composite analyses are used to diagnose the vertical structure of transport anomalies associated with strong variability near ${\sigma }_{{SD}\max }$. For each model (and for OSNAP observations), anomalies are defined from the temporally-filtered variability on the timescale of interest. Periods are then selected when the anomaly of the integrated overturning streamfunction at ${\sigma }_{{SD}\max }$ in O-West or O-East is positive. Anomalies in the quantity of interest (e.g. integrated streamfunction, density-bin transport, or its area- and velocity-driven components) are then averaged over these selected periods to form the composite.

In addition to basin-integrated diagnostics, we also analyze directional transport anomalies across three connected sections: southward transport across O-East (OE-Out), northward transport into O-West (OW-In), and southward transport exiting O-West (OW-Out). In the primary analysis, these quantities are defined by selecting velocity according to flow direction and integrating transport across the full O-East or O-West section within the specified density range near ${\sigma }_{{SD}\max }$. As a robustness check, the same pathway analysis is repeated using transport cumulatively integrated from the bottom to the branch-specific ${\sigma }_{{SD}\max }$.

All composites are constructed from the filtered LF or HF time series, depending on the application described in the main text.

### Alignment across models in density space

Because the density at which maximum variability occurs differs among models, direct averaging of profiles in density space can artificially smooth or obscure coherent structures (Supplementary Figs. 2, 4). To reduce this effect, composite and correlation profiles, and streamfunction fields from each model are shifted in density space so that their respective ${\sigma }_{{SD}\max }$ aligns with the multimodel-mean ${\sigma }_{{SD}\max }$ prior to averaging. This procedure preserves the relative structure of variability around the density of maximum variance while minimizing inter-model smearing. This alignment is performed prior to all density-dependent multimodel averaging.

### Correlation analyses

Correlations between transport variability and MOC variability are computed using Pearson correlation coefficients. Unless otherwise stated, correlations are calculated at zero lag using anomalies of the same temporal filtering (LF, HF, or monthly). Correlations are computed between:

(i) the MOC time series and the overturning streamfunction $\varPsi \left(\sigma \right)$ at each density;
(ii) the MOC and density-bin transport $V\left(\sigma \right)$; and
(iii) transport time series across different OSNAP sections (e.g. OE-Out, OW-In, OW-Out) with streamfunction of OW-Out at ${\sigma }_{{SD}\max }$.

To examine temporal relationships between upstream and downstream variability, lead–lag correlations are computed throughout this study. In these cases, correlations are evaluated over a range of positive and negative lags, with negative lags indicating subpolar transport leading the downstream MOC. The maximum correlation across all lag times is reported, and the corresponding lead-lag times vary among models and are shown in the Supplementary Information (e.g., Supplementary Fig. 11). Correlation profiles are first calculated independently for each model, then aligned in density space (with respect to ${\sigma }_{{SD}\max }$) before computing the multimodel mean.

### Surface-forced water mass transformation

Surface-forced water mass transformation (SFWMT) was computed offline in density space following the standard Walin framework and using the same formulation as in previous studies of subpolar overturning variability^15,23^. Briefly, monthly surface heat and freshwater fluxes were combined with surface temperature and salinity to obtain the surface density flux, which was then integrated over the outcrop area of each density class to yield transformation as a function of density. The calculation used the monthly model fields hfds, wfo, tos, and sos, and was performed on the same density grid as the overturning analysis ($\Delta \sigma$ = 0.02 kg m^-3^). Positive values indicate transformation toward denser classes. Because only seven models provide the full set of required surface variables, all comparisons between SFWMT and transport variability are based on this seven-model subset.

### Observations and models

Observational estimates of the overturning circulation are based on data from the Overturning in the Subpolar North Atlantic Program (OSNAP). We use eight years of monthly OSNAP observations spanning August 2014 to July 2022, covering the full OSNAP section (O-Full) and its two coast-to-coast subsections, O-West and O-East (Fig. 1a). The OSNAP observing system provides gridded property and velocity fields, MOC and other time series across the section, derived from a combination of moored instruments, gliders, and ship-based measurements. The processing steps include quality control, interpolation to a common grid, and the calculation of transports in density coordinates, following established methodologies^32^. We use the gridded temperature, salinity, and velocity products to compute the overturning streamfunction and associated transport diagnostics in density space, consistent with previous OSNAP-based studies^19^.

We analyze simulations from nine coupled climate models participating in CMIP6 together with one high-resolution coupled model (CESM1.3-HR), using 300 years from each preindustrial control simulation to isolate internally generated variability. The full list of models and their key characteristics are provided in Supplementary Table 1.

The resulting ensemble spans six distinct ocean model formulations, with two model pairs differing primarily in horizontal resolution, providing a range of subpolar circulation representations without targeting specific processes. The nine CMIP6 models were drawn from the CMIP6 archive based on the availability of long preindustrial control simulations and the three-dimensional fields required for density-space diagnostics. We used Heuzé et al.^29^ as a broad reference to exclude models with extreme bottom-density biases in the Greenland–Iceland–Norwegian Seas and subpolar gyre, which could possibly dominate density-space structure and hinder inter-model comparison. Beyond this exclusion of clear outliers, no selection was made based on overturning behavior. Thus, the final ensemble represents a quasi-random subset spanning a range of ocean model formulations and horizontal resolutions. Accordingly, our analysis does not rely on the fidelity of any individual model, but instead emphasizes relationships that are consistent across models, timescales, and independent observations.

To ensure consistency with observational diagnostics, model output is first interpolated onto the OSNAP section grid, and velocity components are rotated to obtain the section-normal velocity. The overturning streamfunction and associated transport metrics are then computed using the same methodology as applied to OSNAP observations.

While our primary results are based on multimodel statistics, two individual models are used in selected figures for illustration. MPI-ESM1-2-HR is shown in Fig. 3a because the correlation between OW-Out and OW-In in this model is close to the multimodel mean, making it a representative example of the ensemble behavior. CESM2 is used in selected figures to illustrate the mean overturning structure and spatial patterns of variability. Neither model is chosen for superior performance; rather, the qualitative behaviors highlighted are representative of those found across the ensemble.

## Supplementary information

Supplementary Information

Transparent Peer Review file

## Data Availability

The CMIP6 pre-industrial control (piControl) model output used in this study, including thetao, so, uo, and vo from the r1i1p1f1 ensemble member, is publicly available through the Earth System Grid Federation (ESGF; https://metagrid.esgf-west.org/search/cmip6/). The models and dataset specifications are listed in Supplementary Table 1. The CESM1.3-HR pre-industrial control simulation is publicly available through the International Laboratory for High-Resolution Earth System Prediction (iHESP; https://ihesp.github.io/archive/products/ds_archive/Datasets.html). The OSNAP gridded temperature, salinity, and velocity fields for 2014–2020 are publicly available at https://doi.org/10.35090/gatech/70342. The processed data underlying the figures are publicly available at https://github.com/jchenocean/North-Atlantic-AMOC-1Variability. Source data are provided with this paper.
