# Supplementary material for: The Labrador Sea as a conduit for AMOC lower-limb transport anomalies on multiple timescales
Source: Nat Commun. 2026 Aug 28;17:10298. doi: 10.1038/s41467-026-77271-z (PMC13620149; doi:10.1038/s41467-026-77271-z)
Supplement: Supplementary file 1 [file 41467_2026_77271_MOESM1_ESM.pdf]

Supplementary Information for

The Labrador Sea as a conduit for AMOC lower-limb transport anomalies on multiple timescales

Jia-Jia Chen*, M. Susan Lozier

*Corresponding author. Email: jiajiachen@gatech.edu

This PDF file includes:

Supplementary Figures 1–14
Supplementary Table 1
References (1–10)

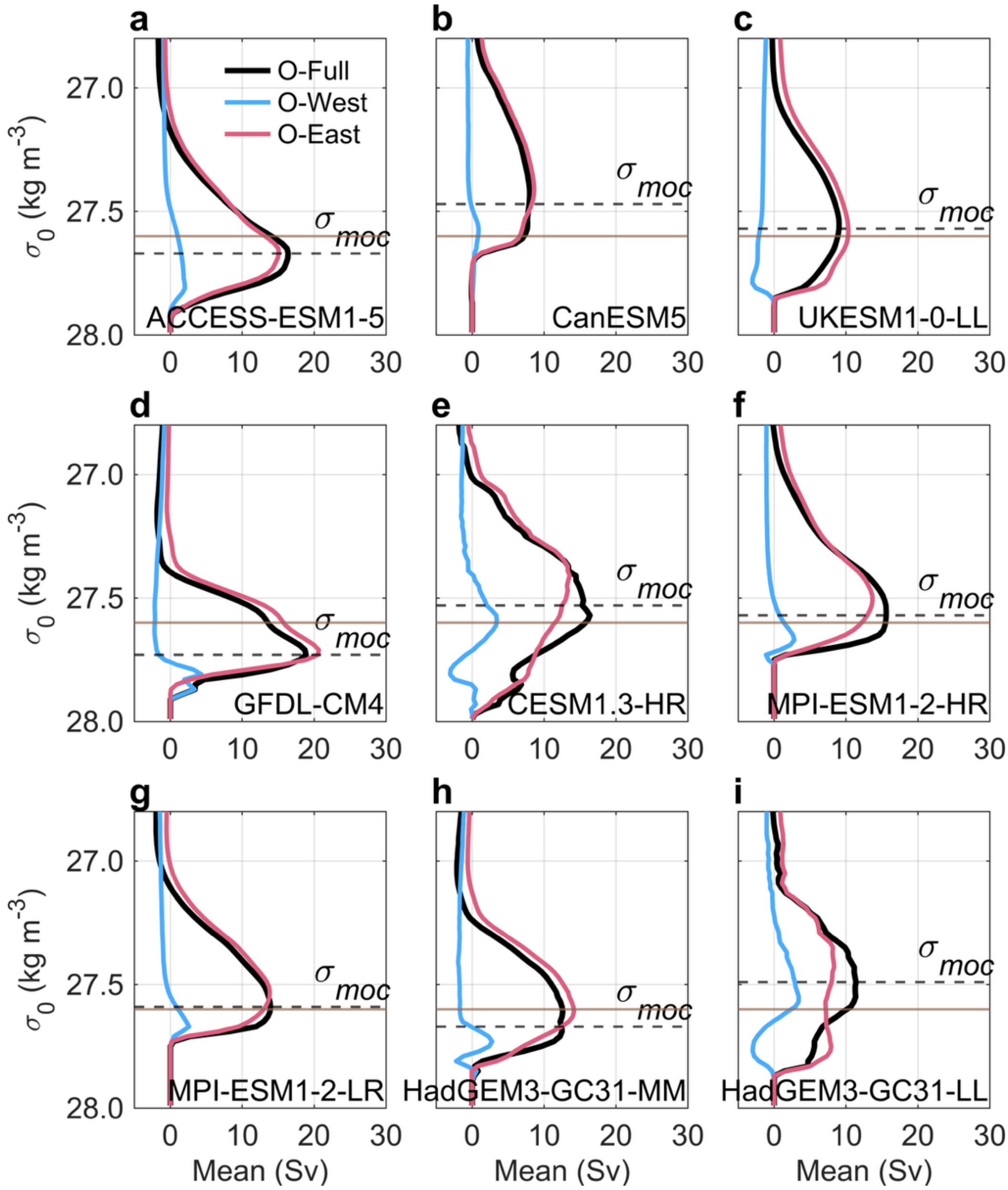

Supplementary Figure 1. Time-mean overturning streamfunction in density space derived from the suite of CMIP6 models used in this study. The black dashed lines indicate the density of maximum overturning σ_{moc} in each individual model, whereas the brown solid line marks the multimodel-meanσ_{moc}. Shown are the same diagnostics as in Fig. 1b of the main text, but for the remaining nine models in the ensemble.

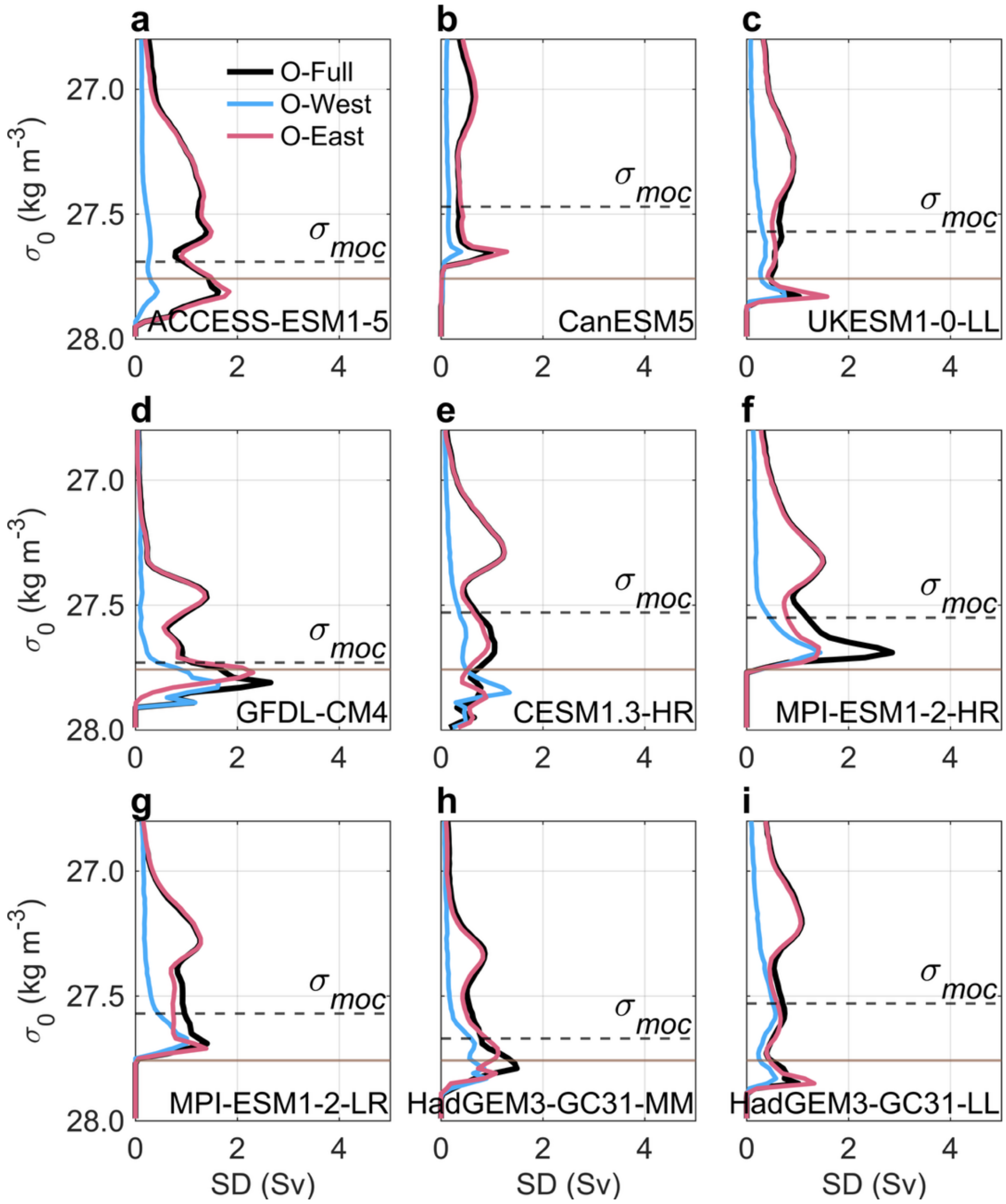

Supplementary Figure 2. Low-frequency (LF) variability of overturning stream function in density space derived from the suite of CMIP6 models used in this study. The black dashed lines indicate the density of maximum overturning σ_{moc} in each individual model, whereas the brown line marks the multimodel-mean density of maximum LF standard deviation (SD) σ_{SDmax}. Shown are the same diagnostics as in Fig. 1d of the main text, but for the remaining nine models in the ensemble.

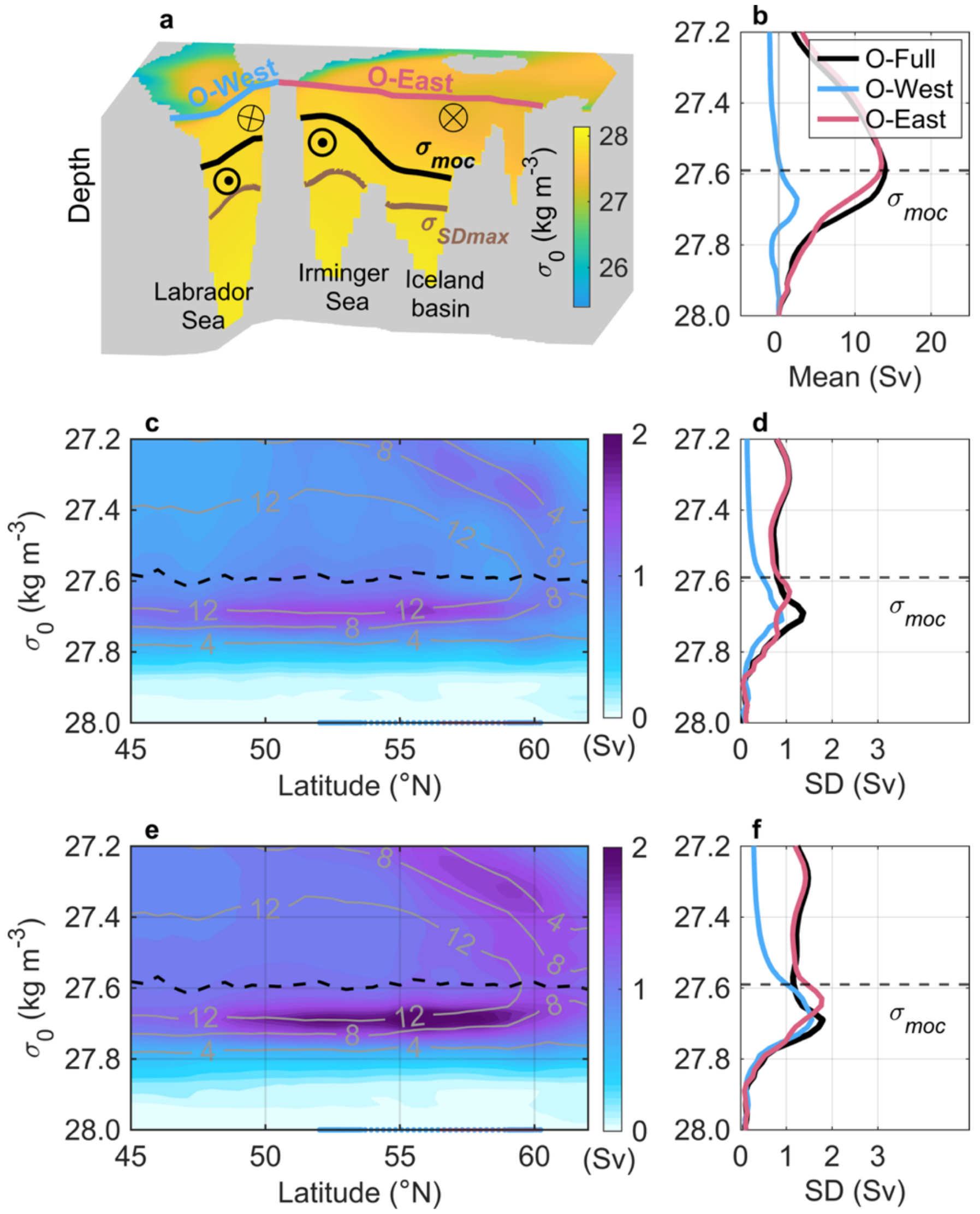

Supplementary Figure 3. Aligned multimodel composite of the mean and variability of the overturning streamfunction in density space. a Schematic diagram of O-West and O-East sections and frequently used isopycnals in this study (σ_{moc} and σ_{SDmax}). σ_{moc} (black line) is the density of the maximum streamfunction for O-Full, and σ_{SDmax} (brown line) is the isopycnal of the maximum standard deviation (SD) of the streamfunction for O-Full. Circles with crosses and dots represent northward and southward flows, respectively. **b** Aligned multimodel mean time-mean overturning streamfunction across the three sections indicated. **c** Latitude-density cross-section of the time-mean overturning streamfunction (gray contours) and low-frequency (LF; variability on timescales > 20 years) SD (colored shading). Before averaging, each model is mapped onto a common density coordinate relative to its own σ_{moc}. The SD shown here represents variability of the overturning streamfunction at each density, not variability of MOC strength, which is defined as the maximum of the density-space overturning streamfunction at σ_{moc}. The black dashed line denotes values of σ_{moc} for each latitude. Blue and red dots alone the x-axis indicate the latitudinal positions of the O-West and O-East arrays, respectively. For reference, the OSNAP array spans approximately 52–60°N. **d** LF SD of the streamfunction across the three sections indicated. **e**, **f** Same as **c**, **d**, but for high-frequency (HF; variability on timescale < 10 years) SD. All quantities are computed from the 300-year simulations and then averaged across models using the density-alignment procedure described in Methods.

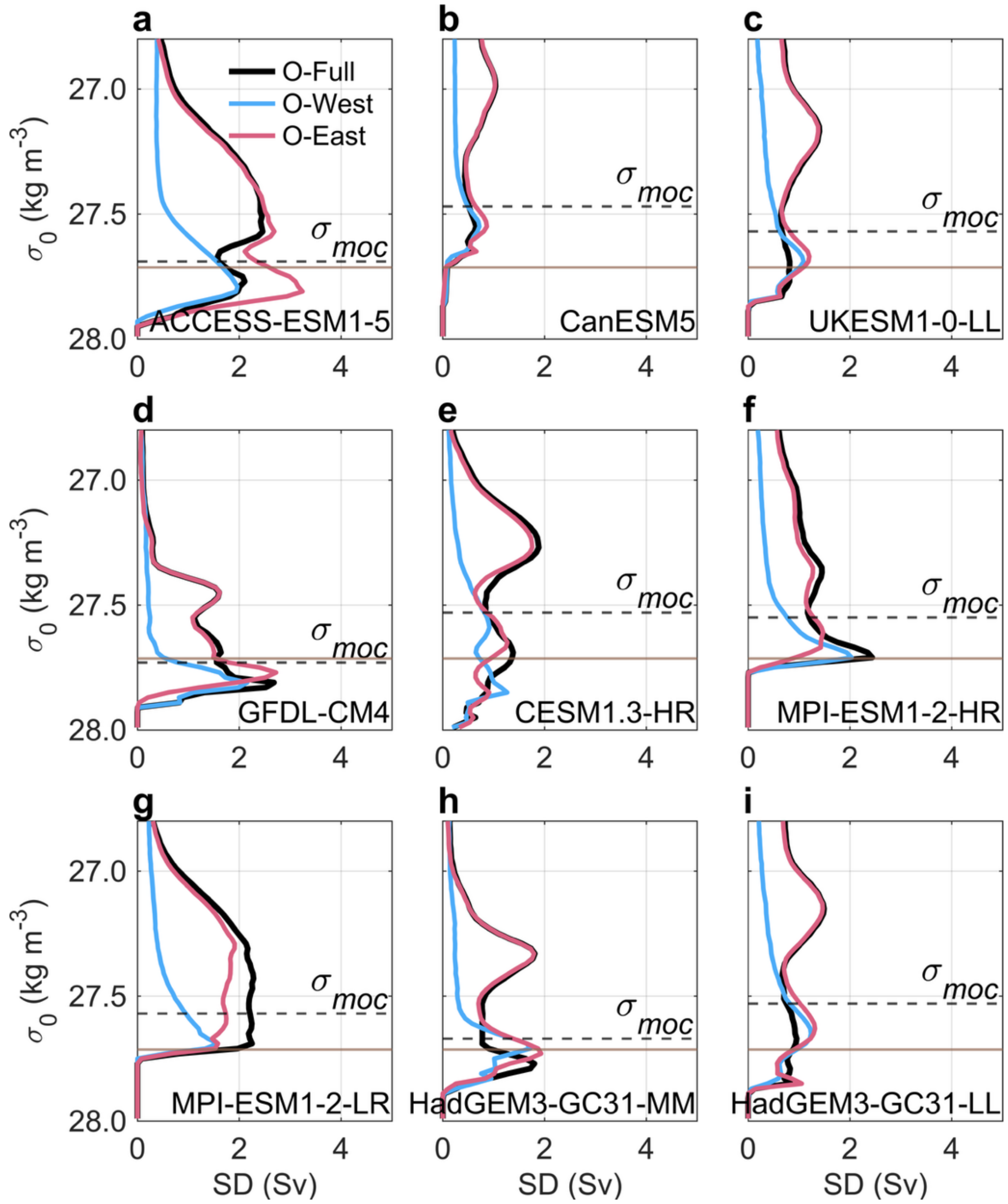

Supplementary Figure 4. High-frequency (HF) variability of overturning stream function in density space derived from the suite of CMIP6 models used in this study. The black dashed lines indicate the density of maximum overturning σ_{moc} in each individual model, whereas the brown line marks the multimodel-mean density of maximum HF standard deviation (SD) σ_{SDmax}. Shown are the same diagnostics as in Fig. 1f of the main text, but for the remaining nine models in the ensemble.

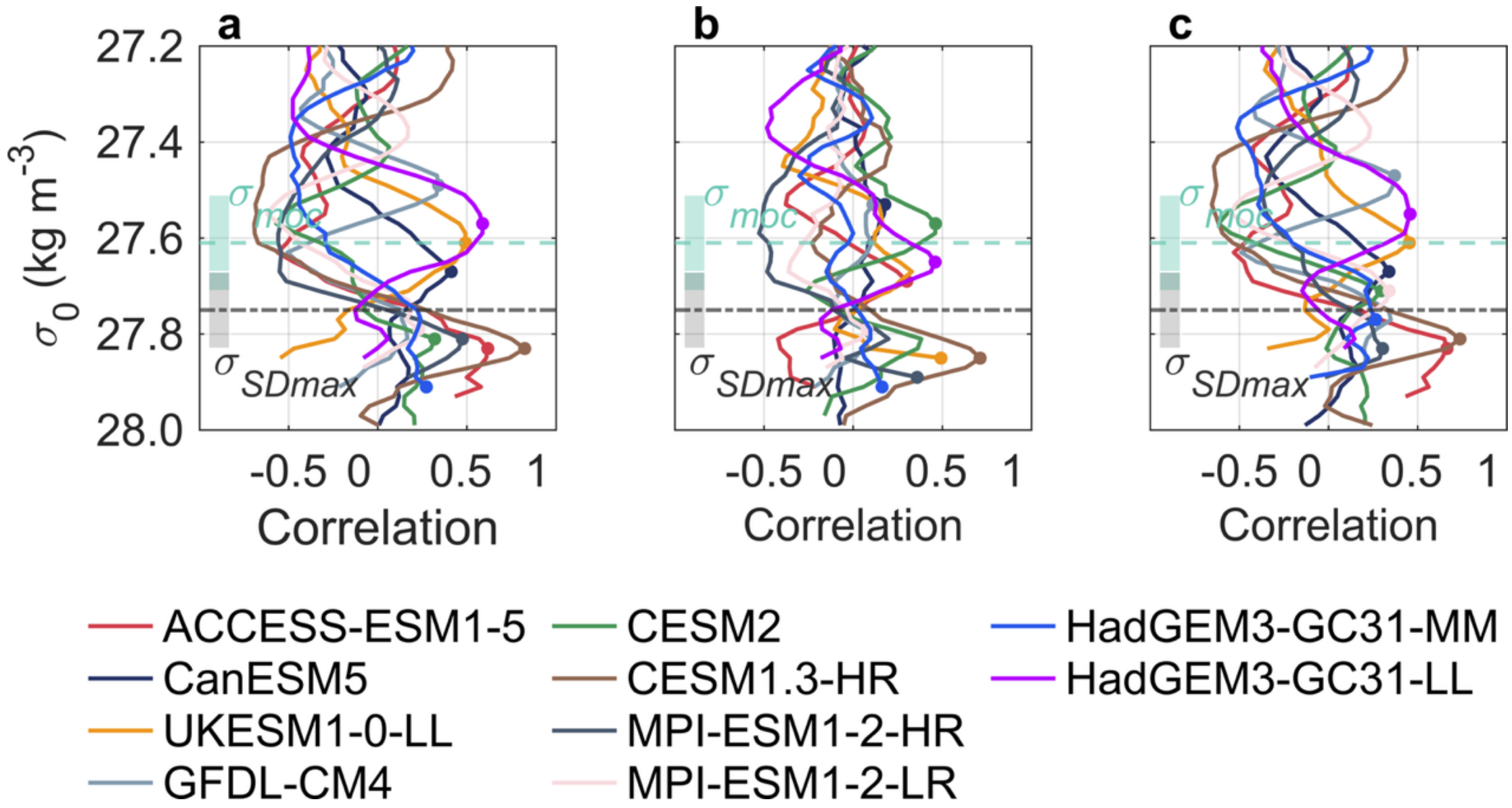

Supplementary Figure 5. Model-by-model correlations between transport variability and the OSNAP MOC. a–c Correlation between density-bin transport anomalies and the OSNAP full-section MOC (O-Full), shown separately for O-Full (a), O-West (b), and O-East (c).

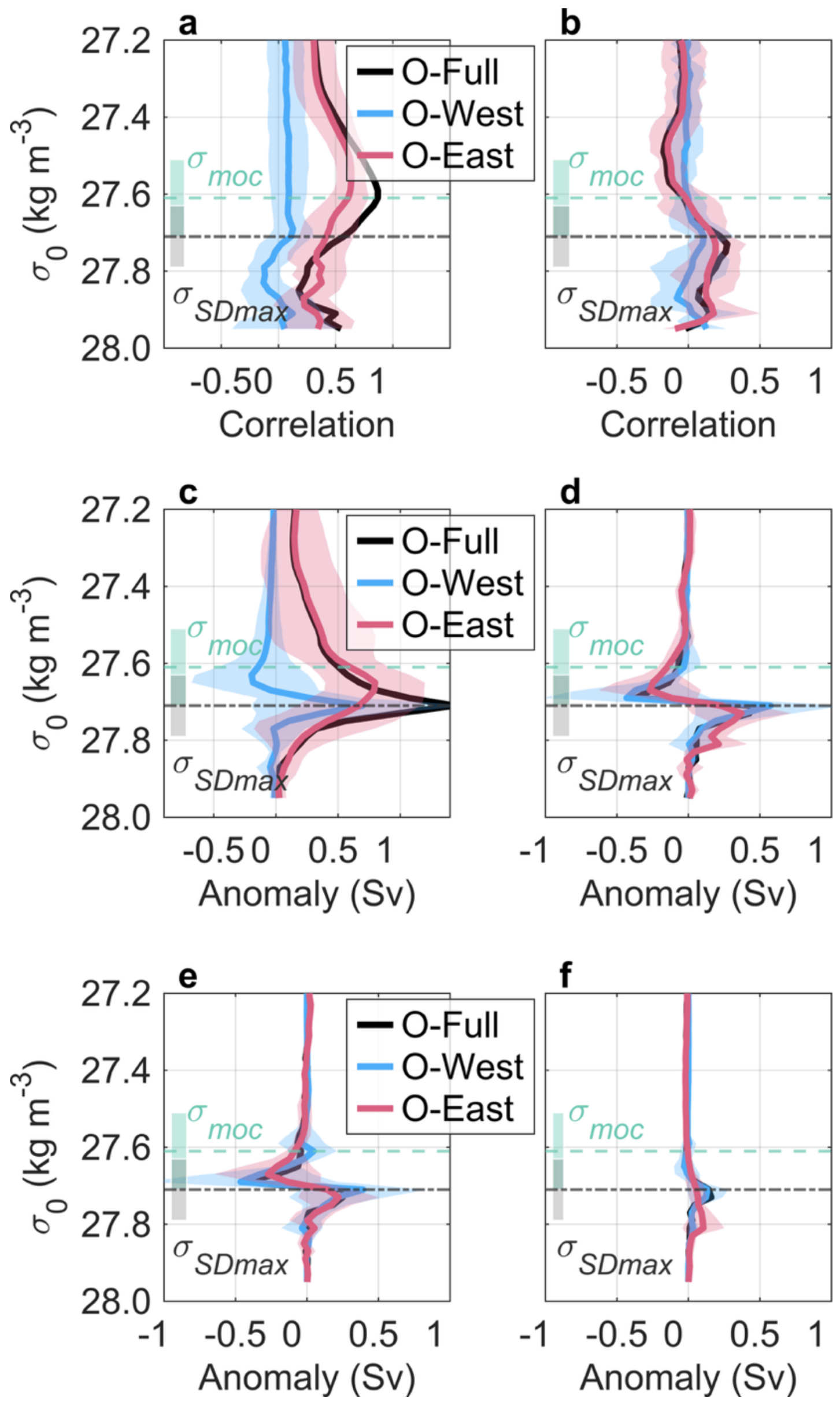

Supplementary Figure 6. Vertical compensation of high-frequency transport variability and its relationship to the OSNAP MOC. Similar to Fig. 2 of the main text, but for high-frequency (HF; interannual) variability. **a** Correlation between the overturning streamfunction at each density and the OSNAP-Full section MOC (O-Full), shown separately for O-Full (black), O-West (blue), and O-East (red). **b** As in a, but for transport anomalies on individual density surfaces (density-bin transport). **c** Composite mean of integrated transport (streamfunction) anomalies as a function of density, constructed by averaging anomalies during periods when the integrated transport anomaly at σ_{SDmax} in O-Full is positive. **d** Corresponding composite of transport anomalies on individual density surfaces. **e, f** Composite results as in **d**, but with transport anomalies decomposed into contributions from area anomalies and velocity anomalies on each density surface, respectively. In all panels, green dashed line denotes the multimodel mean density of the maximum overturning streamfunction (σ_{moc}) in O-Full, and black dash-dotted line denotes the multimodel mean density of maximum low-frequency standard deviation (σ_{SDmax}) of the streamfunction in O-Full. Shaded regions indicate ±1 standard deviation across models, with colors corresponding to the associated mean lines.

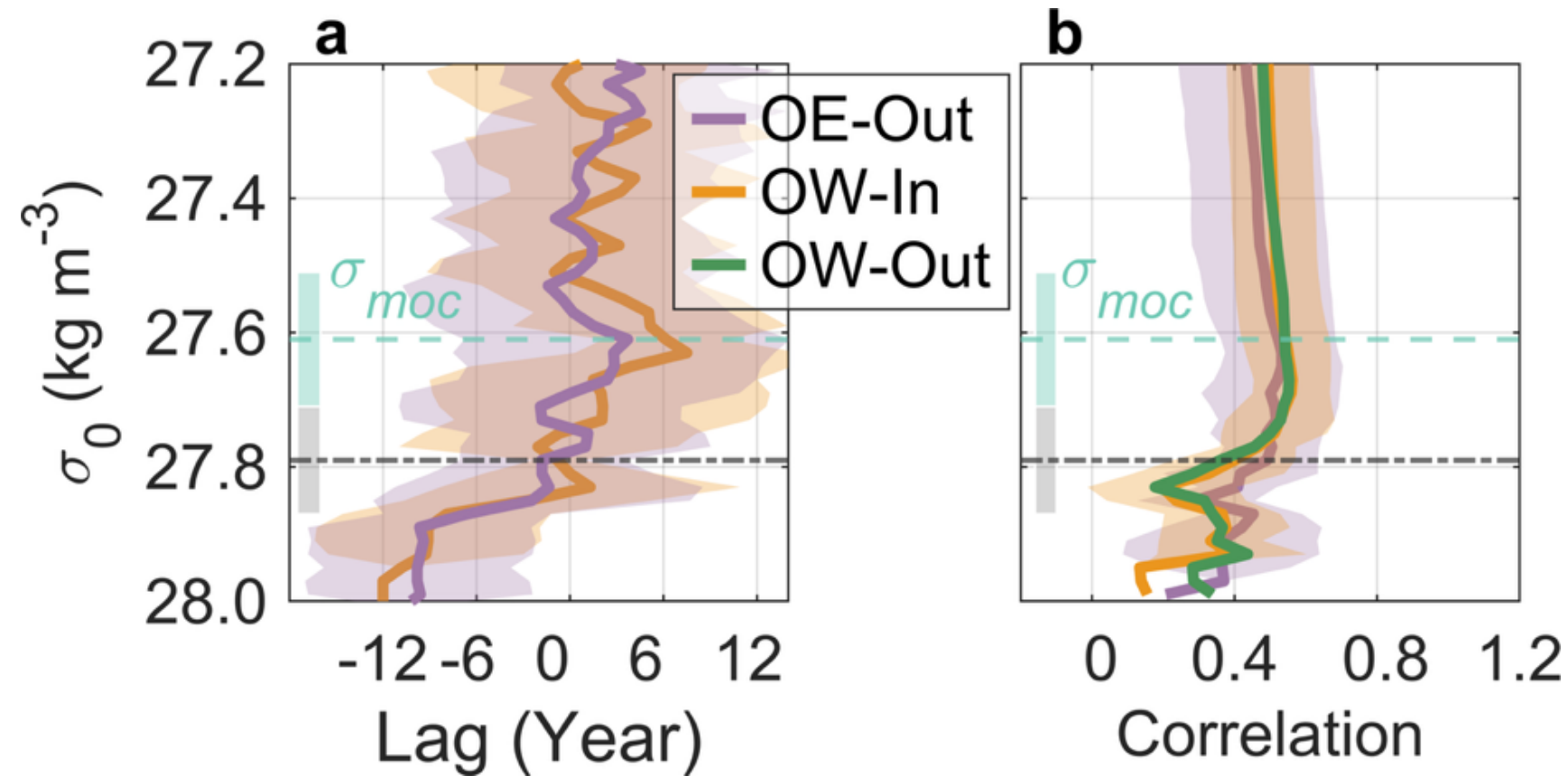

Supplementary Figure 7. Lead-lag relationships associated with transport variability along subpolar pathway. a Same as Fig. 3b of the main text, but showing the lag times at which the maximum correlation occurs between density-bin transport anomalies at σ_{SDmax} in OW-Out and density-bin transport anomalies at each density bin for OE-Out and OW-In. Negative (positive) lag values indicate that subpolar transport anomalies lead (lag) the downstream MOC. **b** Correlations between integrated transport anomalies for the three components and the OSNAP Full MOC. Solid lines denote the multimodel mean and shading indicates ±1 standard deviation across models.

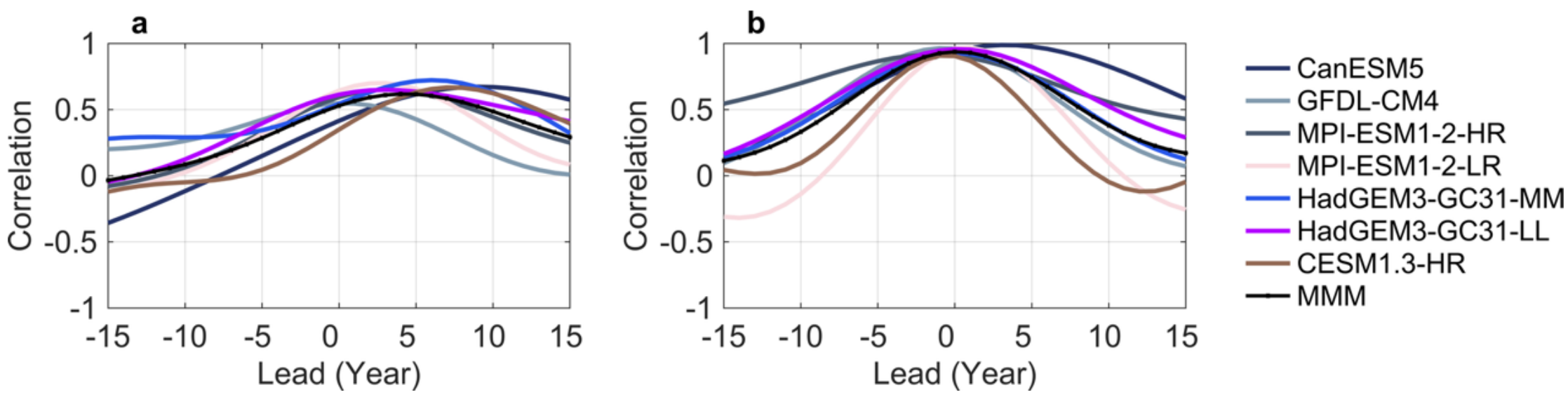

Supplementary Figure 8. Lead-lag relationships for cumulative dense transport anomalies in O-West. a Lead-lag correlation between OW-Out transport anomalies, cumulatively integrated from the bottom to the branch-specific σ_{SDmax}, and wintertime (December–February mean) surface-forced water mass transformation (SFWMT) across σ_{SDmax} in the Labrador Sea. Positive (negative) lag indicates that SFWMT leads (lags) OW-Out variability. **b** Lead-lag correlation between cumulative OW-Out and OW-In transport anomalies, each integrated from the bottom to its own branch-specific σ_{SDmax}. Positive (negative) lag indicates that OW-In leads (lags) OW-Out. Colored lines show individual models and the black line the multimodel mean (MMM). This Supplementary Figure includes only seven models because of data availability.

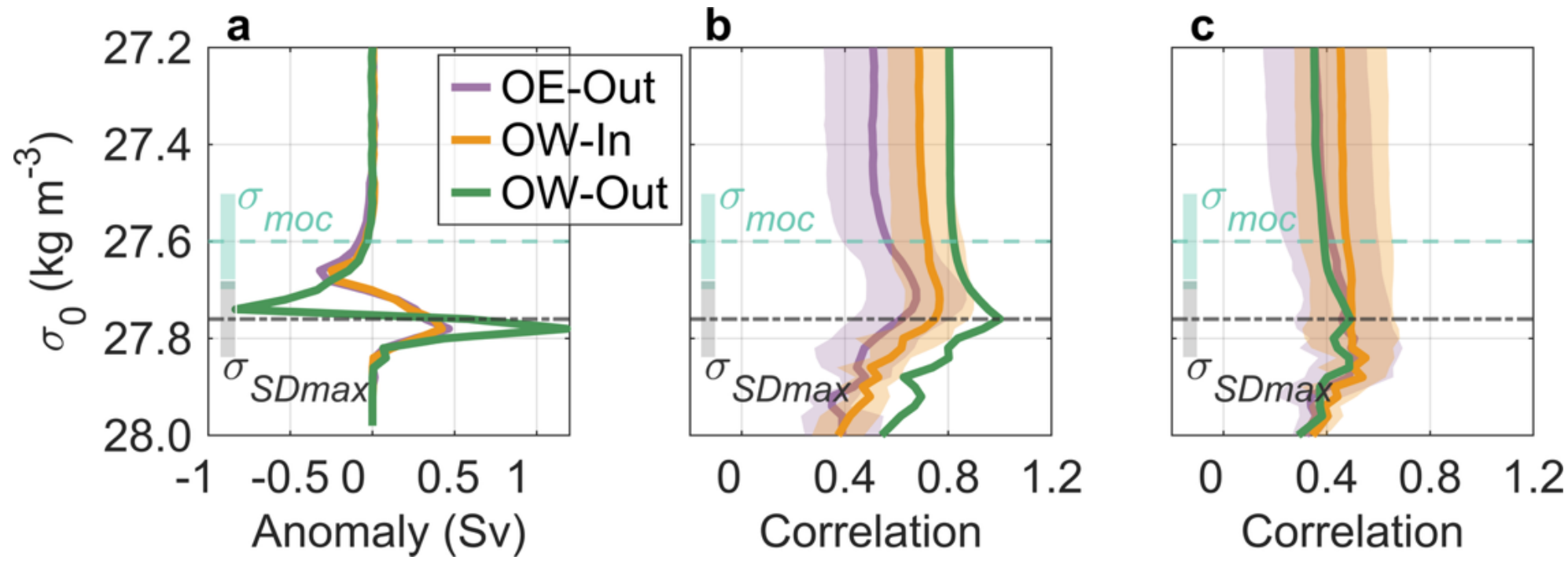

Supplementary Figure 9. Same as Fig. 3 of the main text, but for boundary-current transports. OE-Out, OW-In and OW-Out are calculated by integrating flow within 200 km offshore from the 500-m isobath, rather than over the full eastern or western basin. **a** Composite mean of transport anomalies on individual density surfaces for OE-Out, OW-In and OW-Out. **b** Correlation between integrated transport anomalies for OE-Out, OW-In and OW-Out and the integrated transport anomaly at σ_{SDmax} in OW-Out. **c** As in **b**, but showing correlations between integrated transport anomalies for the three components with the OSNAP Full MOC. The black dash–dotted line denotes the multimodel mean density of maximum low-frequency standard deviation (σ_{SDmax}) of the streamfunction in OW-Out.

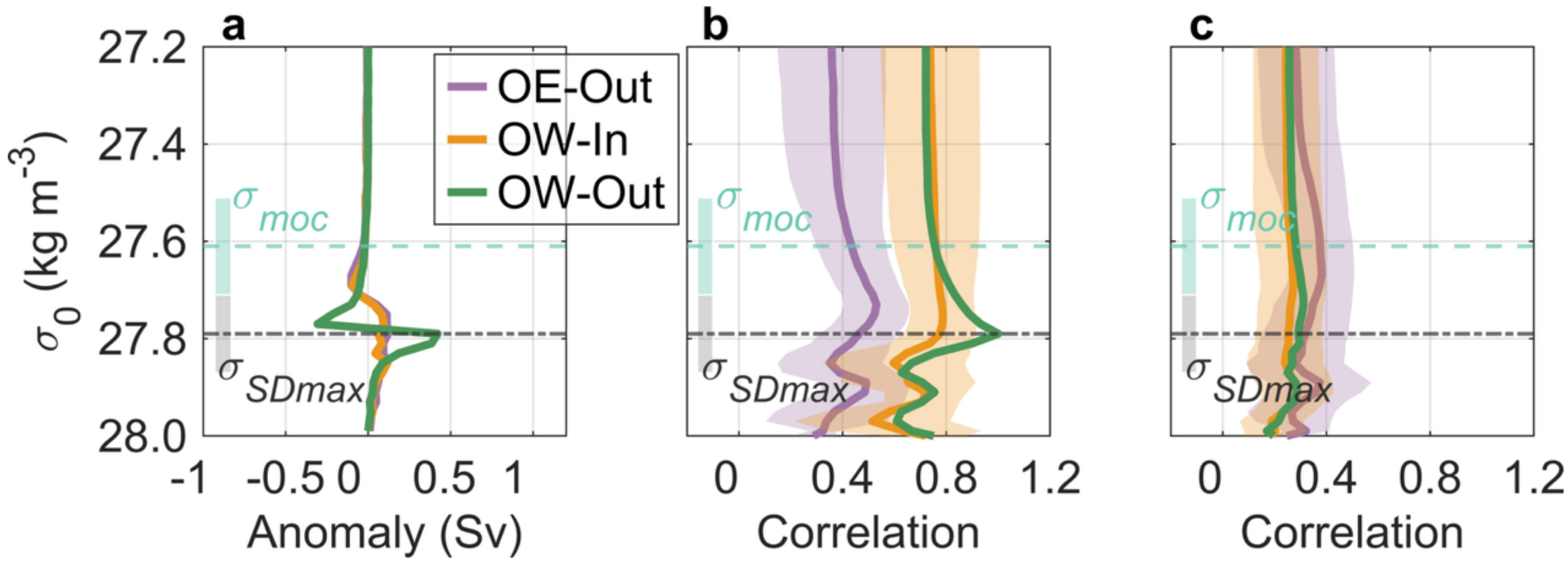

Supplementary Figure 10. Same as Fig. 3 of the main text, but for high-frequency (HF; interannual) variability. a Composite mean of transport anomalies on individual density surfaces for OE-Out, OW-In and OW-Out, constructed by averaging anomalies during periods of positive integrated transport anomaly at σ_{SDmax} in each component. **b** Correlation between integrated transport anomalies for OE-Out, OW-In and OW-Out and the integrated transport anomaly at σ_{SDmax} in OW-Out. **c** As in **b**, but showing correlations between integrated transport anomalies for the three components with the OSNAP Full MOC. The black dash–dotted line denotes the multimodel mean density of maximum high-frequency standard deviation (σ_{SDmax}) of the streamfunction in OW-Out.

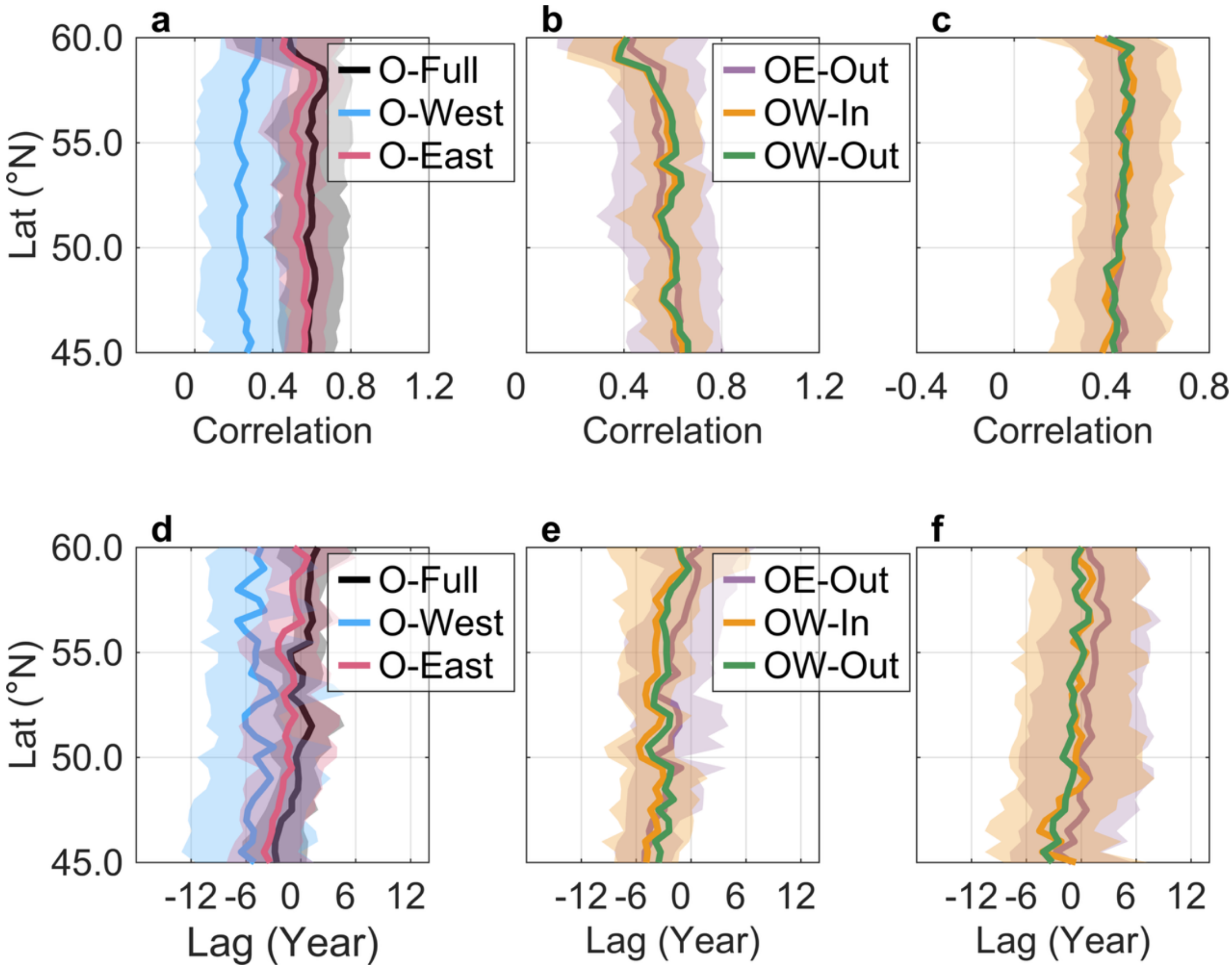

Supplementary Figure 11. Relationship between subpolar transport variability and downstream MOC and associated lag times. a Correlation between the overturning streamfunction across the OSNAP sections, evaluated over the density range 27.63-27.73 kg m^{-3}, and the downstream MOC at different latitudes. **b** As in **a**, but with the OSNAP overturning streamfunction decomposed into its constituent components (OE-Out, OW-In, and OW-Out). **c** Correlation between the downstream MOC and transport anomalies evaluated on individual density surfaces (density-bin transports) for the same components shown in **b**. **d-f** As in **a-c**, but showing the corresponding lag times. Negative values indicate that variability in the OSNAP component leads the downstream MOC.

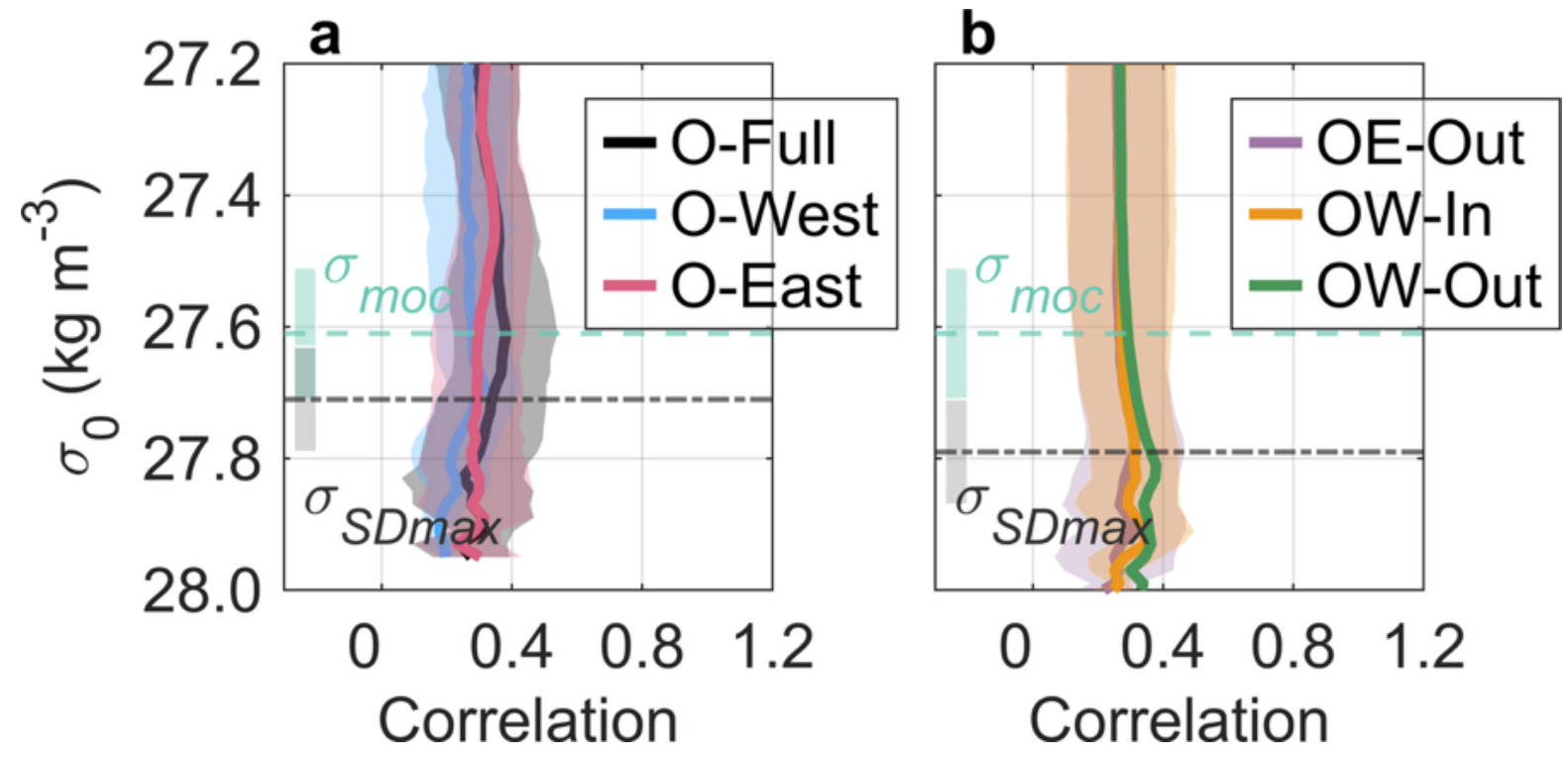

Supplementary Figure 12. Same as Fig. 4 of the main text, but for high-frequency (HF; interannual) variability. **a** Correlation between the overturning streamfunction at each density across the OSNAP sections and the downstream MOC at 50°N. **b** Same as **a**, but with the OSNAP overturning streamfunction decomposed into its constituent components (OE-Out, OW-In, and OW-Out). The black dashed line indicates the multimodel-mean density of maximum high-frequency standard deviation of the overturning streamfunction at OW-Out.

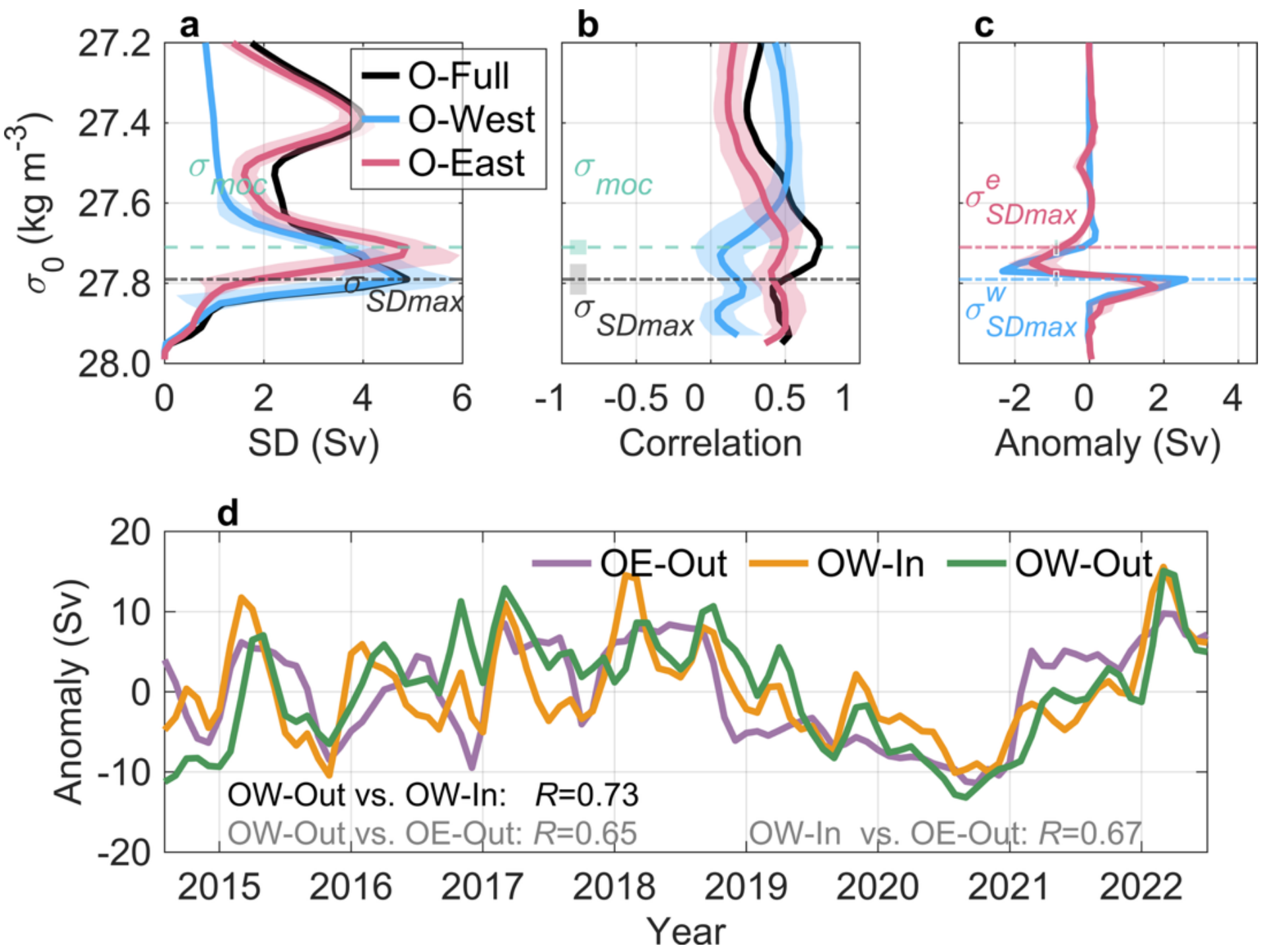

Supplementary Figure 13. Transport variability from CESM2 simulation. **a** Monthly standard deviation (SD) of transport as a function of density across OSNAP sections. **b** Correlation between overturning streamfunction at each density and the OSNAP-Full section MOC (O-Full). **c** Composite mean of transport anomalies on individual density surfaces, constructed by averaging monthly anomalies during periods of positive integrated transport anomaly at σ_{SDmax} in O-East (red) or in O-West (blue). The green dashed line denotes σ_{moc} and the black dash–dotted line denotes σ_{SDmax}, both estimated from OSNAP (Methods). **d** Time series of transport anomalies near σ_{SDmax} for southward transport across O-East (OE-Out), northward transport into O-West (OW-In) and southward transport exiting O-West (OW-Out). Transport anomalies are computed by integrating directional transport over the two density bins immediately below σ_{SDmax}. For visualization, the sign of OW-In is reversed to account for its opposite flow direction. Correlation coefficients among the three components are indicated at the bottom of the panel. To match the 96-month OSNAP record, we randomly select 50 independent segments of 96 consecutive months from the 300-year CESM2 simulation. Statistics shown here are summarized across these segments. Shaded regions indicate ±1 standard deviation across segments around the corresponding segment-mean curves.

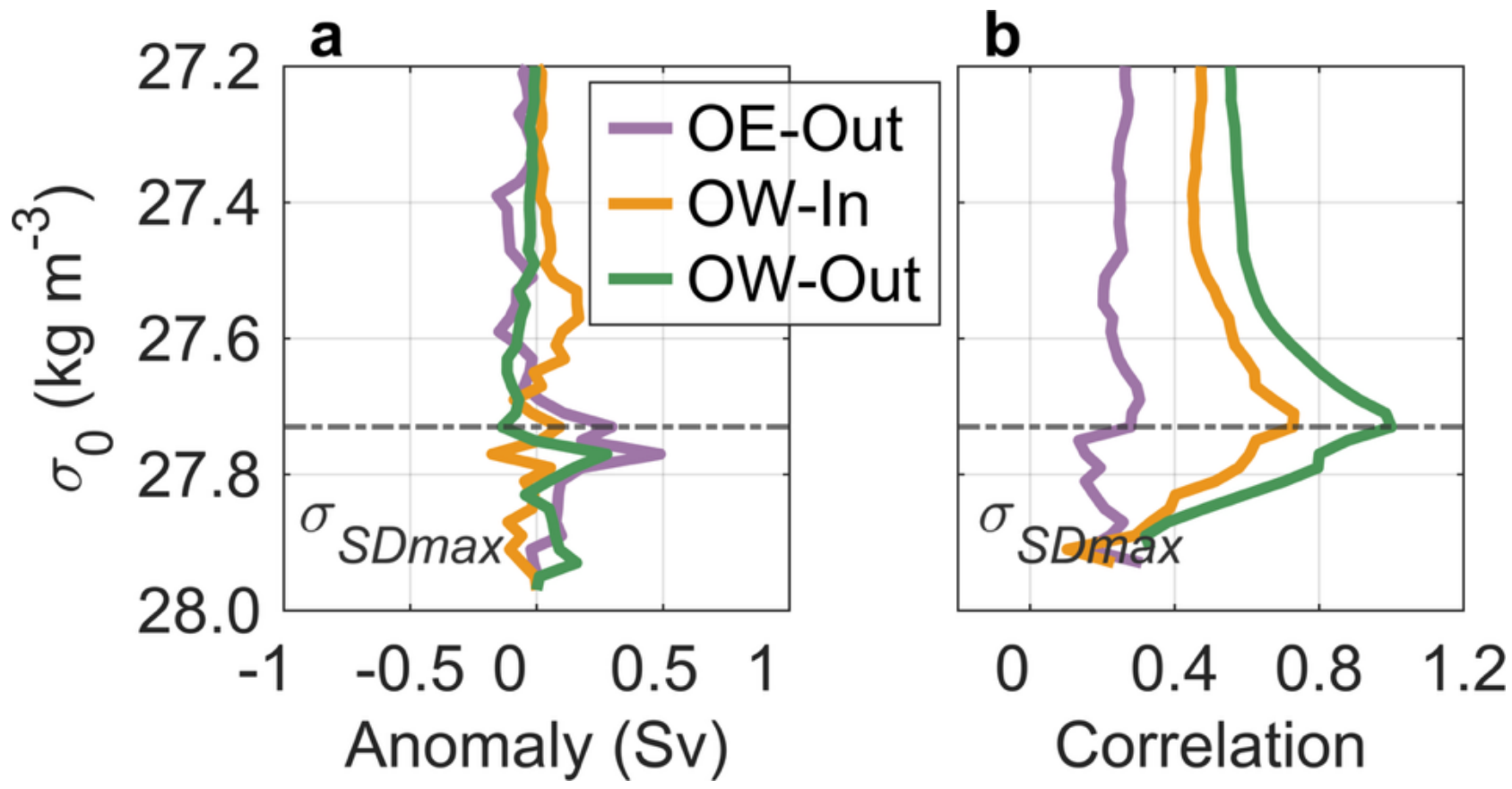

Supplementary Figure 14. Transport variability from OSNAP observations. **a** Composite mean of density-bin transport anomalies as a function of density for southward transport across O-East (OE-Out), northward transport into O-West (OW-In) and southward transport exiting O-West (OW-Out), constructed by averaging anomalies during periods of positive integrated transport anomaly at σ_{SDmax} in each component. **b** Correlation between integrated transport anomalies at each density for OE-Out, OW-In and OW-Out and the integrated transport anomaly at σ_{SDmax} in OW-Out. The green dashed line denotes σ_{moc} and the black dash-dotted line denotes σ_{SDmax}. All observational diagnostics are based on the August 2014–July 2022 OSNAP record.

Supplementary Table 1. Climate models used in this study. Most models are CMIP6 models, except CESM1.3-HR. The table lists nominal horizontal resolution in °latitude × °longitude, number of vertical levels and reference for each model. For all CMIP6 models, overturning streamfunction in density space is calculated using thetao, so, uo, and vo from the variant label r1i1p1f1.

	Model name	Horizontal	Vertical	Ocean module
1	ACCESS-ESM1-5 (*1*)	1 × 1	z^* 50	MOM5
2	CanESM5 (*2*)	1 × 1	z 45	NEMO3.4.1
3	UKESM1-0-LL (*3*)	1 × 1	z^* 75	NEMO-HadGEM3-GO6.0
4	GFDL-CM4 (*4*)	0.25 × 0.25	$\rho - z^*$ 75	MOM6
5	CESM2 (*5*)	1 × 1	z 60	POP2
6	CESM1.3-HR (*6*)	0.1 × 0.1	z 60	POP2
7	MPI-ESM1-2-HR (*7*)	0.4 × 0.4	z 40	MPIOM1.6.3
8	MPI-ESM1-2-LR (*8*)	1.5 × 1.5	z 40	MPIOM1.6.3
9	HadGEM3-GC31-MM (*9*)	0.25 × 0.25	z^* 75	NEMO-HadGEM3-GO6.0
10	HadGEM3-GC31-LL (*10*)	1 × 1	z^* 75	NEMO-HadGEM3-GO6.0

References

1. Ziehn, T., M. A. Chamberlain, R. M. Law, A. Lenton, R. W. Bodman, M. Dix, L. Stevens, Y.-P. Wang, J. Srbinovsky, The Australian Earth System Model: ACCESS-ESM1.5. *J. Southern Hemisphere Earth Syst. Sci.* **70**, 193–214 (2020).

2. Swart, N. C., J. Cole, V. Kharin, M. Lazare, J. Scinocca, N. Gillett, J. Anstey, V. Arora, J. Christian, S. Hanna, Y. Jiao, W. Lee, F. Majaess, O. Saenko, C. Seiler, C. Seinen, A. Shao, L. Solheim, K. von Salzen, D. Yang, B. Winter, The Canadian Earth System Model version 5 (CanESM5.0.3). *Geosci. Model Dev.* **5**, 1–68 (2019).

3. Sellar, A. A., C. G. Jones, J. P. Mulcahy, Y. Tang, A. Yool, A. Wiltshire, F. M. O'Connor, M. Stringer, R. Hill, J. Palmieri, S. Woodward, L. de Mora, T. Kuhlbrodt, S. T. Rumbold, D. I. Kelley, R. Ellis, C. E. Johnson, J. Walton, N. L. Abraham, M. B. Andrews, T. Andrews, A. T. Archibald, S. Berthou, E. Burke, E. Blockley, K. Carslaw, M. Dalvi, J. Edwards, G. A. Folberth, N. Gedney, P. T. Griffiths, A. B. Harper, M. A. Hendry, A. J. Hewitt, B. Johnson, A. Jones, C. D. Jones, J. Keeble, S. Liddicoat, O. Morgenstern, R. J. Parker, V. Predoi, E. Robertson, A. Siahaan, R. S. Smith, R. Swaminathan, M. T. Woodhouse, G. Zeng, M. Zerroukat, UKESM1: Description and Evaluation of the U.K. Earth System Model. *J. Adv. Model Earth Syst.* **11**, 4513–4558 (2019).

4. Held, I. M., H. Guo, A. Adcroft, J. P. Dunne, L. W. Horowitz, J. Krasting, E. Shevliakova, M. Winton, M. Zhao, M. Bushuk, A. T. Wittenberg, B. Wyman, B. Xiang, R. Zhang, W. Anderson, V. Balaji, L. Donner, K. Dunne, J. Durachta, P. P. G. Gauthier, P. Ginoux, J. C. Golaz, S. M. Griffies, R. Hallberg, L. Harris, M. Harrison, W. Hurlin, J. John, P. Lin, S. J. Lin, S. Malyshev, R. Menzel, P. C. D. Milly, Y. Ming, V. Naik, D. Paynter, F. Paulot, V. Rammaswamy, B. Reichl, T. Robinson, A. Rosati, C. Seman, L. G. Silvers, S. Underwood, N. Zadeh, Structure and Performance of GFDL's CM4.0 Climate Model. *J. Adv. Model Earth Syst.* **11**, 3691–3727 (2019).

5. Danabasoglu, G., J. F. Lamarque, J. Bacmeister, D. A. Bailey, A. K. DuVivier, J. Edwards, L. K. Emmons, et al. The Community Earth System Model Version 2 (CESM2). *J. Adv. Model Earth Syst.* **12**, e2019MS001916 (2020).

6. Zhang, S., H. Fu, L. Wu, Y. Li, H. Wang, Y. Zeng, X. Duan, W. Wan, L. Wang, Y. Zhuang, H. Meng, K. Xu, P. Xu, L. Gan, Z. Liu, S. Wu, Y. Chen, H. Yu, S. Shi, L. Wang, S. Xu, W. Xue, W. Liu, Q. Guo, J. Zhang, G. Zhu, Y. Tu, J. Edwards, A. Baker, J. Yong, M. Yuan, Y. Yu, Q. Zhang, Z. Liu, M. Li, D. Jia, G. Yang, Z. Wei, J. Pan, P. Chang, G. Danabasoglu, S. Yeager, N. Rosenbloom, Y. Guo, Optimizing high-resolution Community Earth System Model on a heterogeneous many-core supercomputing platform. *Geosci. Model. Dev.* **13**, 4809–4829 (2020).

7. Müller, W. A., J. H. Jungclaus, T. Mauritsen, J. Baehr, M. Bittner, R. Budich, et al. A Higher-resolution Version of the Max Planck Institute Earth System Model (MPI-ESM1.2-HR). *J. Adv. Model Earth Syst.* **10**, 1383–1413 (2018).

8. Mauritsen, T., J. Bader, T. Becker, J. Behrens, M. Bittner, R. Brokopf, V. Brovkin, M. Claussen, T. Crueger, M. Esch, I. Fast, S. Fiedler, D. Fläschner, V. Gayler, M. Giorgetta, D. S. Goll, H. Haak, S. Hagemann, C. Hedemann, C. Hohenegger, T. Ilyina, T. Jahns, D. Jimenéz-de-la-Cuesta, J. Jungclaus, T. Kleinen, S. Kloster, D. Kracher, S. Kinne, D. Kleberg, G. Lasslop, L. Kornblueh, J. Marotzke, D. Matei, K. Meraner, U. Mikolajewicz, K. Modali, B. Möbis, W. A. Müller, J. E. M. S. Nabel, C. C. W. Nam, D. Notz, S. S. Nyawira, H. Paulsen, K. Peters, R. Pincus, H. Pohlmann, J. Pongratz, M. Popp, T. J.

Raddatz, S. Rast, R. Redler, C. H. Reick, T. Rohrschneider, V. Schemann, H. Schmidt, R. Schnur, U. Schulzweida, K. D. Six, L. Stein, I. Stemmler, B. Stevens, J. S. von Storch, F. Tian, A. Voigt, P. Vrese, K. H. Wieners, S. Wilkenskjeld, A. Winkler, E. Roeckner, Developments in the MPI-M Earth System Model version 1.2 (MPI-ESM1.2) and Its Response to Increasing CO2. *J. Adv. Model Earth Syst.* **11**, 998–1038 (2019).

9. Menary, M. B., T. Kuhlbrodt, J. Ridley, M. B. Andrews, O. B. Dimdore-Miles, J. Deshayes, R. Eade, L. Gray, S. Ineson, J. Mignot, C. D. Roberts, J. Robson, R. A. Wood, P. Xavier, Preindustrial Control Simulations With HadGEM3-GC3.1 for CMIP6. *J. Adv. Model Earth Syst.* **10**, 3049–3075 (2018).

10. Williams, K. D., D. Copsey, E. W. Blockley, A. Bodas-Salcedo, D. Calvert, R. Comer, P. Davis, T. Graham, H. T. Hewitt, R. Hill, P. Hyder, S. Ineson, T. C. Johns, A. B. Keen, R. W. Lee, A. Megann, S. F. Milton, J. G. L. Rae, M. J. Roberts, A. A. Scaife, R. Schiemann, D. Storkey, L. Thorpe, I. G. Watterson, D. N. Walters, A. West, R. A. Wood, T. Woollings, P. K. Xavier, The Met Office Global Coupled Model 3.0 and 3.1 (GC3.0 and GC3.1) Configurations. *J. Adv. Model Earth Syst.* **10**, 357–380 (2018).
