# Supplementary material for: The Labrador Sea as a conduit for AMOC lower-limb transport anomalies on multiple timescales
Source: Nat Commun. 2026 Aug 28;17:10298. doi: 10.1038/s41467-026-77271-z (PMC13620149; doi:10.1038/s41467-026-77271-z)
Supplement: Supplementary file 2 [file 41467_2026_77271_MOESM2_ESM.pdf]

nature portfolio

Peer Review File

The Labrador Sea as a conduit for AMOC lower-limb transport anomalies on multiple timescales

Corresponding Author: Dr Jiajia chen

This file contains all reviewer reports in order by version, followed by all author rebuttals in order by version.

Attachments originally included by the reviewers as part of their assessment can be found at the end of this file.

Version 0:

Reviewer comments:

Reviewer #1

(Remarks to the Author)
See comments in the attached PDF.

Reviewer #2

(Remarks to the Author)
Review of "The Labrador Sea as a conduit of AMOC variability on multiple time scales" by Chen and Lozier

The authors examine AMOC transport and variability in the subpolar latitudes using long simulations from 9 CMIP6 models and make comparisons on short timescales with OSNAP observations. They find that subpolar AMOC variability is predominantly driven from the Irminger and Iceland basins, with contributions from the Labrador Sea being smaller and generally providing a role as an amplifier of variability which originates upstream. The text and figures are presented to a high standard, although some recent and relevant literature seems to be missing (e.g. Megann et al., 2021, Jackson and Petit, 2023 Petit et al., 2025). However, as it is currently presented the manuscript does not contain sufficient new knowledge. It needs to more clearly define the research question(s) it seeks to address and hence the conclusions which can be drawn from the subsequent analysis.

Major comments:
Overall, the analysis focuses on correlations of time series at σmoc (maximum overturning at a time varying density) and $SDmax$ (overturning at a time invariant density located the maximum standard deviation for each section), yet the manuscript does not make it clear to readers why we should expect a relationship to exist between the monthly transports at $\sigma SDmax$ and the net northward transport of the upper limb (MOC, as defined in this study) across the OSNAP sections. In failing to do so, the message of the paper is muddled and it is not clear what novel conclusions this paper delivers. The main conclusion, that variability on longer timescales is dominated by the Irminger and Iceland basins and not the Labrador Sea is not new (see e.g. Megann et al., 2021), and several previous studies have reported that the depth of maximum variability is found below the depth of the maximum transport at all latitudes (e.g. Hirschi et al., 2020 and references therein). I don't believe there is a rigorous explanation as to why this is the case in published literature, but I wonder if you are close with this work, noting that the deep convection in the Labrador Sea introduces additional variability to water masses that are already below the density of the maximum AMOC.

On Line 291-292: "we find strong variability at $\sigma SDmax$ has little influence on the overall MOC variability on both interannual and multidecadal timescales". This appears to be at odds with Yeager et al., (2021) - whose numerical simulation is included in the model ensemble used in this study – who found that "...low-frequency variability of density-space overturning at 45°N, which accounts for almost all the low-frequency variance in ocean heat transport into the SPNA, is driven by large amplitude fluctuations in the density distribution of southward-flowing lower limb transport."

Importantly, Yeager et al. (2021) demonstrate this relationship through a lagged composite analysis of the diapycnal

overturning and Labrador Sea surface water mass transformation rather than correlating the diapycnal overturning strength at 45° N with that taking place at $\sigma SDmax$ across OSNAP West. This leads me to question whether a similar relationship might be uncovered if a lagged correlation / composite analysis were used in this study. The physical mechanism proposed by Yeager et al. (2021) results in a time delay between the lower and upper limb anomalies of roughly a decade at ~55° N.

Several approaches could be taken to improve the clarity of the manuscript's central message. Firstly, the authors could broaden their analysis to consider which regions of density-space are most important for capturing MOC (or better still meridional heat transport variability, since this is what is climatically important). This would be a refocusing of the current analysis, which asks are transports at $\sigma SDmax$ related to the MOC strength, to instead address which regions of the diapycnal overturning stream function might best capture the variability at the isopycnal of maximum diapycnal overturning. Alternatively, the authors could explore whether transport variability at $\sigma SDmax$ is able to capture downstream MOC variability (as found by Yeager et al., 2021) despite being poorly correlated with local, subpolar diapycnal overturning variability. This might suggest that the communities' strong focus on the variability found at the isopycnal of maximum overturning at OSNAP is not necessarily where the subpolar overturning exhibits its strongest relationship to downstream (say 45° N) MOC variability. Reshaping the manuscript with this focus would allow the authors to address whether the relative stability of the subpolar MOC, which is briefly discussed on Lines 306-309, is a further artifact of using the stream function maxima (since this conceals variability in the composition of the upper limb).

Minor comments:
The first section of the results opens with two paragraphs introducing and describing the OSNAP observations, but then focuses solely on analysis of the 9 CMIP6 models between lines 78 and 244. This transition is confusing at first, and there is scope to reorganise the section to improve readability.

Line 71: "The nine CMIP6 models were selected for their relatively low biases in bottom density" – It is worth expanding further, both on how the models were selected and on the limitations of CMIP-class models. I can't see how the 9 models were objectively identified from Heuze, 2021 on the basis stated. Looking at their Fig. 3, which shows bottom density biases and RMSE, why, for example, was CanESM5 selected over the EC-Earth, MRI-ESM2-0 or CNRM models? CMIP-class models are in general not capable of representing key processes like the dense overflows from the Nordic Seas (CESM2's overflow parameterisation excepting) and often have unrealistically vigorous deep convection in the Labrador Sea, so they are unlikely to exhibit low biases in bottom density for the right reasons.

Line 200: It is unclear in this paragraph whether the time series are the total northward (southward) transport across the section (Lines 200-203). Figure 4a shows anomalies. How do you calculate the anomalies (removal of initial value, mean, line of best fit, …?)?

Related, flipping the sign of OW-In would make visual comparison (e.g. in Fig 4A, D) with the other two lines and correlation values much easier. The labelling of the curves implies positive into the Labrador Sea for "OW-In" and positive out of the Labrador Sea for "OW-out". It is not surprising that OW-In and OW-out are so highly correlated with each other, since the Labrador Sea can be regarded as almost a closed basin. There can only be minimal change in storage so the difference between inflow and outflow will be dominated by flows through the Hudson and Davis Straits. Therefore, the interesting aspect is not correlation between the inflow and outflow, but the differences in the water mass properties of those two flows.

Line 263: suggest "…also offset each other on monthly timescales."

Line 263-4: Please clarify this statement. Don't the high (and presumably significant?) correlations between OW-In, OW-Out and OE-Out, and the fact that variability is largest below σmoc, support the argument that a single boundary may be sufficient to capture the overall MOC strength?

Line 292-94: "Specifically, the multi-model mean correlation coefficients..." Please clarify whether the correlation coefficients are calculated on the multi-model mean, or whether this is the ensemble mean of the correlation coefficients computed on each of the models separately.

Typos:
L142: "raging" > "ranging"
L167: Missing "The" > "The large black circle…"

References:
Hirschi, J. J.-M., Barnier, B., Böning, C., Biastoch, A., Blaker, A. T., Coward, A., et al. (2020). The Atlantic meridional overturning circulation in high-resolution models. Journal of Geophysical Research: Oceans, 125, e2019JC015522. https://doi.org/10.1029/2019JC015522

Jackson, L.C., Petit, T. North Atlantic overturning and water mass transformation in CMIP6 models. Clim Dyn 60, 2871–2891 (2023). https://doi.org/10.1007/s00382-022-06448-1

Megann, A., Blaker, A., Josey, S., New, A., & Sinha, B. (2021). Mechanisms for late 20th and early 21st century decadal

AMOC variability. Journal of Geophysical Research: Oceans, 126, e2021JC017865. https://doi.org/10.1029/2021JC017865

Petit, T., Robson, J., Ferreira, D., Yeager, S., & Evans, D. G. (2025). Coherence of the AMOC over the subpolar North Atlantic on interannual to multiannual time scales. Geophysical Research Letters, 52, e2025GL115171. https://doi.org/10.1029/2025GL115171

Reviewer #3

(Remarks to the Author)
I co-reviewed this manuscript with one of the reviewers who provided the listed reports. This is part of the Nature Communications initiative to facilitate training in peer review and to provide appropriate recognition for Early Career Researchers who co-review manuscripts.

Reviewer #4

(Remarks to the Author)
The manuscript titled "The Labrador Sea as a conduit of AMOC variability on multiple time scales" by Chen and Lozier uses climate simulations to look at variability of the Atlantic meridional overturning circulation (AMOC) in the subpolar North Atlantic (SPNA), calculated both at σ(MOC) the maximum overturning density, and σ(SDmax), the density with the highest standard deviation. The aim of the study is to reconcile previous observational results that show the eastern SPNA dominates overturning variability with model studies that had suggested an important role of the Labrador Sea in the western SPNA in setting longer-term variability.
The authors find that σ(MOC) transport is dominated by O-East even on longer time scales, and although the peak standard deviation of the overturning stream function is set by variability in the O-West section, this largely results from transport changes within the lower AMOC branch, with little effect on overall strength of the AMOC.

This study is a valuable addition to the recent literature discussing the role of water mass transformation and overturning in the eastern and western SPNA, and represents an important step in understanding what drives multi-decadal AMOC variability. While I think the results are robust, I do feel some improvements need to be made in how they are presented, and the methods and results should be clarified by providing more information about the calculations and improving the phrasing of some paragraphs

General comments:

I think the methods and materials section should have a bit more detail about how the calculations were done, currently I find it difficult to fully follow how the main results were derived. In particular:
- How was the σ(SDmax) transport/transport anomaly actually calculated? I believe the "normal" OSNAP transport is calculated by integrating the equation in line 345 in density space from the lightest density to σ(MOC). So is the SDmax transport in e.g. Fig. 2A the integral from the surface to σ(SDmax)? Or just the transport in some density range around σ(SDmax)?
- Similarly, I couldn't find any mention of how the partitioning into southward and northward flow in Fig,. 4/5D is achieved; Is it summing together all the positive transport values at a given time step for OW-In and all the negative ones for OW/OE-Out? Is it a certain segment of the OSNAP line, e.g. OW-In is the eastern half of OSNAP-West and OW-Out is the western half?

For the discussion, the finding of "inherited" upstream variability in this study fits nicely with recent work on the continued transformation of surface into deep waters in the subpolar gyre (e.g. Buckely et al 2023, https://royalsocietypublishing.org/doi/full/10.1098/rsta.2022.0181; Tooth et al 2025, https://egusphere.copernicus.org/preprints/2025/egusphere-2025-1132/) and it would be good to discuss that connection a bit more in the paper.

Specific comments:

Line 30-33: This statement makes it sound like overflow waters are not part of NADW, which I don't think is true. I would say that NADW is composed of lighter waters formed by open ocean convection (LSW/uNADW) and denser overflow waters (DSOW & ISOW/lNADW)

Line 41-42: The statement could be strengthened by adding phraseing connecting the statements about Pickart et al (ref. 11) and Lozier et al (ref. 12). E.g. something like "Consistent with their findings, direct observations from OSNAP revealed…"

Line 56-58: I don't' have a specific suggestion here, but I think the motivating questions could be phrased a bit better

Line 60-65: Again, I think this paragraph could be reworded a bit to better set up the rest of the study. For example, there's no need to start with "Motivated by these questions" after the previous paragraph talked about "questions that motivate our study". Just saying "To address these questions…" would sound better

Line 102 (Fig. 1): I think this is a nice figure, but could be further improved with some changes:
- I can infer from the text that the figure shows CESM2 model data, but it would be good to explicitly mention that in the

caption. It could also be useful to mention the model time frame (300 years?) to provide context for the LF and HF filtering
- I don't see any colorbar, or even a mention of what the colors show (salinity?), in panel A
- It could be confusing to some readers to have red/blue for northward/southward flow in panel A and using a slightly different shade of red and blue for O-West and O-east
- It could be helpful to add a line fo σ(SDmax) in panels C and E; but I also understand that it might make the figure too busy, so it's fine to leave it out

Line 135-136: Are the "lagged downstream responses" shown somewhere in the SI, or is this just an extra calculation you did that is not shown?

Line 157 (Fig. 2): Add σ(MOC) to legend in panel C to be consistent with panel A. Also panel B and D should either be expanded to show a range from -1 to 1, or the zero line should be highlighted, to make interpretation more intuitive

Line 167-168: Describe small black/gray circles too – presumably the correlation between O-Full and 50N for individual models

Line 171-172: The first two sentences could be combined and shortened

Line 184-185: "changes in local water mass transformation occurs" doesn't sound right – do you mean either ""changes in local water mass transformation" or "changes in where local water mass transformation occurs"?

Line 215: It would be good to provide some explanation here for why you chose to show GFDL data; is it just to show how things look different in a different model? Is it because the GFDL model is a unique case with most of the change apparently happening between OE-Out and OW-In, or is this representative of other models?
Also, is the implication here that GFDL has relatively little transformation both across OSNAP East and OSNAP-West, but a lot of transformation SE of Greenland?

Line 232 (Fig. 4):
- As for fig. 1, it could be useful to visually show σ(SDmax) somehow. Maybe a thin dashed line in the colors of each transport component in panels B and C?
- "Of" sounds odd in the phrases like "southward flow of O_East", "northward flow of O_West", could be replaced by something like "across" or "through"
- The colors in panel D are confusing since they are related to, but not quite the same as, panels A-C, so it would be good to add a legend. Alternatively you could add y tick labels on the right side of the axis to label each row of correlations, in which case you don't actually need to differentiate colors at all

Line 259-261: Currently panel 5 D is not mentioned in the text, so it should be referenced here

Line 265: What are the 71 months shown in figure S7? Is this just one particular 71-month period out of the 300 years? If so how was it chosen? It's fine not to state this in the main text but it should be mention in the S7 caption

Line 278: I'd prefer mentioning what this shows instead of just saying "As in Fig. 4A"; i.e. "Time series of transport anomalies at σ(SDmax) as in Fig. 4A". Also since this is using observational data it would be good to state what time period the 71 months correspond to

Line 287-289: Mention the value of the isopycnal in question

Line 310-325: As mentioned above, it would be good to put this paragraph into the context of some other studies like Buckley et al 2023 and Tooth et al 2025

Line 329-330: Here too you could mention previous studies; e.g. that the dominant role of O-East variability further udnerscores the findings from Lozier et al 2019

Line 322-323: "even at monthly timescales" – isn't it only at monthly time scales? Since the LF and HF variability as defined for the models isn't resolved at present by OSNAP

Line 333-334: Could be phrased better "the OSNAP time series limits our ability to understand AMOC variability on longer timescales" makes it sound like OSNAP measurements are actively detrimental to understanding AMOC variability, which would be an unfortunate finding. I think it's better to say that OSNAP alone is insufficient to understand AMOC variability on these timescales, or simply that they are not (yet) resolved by OSNAP

Line 350: Should specify what σ0 is, i.e. potential density referenced to the surface

Line 365: I think you should reference Table S1 again here, and it could also be useful to provide the names of all the models somewhere in this section.

Line 378: It would be good to add here, or somewhere else in the materials and methods, some more info about the OSNAP data used, including the time period covered in the current version. I also think it would be good to add a reference either for the data product or for an earlier OSNAP paper describing how it is calculated since there are quite a few steps between the

actual measurements and the gridded T/S/v product used here

Line 381-383: Is the 0.08 kg/m3 offset applied to the data shown in the study (or fig. S8)? If not, maybe it’s better to phrase it differently than “after aligning the profiles”, which sounds like an offset was applied to the data. Also, it’s unclear what the “average coefficient of 0.83” refers to

Line 387-389: Need some more context for “the modeled range of 71-month means”. Does this mean splitting the 300-year time series up into 71-month segments and calculating the mean for each? Is the range the lowest to highest value of those means? I’m also not sure what the “range of monthly SD” is, and in any case there are no values provided either for it or the observed value it’s compared to, so the statement isn’t too useful.
I think it could be easier to follow if you just state the mean and SD of the 71-month means, compared to the mean and uncertainty of the observed value

Line 388: No need to define Sverdrup again since you already did in line 353

Version 1:

Reviewer comments:

Reviewer #1

(Remarks to the Author)
This is a much improved version of the manuscript and it is generally well written. The paper makes an important point that variability in dense water transport is not necessarily linked to the downstream AMOC strength variability because compensation can occur in density space.

However, much of the current discussion remains largely descriptive. In my view, the more fundamental issue is understanding what controls the density of maximum AMOC variability (σ_{SDmax}) and why it differs from the density of maximum overturning (σ_{moc}). The manuscript repeatedly demonstrates that compensation occurs in density space, but this essentially restates the result that $\sigma_{\text{SDmax}} > \sigma_{\text{moc}}$, rather than explaining why this relationship arises dynamically.

In addition, the discussion of how the present results reconcile with Yeager et al. (2021) is not sufficiently convincing. I am also not persuaded by the authors' conclusion that the Labrador Sea acs only as a conduit for AMOC variability, particularly for density classes close to σ_{SDmax} (see specific comments below).

Specific Comments

1. **Interpretation of σ_{SDmax} vs. σ_{moc}**: While it is useful to show that variability in dense water transport does not necessarily translate into downstream AMOC strength variability, the more important question is what controls σ_{SDmax} and why it differs from σ_{moc}. Given a perturbation in the water mass transformation, compensation of transport variability across density classes is expected in density space. Therefore, the discussion of compensation largely reiterates the finding that $\sigma_{\text{SDmax}}>\sigma_{\text{moc}}$, rather than addressing the dynamical reason why this occurs. The manuscript would be strengthened by a clearer explanation of the processes that set these two density scales and why they differ.

2. **Source of deep water transport variability**: To examine the origin of dense transport variability, the authors plotted transport anomalies near σ_{SDmax} for three components in Figure 3a: southward transport exiting O-East, northward transport entering O-West, and southward transport exiting O-West. Based on the correlations among these three components, the authors suggest that the transport variability originates in OSNAP-E and is subsequently advected into the Labrador Sea, where further modifications occur.

My difficulty arises from comparing Figure 3a with Figure 1d,f. Figure 1d,f suggest that the transport variaiblity near σ_{SDmax} associated with O-East is negligible compared to that in O-West. This implies that the AMOC variability near σ_{SDmax} reflect water mass transformations within the Labrador Sea. It would be helpful to also include the northward transport entering O-East (OE-In) in Figure 3a. It is highly likely that substantial compensation also occur between OE-In and OE-Out. If such compensation exists, would this mean that the Irminger Sea is also a conduit for AMOC variability?

I agree that the downstream AMOC variability (in terms of its strength) is mainly associated with OSNAP-E, as shown in previous studies. However, the overturning variability near σ_{SDmax} may still originate from Labrador Sea water mass transformations (surface plus interior). If this interpretation is correct, the authors should be more precise about the density range when discussing the origin of AMOC variability.

3. **Relationship to Yeager et al. (2021)**: The discussion of how these results relate to Yeager et al. (2021) is currently ambiguous. Yeager et al. (2021) showed a connection between surface forced dLSW formation and AMOC strength variability on multi-decadal timescales, while this paper finds little correlation between AMOC strength and dense water transport.

As the authors noted in the introduction, the dense water transport variability is not necessarily the same as surface forced water mass formation. However, it remains unclear why the surface forced water mass formation correlates well with the AMOC in Yeager et al. (2021). This may be reconciled by combining the discussions in Yeager et al. (201) with the results shown in Figure 4b of this study.

Reviewer #2

(Remarks to the Author)
Please see the attached document: "Review of revised Chen and Lozier.pdf".

Reviewer #3

(Remarks to the Author)
I co-reviewed this manuscript with one of the reviewers who provided the listed reports. This is part of the Nature Communications initiative to facilitate training in peer review and to provide appropriate recognition for Early Career Researchers who co-review manuscripts.

Reviewer #4

(Remarks to the Author)
REVIEW OF "The Labrador Sea as a conduit of AMOC variability on multiple time scales", Chen et al 2026, revision 1

I think the authors did a great job responding to the comments by myself and other reviewers, and the revised manuscript is much clearer in its motivation, scope, and key results. I don't have any significant comments in terms of content at this point, but did notice some areas where the text could be changed to further improve the clarity and readability of the paper, as outlined below.

MINOR COMMENTS

L 44-45: "direct observations two decades later of the meridional overturning circulation via the Overturning in the Subpolar North Atlantic Program" - could improve flow of this sentence; maybe "two decades later" is not necessary

L 81: Should reference be earlier? e.g. "(CMIP6 (17); Supplementary Table 1)" instead of "(CMIP6; Supplementary Table 1) (17)"

L 86: It could be helpful for readers not familiar with OSNAP to say explicitly that O-West + O-East = O-full; for example, change the text to "across a full section (O-Full) *comprised of* two coast-to-coast subsections (O-West and O- East; Fig. 1a)."

L 100: “as with observations”: Again, this could be changed to improve clarity for some readers; as far as I can tell the paper doesn't actually include the OSNAP observational streamfunction anywhere either in the main text or supplementary, and while the authors and many readers will be intimately familiar with it, it could be helpful to include for those that aren't. Alternatively, at least a reference to an OSNAP paper that shows the streamfunction would be useful

L 102: “Looking downstream” - It could be useful to add a nominal latitude for OSNAP, which of course might be difficult since it spans a relatively broad range. But some indication on the figure could help put this in context; either with a symbol or line at a representative latitude, or showing the lat range covered by OSNAP

L 116-117: “We next confirm this indication” - I think it would be better to say something like "we further explore this connection" instead to avoid already giving a conclusion to results that are only shown in the next section

L 125/Fig 1a: I appreciate the additon of the colorbar but think a little more information is still needed: Is the potential density shown taken from observations or CESM2? And is it a time-mean over the whole period?

L 141: “we examine where in density space we might find a strong relationship.” - could be phrased slightly better

L 143/Fig 2a: Why doesn't the black line equal 1 at sigma_moc? Is it because for OSNAP MOC the density is time variable and here a constant density is used?

L 160/Fig 2: It would be good in the figure caption to note again that this is CESM2 data

L 165/Fig 2: add panels in the description of c and d to improve clarity; e.g. "decomposed into contributions from area anomalies (c) and velocity anomalies (d)"

L 207/Fig 3a: Reading this I was immediately wondering why the authors switched from showing the CESM2 model in fig 1/2 to MPI-ESM1-2-HR in fig 3. The reasoning is stated in the methods in L 555-557: "MPI-ESM1-2-HR is shown in Fig. 3a because the correlation between OW-Out and OW-In in this model is close to the multimodel mean, making it a

representative example of the ensemble behavior." It might be a good idea to add some explanation along those lines in the main text to avoid readers thinking the model may be "cherry-picked" for this figure

L 239/fig 3b: Could be helpful to add a legend to the panel, or a description in the text, since the colors are not strictly the same as panel a; i.e. purple line is OE-out/OW-out correlation, and orange line is OW-in/OW-out correlation,

L 285/Fig 4: Should this be "black dash-dotted line" rather than "black solid line"?

L 291-294/Fig 5: It could be useful to compare this shorter timescale variability to model data; in fact I believe the authors did so in the original manuscript (fig. S7 in original supplementary) but now took out that analysis. I think it would be valuable to see that models and observations show similar patterns on short timescales, supporting the use of the models for the longer-term analysis in Fig 3
Also, since panels b and c are similar to previous figures on the CESM2 data (fig 2a, b) it would be good to at least briefly relate these, even though the time scales may be different

L 368-369: I would remove the phrase “this is not unexpected”; just saying it's consistent with previous studies should be sufficient

L L427-433: Could improve flow of this paragraph by moving up the sentence about model velocity/property fields before the mention of GSW/potential density calculation, and also make it clearer initially that model data is also extracted along OSNAP, as the phrasing "while for the streamfunction across zonal sections (which we use with model output)" could be interpreted to mean ONLY zonal sections are used in models. You could clarify this by adding a similar clause at the beginning of the sentence "For the streamfunction across each OSNAP section (used both with models output and observations)"

L 481-493: It could be useful to have separate subsections for this area/velocity decomposition and the preceding OE-Out/OW-In/OW-Out decomposition. Maybe this could also be merged with the earlier introduction of V (444 - 452)?

L 495: It could be helpful to mention the density offset for each model e.g. in a table; alternatively the multimodel sig_SDmax could be marked in fig S1 so readers can visually see the offset

L 569/Table S1: For reproducibility it would help to add which CMIP6 variables were downloaded (T, S, v?) and which model variant is used for each model, i.e. identifiers with the format r1i1p1f1

Version 2:

Reviewer comments:

Reviewer #1

(Remarks to the Author)
See comments in attached PDF.

Reviewer #2

(Remarks to the Author)
I am happy that all the points I raised have been satisfactorily addressed.

Reviewer #3

(Remarks to the Author)
I co-reviewed this manuscript with one of the reviewers who provided the listed reports. This is part of the Nature Communications initiative to facilitate training in peer review and to provide appropriate recognition for Early Career Researchers who co-review manuscripts.

Reviewer #4

(Remarks to the Author)
I thank the authors for addressing my comments, and have no further suggestions at this time.

Version 3:

Reviewer comments:

Reviewer #1

(Remarks to the Author)
I appreciate the authors' clarifications. This is the fourth round of review, and my only remaining requirement for acceptance is resolving a contradiction between Figure 1 and Figure 4–this can be achieved by replacing Figure 1 with a model that

better aligns with the multi-model mean.

The paper's main conclusion is that the Labrador Sea acts as a passive conduit for anomalies originating in OSNAP-East. However, the choice of CESM for Figure 1 directly contradicts this narrative and the multi-model results in Figure 4.

By definition, OSNAP-Full = OSNAP-West + OSNAP-East. In Figure 1, the variance in OSNAP-West dominates the total budget. A straightforward mathematical derivation dictates that the component dominating the variance must yield the higher correlation with the total sum.
Therefore, the physics shown in Figure 1 for CESM2 demand that OSNAP-West is the primary driver of full AMOC variability. This completely contradicts Figure 4, where the ensemble mean shows OSNAP-East dominating the correlation.

Once Figure 1 is replaced to align with the text and Figure 4, the manuscript will be structurally sound and ready for acceptance.

Version 4:

Reviewer comments:

Reviewer #1

(Remarks to the Author)
See attached PDF.

Reviewer #5

(Remarks to the Author)
After reading the full manuscript and the supporting material in detail I find that the manuscript's central conclusions are supported by the analyses and that the remaining disagreement reflects a misunderstanding of Figure 1 by the reviewer. My brief remarks below further explain how I came to this conclusion.

The introduction poses a very specific unresolved question raised by earlier published papers – how does transport variability in the density classes of the lower limb of the AMOC relate to the variability of the AMOC itself. The manuscript carries out a clear and careful analysis of couple climate models at different timescales – Low Frequency meaning multi-decadal, and High Frequency meaning Interannual. This is important because it has been argued that the mechanism relating transport in the deep waters to the strength of the AMOC may differ at longer and shorter timescales. Ultimately they show the same signals in the HF timescales as the LF which is very interesting and possibly not what we might expect to see – but on both timescales the central conclusions of the paper is supported by the evidence shown by the analysis. They use OSNAP observations to test the conclusions from the model analysis and find that they are too support the findings.

I find that the authors take the reader through the steps of the analysis very well. The logic is straightforward, understandable and valid. The figures are clever – the reader needs to take the time to understand what they show by reading the text and studying the captions to understand the data and the colours, but on doing that they are startlingly clear in leading the reader to the same conclusion as the authors.

Turning to the remaining disagreement after a thorough reading of the paper, I conclude that the reviewer concern is centred on two minor misunderstandings by the reviewer, both of which are addressed in the rebuttal statements.

Firstly the reviewer asserts that the full OSNAP MOC is the sum of OSNAP W and OSNAP E, which is incorrect. This concept was explained in the first OSNAP papers (Lozier et al 2017, Lozier et al 2019) and has been re-explained in every OSNAP paper since then.

The main concern is the second point however – the reviewer misinterpreted the high variability in the streamfunction at around 27.8 in OSNAP W as demonstrating the highest variability in the MOC is found in OSNAP W. This is not correct as is now explained in the version of manuscript that I am reading. It is a key point in the analysis that high variability in the overturning streamfunction in density space below the maximum of the streamfunction, ie in a class denser than the definition of the MOC, does not relate to the variability in the strength of the MOC itself. When one understands that distinction, the suitability of the model as an illustration of the analysis is no longer a cause for objection. The authors have added to text to ensure other readers find it easier to see what the figure is showing, and the additional figure in Supplementary Material helps allay any concerns that this chosen model might not be representative of the multi-model mean.

In conclusion I find that manuscript's "Labrador Sea as a conduit" conclusion is consistent with Figure 1.

Open Access This Peer Review File is licensed under a Creative Commons Attribution 4.0 International License, which permits use, sharing, adaptation, distribution and reproduction in any medium or format, as long as you give appropriate credit to the original author(s) and the source, provide a link to the Creative Commons license, and indicate if changes were made.
In cases where reviewers are anonymous, credit should be given to 'Anonymous Referee' and the source.
The images or other third party material in this Peer Review File are included in the article's Creative Commons license, unless indicated otherwise in a credit line to the material. If material is not included in the article's Creative Commons license and your intended use is not permitted by statutory regulation or exceeds the permitted use, you will need to obtain permission directly from the copyright holder.
To view a copy of this license, visit https://creativecommons.org/licenses/by/4.0/

Reviewer 1: This study examined the overturning circulation variability in the North Atlantic, focusing on connections between the OSNAP sections and the downstream AMOC. They found that (i) overturnings in the Irminger and Iceland basins, rather than the Labrador Sea, show great correlations with the AMOC. They also suggested that (ii) the Labrador sea overturning variability may be inherited from the upstream Irminger/Iceland basins. While I agree with their key point # (i), which is not surprising based on previous observational/modelling studies, I don't think their point #(ii) is correct (Or at least it is not supported by the current analysis; See major concern #3 below).

We thank the reviewer for this summary and for highlighting these two central points, both of which we address below.

Major Concerns

[1] This study is partly motivated by the discussion of Yeager et al. (2021), but the authors appear to misunderstand the key messages in that paper, which by no means speculated a better correlation between OSNAP-West and AMOC than OSNAP-East. Yeager et al. (2021) suggested that deep convections in the Labrador Sea precede the multidecadai variability of the AMOC by roughly a decade (their Figure 7) -- this is achieved by modifying the gyre circulation in the subpolar North Atlantic. It does not imply a more significant role of the Labrador Sea in driving the AMOC if this is quantified using correlation. If a similar analysis (Figure 7) were carried out in these coarse-resolution models, how does it compare with Yeager et al. (2021)?

We thank the reviewer for raising this point and agree that Yeager et al. (2021) should not be interpreted as implying a stronger correlation between OSNAP-West and the AMOC than between OSNAP-East and the AMOC. We have revised the manuscript to avoid such an interpretation. We now write in our Introduction:

"Shortly thereafter, an eddy-resolving modelling study (15) showed that low-frequency AMOC anomalies at 45°N are preceded by pronounced lower-limb transport anomalies, which are closely associated with enhanced water mass transformation (WMT) within the Labrador Sea Water (LSW) density range. On this basis, the authors argued that although the Labrador Sea contributes only weakly to the mean and monthly variability of the AMOC, it may play a disproportionately important role in driving low-frequency AMOC variability."

We view our analysis as complementary to Yeager et al. (2021). While Yeager et al. emphasized the influence of Labrador Sea water mass transformation on multidecadal AMOC variability through its impact on the subpolar gyre circulation, our study focuses on how transport variability expressed at different density classes and along different branches of the circulation projects onto the local and downstream AMOC. Across many models and for both high and low frequency cases, we find the same signal as Yeager et al. did in their model: transport variability in the lower limb exiting the Labrador Sea is maximum near the density threshold for water mass formation in that basin. Our two studies differ though in two regards: 1. In the Yeager et al. study, the strong variability at LSW densities is attributed to water-mass formation in that basin, while our multimodel study attributes that strong variability largely to upstream processes. 2. In our multimodel study, the strong variability exiting the Labrador Sea on densities below σ_{moc} does not uniquely provide information on the downstream AMOC. We elaborate on both of these points below and in the revised text.

[2] Based on previous observational/modelling studies (e.g., Lozier et al., 2019; Menary et al., 2020), it is not surprising that the highest correlation is between the AMOC and O-East (σ_{moc}), especially on multi-decadal timescales, given that most of the transformation occurs in the Iceland and Irminger Seas (Petit et al., 2020). The variability of overturning circulation streamfunction in the Labrador Sea, peaking at a higher density than σ_{moc}, primarily represents a redistribution of deep water transport between water masses denser than σ_{moc}. 1) Why should we expect a direct correlation between O-West (σ_{SDmax}) and the AMOC? The key message of Yeager et al. (2021) is that deep convections in the Labrador Sea have merits for predictions of the AMOC, with respect to its internal variability on multi-decadal timescales. 2) What do these in-phase correlations, discussed in this paper, tell us? 3) What is responsible for the large inter-model spread? 4) Can we trust all these models?

As for your first question: There is no *a priori* reason to expect a strong in-phase correlation between overturning variability in the Labrador Sea at σ_{SDmax} and the AMOC. However, given the pronounced variability observed at these densities in the models and in OSNAP observations, we were motivated to understand the source of this variability and whether it has any impact on the local and downstream AMOC.

As for your second question: the revised manuscript no longer relies on in-phase correlations. we examine correlations between the AMOC and transport variability at all density surfaces, allowing for lead–lag relationships. Results from our lead-lag analyses are discussed in lines 215-217, 255-268.

As for your last two questions: The large inter-model spread reflects well-known differences among CMIP-class models in stratification, deep convection intensity, and water-mass transformation in the subpolar North Atlantic. Rather than relying on any single model,

the conclusions in our paper are drawn from structural features—such as maximum variability below σ_{moc}, density-space compensation and coherent inflow–outflow variability in the Labrador Sea —that are robust across models, timescales, and observations.

[3] The authors suggested that the variability in the Labrador Sea was advected from the upstream Irminger/Iceland basins. This is true for input/output volume transport across O-West, but how does this relate to the overturning circulation variability? The transports (OW-In, Ow-Out, and OE-Out) are part of the subpolar gyre circulation -- it would be surprising if they were not correlated with each other. The overturning circulation across O-West, by definition, is the residual between OW-In and OW-Out, and must be balanced by water mass transformation or volume tendency terms (e.g., expansion/contraction of LSW) in the Labrador Sea. If no transformation occurs and the water mass volume does not evolve in the Labrador Sea, OW-In will be exactly balanced by OW-Out. Thus, the overturning circulation variability in the Labrador Sea or the compensation does not "reflect the influence of upstream signals". At least, this is not supported by the current analysis (Lines 199-231).

We agree that overturning variability across O-West is, by definition, the residual between OW-In and OW-Out and must be balanced by local water mass transformation or volume tendency within the Labrador Sea. We note that this is fully consistent with our interpretation throughout the manuscript. We did not and do not intend to interpret basin-integrated O-West overturning variability as a direct reflection of upstream influence. We have clarified this point in the revised text (see lines 207-227, 265-268). Furthermore, we agree that *if* our analysis were based on the *total* in/out transport integrated over the full water column (or over the entire lower limb) across O-West, a strong correlation between OW-In and OW-Out would simply reflect volume conservation within the gyre.

However, our analysis is not based on the vertically integrated transport of the full water column or the entire lower branch, but rather on transports evaluated at different density surfaces or integrated from the bottom to a given density. In this context, OW-In and OW-Out are not a priori correlated. Our results show that as water enters the Labrador Sea, the strong variability signal shifts to denser layers and is locally amplified (Fig. 3). This indicates that the variability is not only advected into the basin but modified and amplified locally through water mass transformation processes. Such cross-isopycnal strengthening is fully consistent with the physical expectation that inflowing waters become denser in the Labrador Sea.

Minor Comments

1. [Lines 68-70]: Why are these 9 models selected? How do these 9 models reproduce the results of OSNAP regarding the mean overturning circulation?

As for your first question: Though we used Heuzé et al. (2021) as a broad reference to avoid models with extreme bottom-density biases in the Greenland–Iceland–Norwegian Seas and subpolar gyre, we otherwise chose models as explained in Methods (lines 532-549):

"We analyze simulations from nine coupled climate models participating in CMIP6 together with one high-resolution coupled model (CESM1.3-HR), using 300 years from each preindustrial control simulation to isolate internally generated variability. The full list of models and their key characteristics are provided in Supplementary Table 1.

The resulting ensemble spans six distinct ocean model formulations, with two model pairs differing primarily in horizontal resolution, providing a range of subpolar circulation representations without targeting specific processes. The nine CMIP6 models were drawn from the available archive primarily based on data availability, including long preindustrial control simulations and the three-dimensional fields required for density-space diagnostics. We used Heuzé et al. (2021) as a broad reference to exclude models with extreme bottom-density biases in the Greenland–Iceland–Norwegian Seas and subpolar gyre, which could otherwise dominate density-space structure and hinder inter-model comparison. Beyond this exclusion of clear outliers, no selection was made based on overturning behavior and the final ensemble represents a quasi-random subset spanning a range of ocean model formulations and horizontal resolutions. Accordingly, our analysis does not rely on the fidelity of any individual model, but instead emphasizes relationships that are consistent across models, time scales, and independent observations."

As for your second question: We have now added the time-mean overturning streamfunction in density space for all nine CMIP6 models to the Supplementary Information (new Supplementary Fig. 1). These mean structures exhibit substantial inter-model spread, particularly in O-West, both in magnitude and in the density of maximum overturning. These differences are expected given known CMIP-class limitations but do not affect our conclusions, which focus on variability and structural relationships rather than mean-state accuracy.

2. [Figure 1]: There appears to be some compensation between O-West and O-East around 27.7 kg m^{-3} (the variability of O-Full is much smaller than O-West and O-East). Any suggestions on what processes are causing this?

We agree that partial compensation between O-East and O-West may contribute to the reduced variability of O-Full near ~27.7 kg m⁻³. In response to this observation, we performed an additional composite analysis to examine the relationship between O-East and O-West transport anomalies (Fig. R1). This analysis shows that O-East and O-West exhibit partial compensation at similar densities, including near ~27.7 kg m⁻³.

We interpret this behavior as consistent with a density-space shift of compensation patterns along the subpolar pathway. OE-Out exhibits a compensation structure similar to that in O-West but centered at slightly broader and lighter densities. As a result, basin integration can reveal opposing contributions from O-East and O-West at similar densities, leading to partial cancellation.

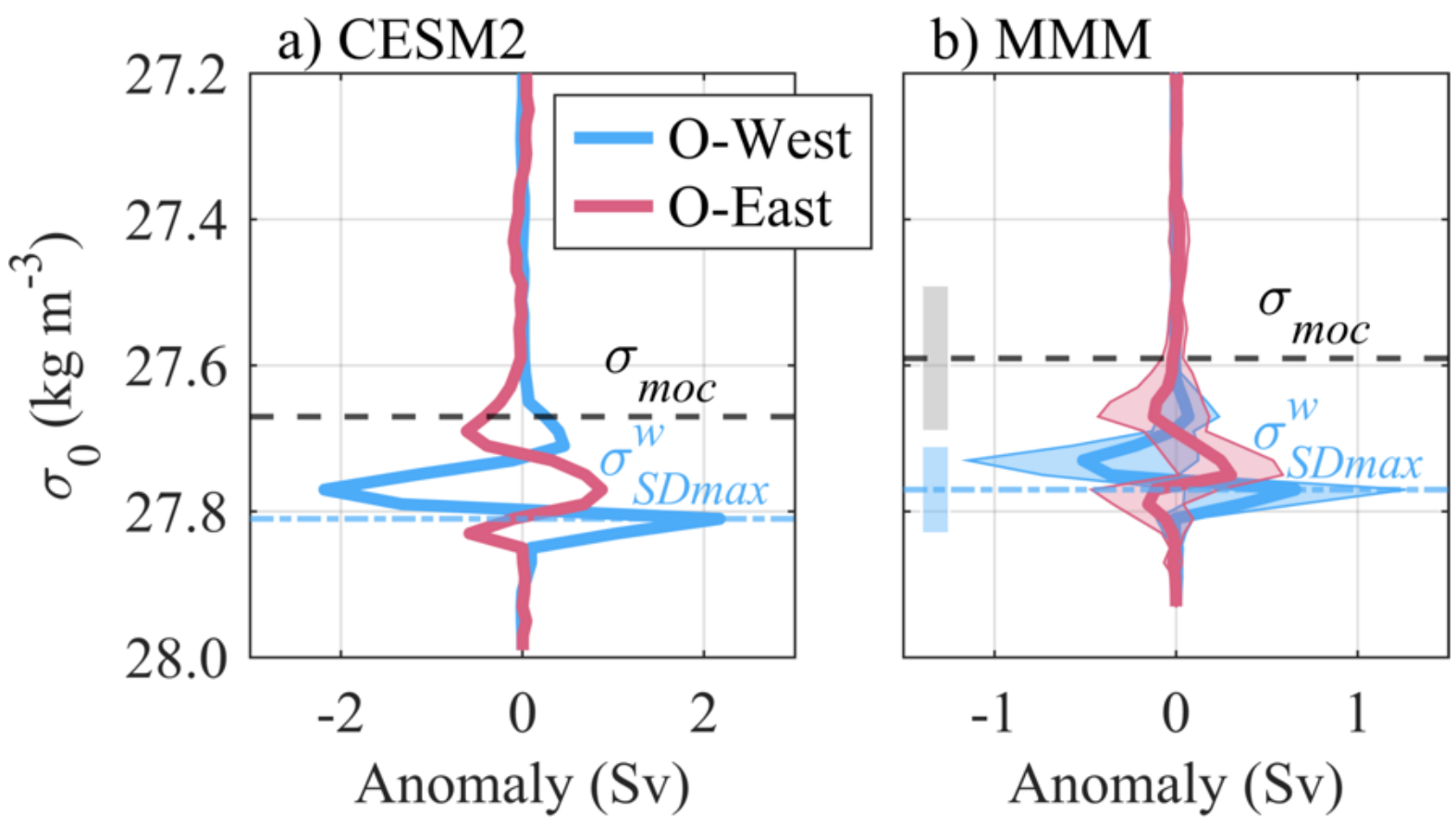

Fig. R1. Compensation pattern of low-frequency transport for O-West and O-East. a) A composite of discrete transport anomalies constructed using transports at each density when there is a positive transport anomaly at σ^{w}_{SDmax} . **b)** As in a), but for the multimodel mean (MMM). In the construction of the multimodel mean results, each model distribution is shifted in density space such that all σ^{w}_{SDmax} are aligned.

Reviewer 2: The authors examine AMOC transport and variability in the subpolar latitudes using long simulations from 9 CMIP6 models and make comparisons on short timescales with OSNAP observations. They find that subpolar AMOC variability is predominantly driven from the Irminger and Iceland basins, with contributions from the Labrador Sea being smaller and generally providing a role as an amplifier of variability which originates upstream. The text and figures are presented to a high standard, although some recent and relevant literature seems to be missing (e.g. Megann et al., 2021, Jackson and Petit, 2023 Petit et al., 2025). However, as it is currently presented the manuscript does not contain sufficient new knowledge. It needs to more clearly define the research question(s) it seeks to address and hence the conclusions which can be drawn from the subsequent analysis.

We thank the reviewer for this constructive assessment and appreciate the concern regarding the novelty and clarity of the research questions in the original submission. In response, we have substantially refocused and reorganized the manuscript to more clearly articulate the central questions addressed by this study and the new insights that emerge from our analysis.

Specifically, based on the reviewer's suggestion, the revised manuscript aims to identify which components of the subpolar overturning circulation, and which regions of density space, most effectively capture the local and downstream AMOC variability across timescales. We demonstrate how strong variability in dense subpolar waters can be largely compensated in density space, thereby limiting its projection onto AMOC strength. We have also expanded the discussion to place our results in the context of recent literature, including the studies suggested by the reviewer, and to clarify how our findings provide a mechanistic framework that helps reconcile differing perspectives on the role of the Labrador Sea in AMOC variability.

Major comments:

1) Overall, the analysis focuses on correlations of time series at σmoc (maximum overturning at a time varying density) and $\sigma SDmax$ (overturning at a time invariant density located the maximum standard deviation for each section), yet the manuscript does not make it clear to readers why we should expect a relationship to exist between the monthly transports at $\sigma SDmax$ and the net northward transport of the upper limb (MOC, as defined in this study) across the OSNAP sections. In failing to do so, the message of the paper is muddled and it is not clear what novel conclusions this paper delivers. The main conclusion, that variability on longer timescales is dominated by the Irminger and Iceland basins and not the Labrador Sea is not new (see e.g. Megann et al., 2021), and several previous studies have reported that the depth of maximum variability is found below the depth of the maximum transport at all latitudes (e.g.

Hirschi et al., 2020 and references therein). I don't believe there is a rigorous explanation as to why this is the case in published literature, but I wonder if you are close with this work, noting that the deep convection in the Labrador Sea introduces additional variability to water masses that are already below the density of the maximum AMOC.

We thank the reviewer for this important comment, which has helped us clarify both the motivation and the novel contribution of this study.

The motivation for focusing on σ_{SDmax} is not that we expect variability at this density to control the AMOC. Rather, σ_{SDmax} emerges objectively as the density at which transport variability is strongest across models and observations. This naturally raises two questions: 1) what the source of this variability and 2) whether such strong dense-water variability serves as a meaningful indicator of local and /or downstream AMOC variability on monthly and decadal time scales. The suggestion in Yeager et al. (2021) that strong variability in dense waters associated with Labrador Sea water mass transformation may precede downstream AMOC variability provides further motivation for an examination of how dense-water variability projects onto overturning strength.

Accordingly, in the revised manuscript we focus on identifying which density classes exhibit the strongest relationship with local and downstream AMOC variability, rather than privileging σ_{SDmax} itself. We acknowledge that two aspects of our results are consistent with previous studies: subpolar AMOC variability is dominated by the Irminger and Iceland basins rather than the Labrador Sea (e.g. Megann et al., 2021); and the depth or density of maximum overturning variability lies below that of maximum transport across a wide range of latitudes and models (e.g. Hirschi et al., 2020).

Here, we extend these studies by demonstrating that these behaviors are robust across multiple CMIP6 models, across monthly to multidecadal timescales, and is also evident in OSNAP observations. Importantly, we provide a mechanistic interpretation showing that strong dense-water variability—whether originating upstream or modified within the Labrador Sea—largely cancels in density space and therefore has a limited impact on overturning strength.

2) On Line 291-292: "we find strong variability at $\sigma SDmax$ has little influence on the overall MOC variability on both interannual and multidecadal timescales". This appears to be at odds with Yeager et al., (2021) - whose numerical simulation is included in the model ensemble used in this study – who found that "...low-frequency variability of density-space overturning at 45°N, which accounts for almost all the low-frequency variance in ocean heat transport into the SPNA, is driven by large amplitude fluctuations in the density distribution of southward-flowing lower limb transport."

Importantly, Yeager et al. (2021) demonstrate this relationship through a lagged composite analysis of the diapycnal overturning and Labrador Sea surface water mass transformation rather than correlating the diapycnal overturning strength at 45° N with that taking place at $\sigma SDmax$ across OSNAP West. This leads me to question whether a similar relationship might be uncovered if a lagged correlation / composite analysis were used in this study. The physical mechanism proposed by Yeager et al. (2021) results in a time delay between the lower and upper limb anomalies of roughly a decade at ~55° N.

We appreciate the need to directly address what appears to be a result "at odds' with Yeager et al. To that end, we have now added lead–lag correlation analyses between subpolar transport anomalies and the downstream MOC at 50°N in the revised manuscript (Fig. 4; Supplementary Fig. 9). These analyses show that allowing for lags does not strengthen the relationship between basin-integrated OSNAP-West overturning and downstream MOC in the multimodel mean, consistent with recent results reported by Petit et al. (2025).

However, when the western basin is decomposed into northward and southward transport components, the southward component (OW-Out) exhibits a clearer lagged relationship with the downstream MOC, consistent with Yeager et al. (2021), who highlighted the role of southward-flowing lower limb transport.

Importantly, our analysis further shows that this lagged variability in OW-Out is strongly correlated with upstream transport variability (OE-Out and OW-In), indicating that much of the signal expressed in the Labrador Sea outflow is inherited from upstream rather than generated locally. While Yeager et al. emphasized the role of Labrador Sea water-mass transformation, our results highlight that upstream variability can account for a large fraction of the transport signal in Labrador Sea.

3) Several approaches could be taken to improve the clarity of the manuscript's central message. Firstly, the authors could broaden their analysis to consider which regions of density-space are most important for capturing MOC (or better still meridional heat transport variability, since this is what is climatically important). This would be a refocusing of the current analysis, which asks are transports at $\sigma SDmax$ related to the MOC strength, to instead address which regions of the diapycnal overturning stream function might best capture the variability at the isopycnal of maximum diapycnal overturning. Alternatively, the authors could explore whether transport variability at $\sigma SDmax$ is able to capture downstream MOC variability (as found by Yeager et al., 2021) despite being poorly correlated with local, subpolar diapycnal overturning variability. This might suggest that the communities' strong focus on the variability found at the isopycnal of maximum overturning at OSNAP is not necessarily where the subpolar overturning exhibits its strongest relationship to downstream (say 45° N) MOC variability. Reshaping the manuscript with this focus would allow the authors to address

whether the relative stability of the subpolar MOC, which is briefly discussed on Lines 306-309, is a further artifact of using the stream function maxima (since this conceals variability in the composition of the upper limb).

We thank the reviewer for this exceptionally constructive comment, which has helped us sharpen our central scientific questions and the structure of the manuscript. We agree that, as originally written, the analysis could be perceived as being overly focused on whether variability at σ_{SDmax} itself should correlate with the MOC, without sufficiently motivating why variability at this density is of interest.

In response, we have substantially refocused and reorganized the manuscript to address the broader and more physically meaningful question: which regions of density space, and which components of the overturning circulation, most effectively capture AMOC variability across time scales, both at the OSNAP section and farther downstream. We now show correlations between AMOC variability and transport variability across the full density space, explicitly identifying where correlations are strongest and where they are weak. We believe this refocus substantially improves the clarity of the manuscript and more clearly articulates the novel contribution of the study.

Minor comments:

1) The first section of the results opens with two paragraphs introducing and describing the OSNAP observations, but then focuses solely on analysis of the 9 CMIP6 models between lines 78 and 244. This transition is confusing at first, and there is scope to reorganise the section to improve readability.

We understand the confusion and, in response, have reorganized the opening of the Results section to clarify the structure of our study. The revised text now explicitly frames the study around the OSNAP observing system at the outset, before presenting model-based results. We then clearly state that the primary analysis focuses on long preindustrial control simulations, with CESM2 and MPI-ESM1-2-HR used for illustration and a multimodel framework used for the main results. OSNAP observations are now explicitly introduced as an independent benchmark. We believe this reorganization improves readability and clarifies the respective roles of models and observations in the study.

2) Line 71: "The nine CMIP6 models were selected for their relatively low biases in bottom density" – It is worth expanding further, both on how the models were selected and on the limitations of CMIP-class models. I can’t see how the 9 models were objectively identified from Heuze, 2021 on the basis stated. Looking at their Fig. 3, which shows bottom density biases and RMSE, why, for example, was CanESM5 selected over the EC-Earth, MRI-ESM2-

0 or CNRM models? CMIP-class models are in general not capable of representing key processes like the dense overflows from the Nordic Seas (CESM2's overflow parameterisation excepting) and often have unrealistically vigorous deep convection in the Labrador Sea, so they are unlikely to exhibit low biases in bottom density for the right reasons.

We agree that the original wording was insufficiently precise. We now clarified in the revised Methods (lines 541-549):

"We used Heuzé et al. (2021) as a broad reference to exclude models with extreme bottom-density biases in the Greenland–Iceland–Norwegian Seas and subpolar gyre, which could otherwise dominate density-space structure and hinder inter-model comparison. Beyond this exclusion of clear outliers, no selection was made based on overturning behavior and the final ensemble represents a quasi-random subset spanning a range of ocean model formulations and horizontal resolutions. Accordingly, our analysis does not rely on the fidelity of any individual model, but instead emphasizes relationships that are consistent across models, time scales, and independent observations"

Thus, our model selection is not preferring CanESM5 over EC-Earth, MRI-ESM2-0, or CNRM models. The ensemble deliberately includes models with a range of bottom-density biases, including CanESM5, which exhibits a larger—but not extreme—bias. The persistence of key features across this ensemble demonstrates that our conclusions are not contingent on accurate mean-state density structure or on models simulating subpolar processes in an optimal way.

Accordingly, while CMIP-class models are known to have limitations in representing key subpolar processes, including Nordic Seas overflows and Labrador Sea convection, our analysis does not rely on their ability to reproduce a realistic mean state. Instead, it focuses on relationships and structural features of variability that are consistent across models, time scales, and independent observations.

3) Line 200: It is unclear in this paragraph whether the time series are the total northward (southward) transport across the section (Lines 200-203). Figure 4a shows anomalies. How do you calculate the anomalies (removal of initial value, mean, line of best fit, …?)?

We apologize for the ambiguity. In the revised manuscript, we have clarified that the time series shown in Fig. 3a represent the directional transport anomalies across each section, evaluated near σ_{SDmax} by integrating transport over the two density bins immediately below σ_{SDmax} (lines 201–210).

The "anomalies" in original Fig. 4 (now Fig.3a) refer to the mean-removed, low-frequency component of these transport time series. Specifically, the raw transport time series are first

demeaned and then low-pass filtered following the procedure described in the Methods (lines 454-465).

4) Related, flipping the sign of OW-In would make visual comparison (e.g. in Fig 4A, D) with the other two lines and correlation values much easier. The labelling of the curves implies positive into the Labrador Sea for “OW-In” and positive out of the Labrador Sea for “OW-out”. It is not surprising that OW-In and OW-out are so highly correlated with each other, since the Labrador Sea can be regarded as almost a closed basin. There can only be minimal change in storage so the difference between inflow and outflow will be dominated by flows through the Hudson and Davis Straits. Therefore, the interesting aspect is not correlation between the inflow and outflow, but the differences in the water mass properties of those two flows.

In the revised figures, we now reverse the sign of OW-In to facilitate direct comparison among OE-Out, OW-In, and OW-Out and to simplify interpretation of the correlation values (see revised Figs. 3a, 5d).

The purpose of the inflow–outflow decomposition is to examine how variability expressed at specific density levels is preserved and modified as waters transit the Labrador Sea. Our diagnostics are not based on transport integrated over the full water column, but on transport evaluated at selected densities or integrated from the bottom to a given density. In this framework, OW-In and OW-Out are not a priori required to exhibit identical variability, because water mass transformation and other processes can shift variability toward other density classes and alter its amplitude.

Consistent with this interpretation, we find that variability near σ_{SDmax} in the outflow is closely linked to upstream variability near σ_{SDmax} there, while being shifted toward denser densities and amplified during transit through the Labrador Sea (Fig. 3). This behavior indicates that a substantial fraction of the variability expressed in the Labrador Sea outflow is inherited from upstream, with local modification in density space—an aspect emphasized throughout the revised manuscript.

5) Line 263: suggest “…also offset each other on monthly timescales.”

We have accepted the suggested wording.

6) Line 263-4: Please clarify this statement. Don’t the high (and presumably significant?) correlations between OW-In, OW-Out and OE-Out, and the fact that variability is largest below σ_moc, support the argument that a single boundary may be sufficient to capture the overall MOC strength?

We apologize that the original wording was overly general and did not directly demonstrate how transport variability at individual boundaries relates to the overall MOC strength.

In the revised manuscript, we extend the analysis to explicitly quantify the relationship between transport anomalies at each directional transport component and the MOC. We find that both OW-In and OW-Out exhibit moderate correlations with the OSNAP MOC, reaching values of up to ~0.55, whereas the basin-integrated transport across O-West shows substantially weaker correlations (maximum ~0.20). In this sense, transport variability at a single boundary in O-West can better reflect MOC variability than the basin-integrated O-West signal.

However, this behavior is not universal across the OSNAP sections. On the eastern boundary, OE-Out also shows correlations of order ~0.55 with the MOC, but these remain weaker than those obtained using the basin-integrated transport across O-East ($r \approx 0.65$). This indicates that a single-boundary transport does not, in general, provide a superior proxy for MOC strength relative to basin-integrated measures. We have revised the text accordingly to reflect this more nuanced interpretation and to avoid overgeneralizing the implications of transport compensation (lines 269-274).

7) Line 292-94: "Specifically, the multi-model mean correlation coefficients..." Please clarify whether the correlation coefficients are calculated on the multi-model mean, or whether this is the ensemble mean of the correlation coefficients computed on each of the models separately.

We thank the reviewer for requesting clarification. The correlation coefficients are computed separately for each model, after applying the same low- or high-frequency filtering described in the Methods, and the reported values represent the multimodel mean of these individual-model correlations. We have clarified this calculation procedure in the revised Methods section.

8) Typos:

L142: "raging" > "ranging"

L167: Missing "The" > "The large black circle…"

We thank the reviewer for noting these typographical issues. We have carefully reviewed the revised manuscript and corrected these and other minor typographical errors.

References:

Hirschi, J. J.-M., Barnier, B., Böning, C., Biastoch, A., Blaker, A. T., Coward, A., et al. (2020). The Atlantic meridional overturning circulation in high-resolution models. Journal of Geophysical Research: Oceans, 125, e2019JC015522. https://doi.org/10.1029/2019JC015522

Jackson, L.C., Petit, T. North Atlantic overturning and water mass transformation in CMIP6 models. Clim Dyn 60, 2871–2891 (2023). https://doi.org/10.1007/s00382-022-06448-1

Megann, A., Blaker, A., Josey, S., New, A., & Sinha, B. (2021). Mechanisms for late 20th and early 21st century decadal AMOC variability. Journal of Geophysical Research: Oceans, 126, e2021JC017865. https://doi.org/10.1029/2021JC017865

Petit, T., Robson, J., Ferreira, D., Yeager, S., & Evans, D. G. (2025). Coherence of the AMOC over the subpolar North Atlantic on interannual to multiannual time scales. Geophysical Research Letters, 52, e2025GL115171. https://doi.org/10.1029/2025GL115171

Reviewer #3 (Remarks to the Author):

I co-reviewed this manuscript with one of the reviewers who provided the listed reports. This is part of the Nature Communications initiative to facilitate training in peer review and to provide appropriate recognition for Early Career Researchers who co-review manuscripts.

Reviewer #4 (Remarks to the Author):

The manuscript titled "The Labrador Sea as a conduit of AMOC variability on multiple time scales" by Chen and Lozier uses climate simulations to look at variability of the Atlantic meridional overturning circulation (AMOC) in the subpolar North Atlantic (SPNA), calculated both at σ(MOC) the maximum overturning density, and σ(SDmax), the density with the highest standard deviation. The aim of the study is to reconcile previous observational results that show the eastern SPNA dominates overturning variability with model studies that had suggested an important role of the Labrador Sea in the western SPNA in setting longer-term variability.

The authors find that σ(MOC) transport is dominated by O-East even on longer time scales, and although the peak standard deviation of the overturning stream function is set by variability in the O-West section, this largely results from transport changes within the lower AMOC branch, with little effect on overall strength of the AMOC.

This study is a valuable addition to the recent literature discussing the role of water mass transformation and overturning in the eastern and western SPNA, and represents an important step in understanding what drives multi-decadal AMOC variability. While I think the results are robust, I do feel some improvements need to be made in how they are presented, and the methods and results should be clarified by providing more information about the calculations and improving the phrasing of some paragraphs.

We thank the reviewer for the positive assessment of our manuscript and for recognizing its contribution to understanding AMOC variability in the subpolar North Atlantic. We have carefully revised the manuscript to improve clarity in the presentation, methods, and phrasing, as suggested. Specific comments are addressed in detail below.

General comments:

1) I think the methods and materials section should have a bit more detail about how the calculations were done, currently I find it difficult to fully follow how the main results were derived. In particular:

- How was the σ(SDmax) transport/transport anomaly actually calculated? I believe the "normal" OSNAP transport is calculated by integrating the equation in line 345 in density space from the lightest density to σ(MOC). So is the SDmax transport in e.g. Fig. 2A the integral from the surface to σ(SDmax)? Or just the transport in some density range around σ(SDmax)?

- Similarly, I couldn't find any mention of how the partitioning into southward and northward flow in Fig,. 4/5D is achieved; Is it summing together all the positive transport values at a given time step for OW-In and all the negative ones for OW/OE-Out? Is it a certain segment of the OSNAP line, e.g. OW-In is the eastern half of OSNAP-West and OW-Out is the western half?

We agree that additional methodological clarification was needed. In the revised manuscript, we have expanded the Methods section to clearly distinguish between the different transport diagnostics used in the analysis.

As for the calculation of the σ_{SDmax} transport/transport anomaly: We now explain in the text on lines 439-441:

"Because the streamfunction is defined as the cumulative transport integrated upward from the bottom, the streamfunction value at σ_{SDmax} corresponds to the transport integrated from the bottom to σ_{SDmax}."

As for the partitioning of the southward and northward flow: We now explain in the text on lines 201-205:

"Using the same composite approach, we analyze directional transport anomalies across three connected sections: the total southward transport across O-East (OE-Out), the total northward transport into O-West (OW-In), and the total southward transport exiting O-West (OW-Out) , each evaluated on density layers near σ_{SDmax}."

2) For the discussion, the finding of "inherited" upstream variability in this study fits nicely with recent work on the continued transformation of surface into deep waters in the subpolar gyre (e.g. Buckely et al 2023,

https://royalsocietypublishing.org/doi/full/10.1098/rsta.2022.0181; Tooth et al 2025, https://egusphere.copernicus.org/preprints/2025/egusphere-2025-1132/) and it would be good to discuss that connection a bit more in the paper.

We thank the reviewer for this helpful suggestion. In the revised Discussion, we have expanded the interpretation of the "inherited" upstream variability to more explicitly connect our results with recent studies emphasizing the cumulative nature of water-mass transformation across the subpolar gyre. Specifically, we now highlight that the persistence of upstream variability across most models is consistent with recent work showing that dense waters exported from the Labrador Sea reflect a cumulative history of transformation across the broader subpolar gyre, rather than formation within a single localized region (e.g., Buckley et al., 2023; Tooth et al., 2025). See lines 389-393.

Specific comments:

Line 30-33: This statement makes it sound like overflow waters are not part of NADW, which I don't think is true. I would say that NADW is composed of lighter waters formed by open ocean convection (LSW/uNADW) and denser overflow waters (DSOW & ISOW/lNADW)

We have revised the text to clarify that open-ocean convection in the subpolar North Atlantic primarily forms upper NADW, while denser components of NADW originate as overflow waters from the Nordic Seas. See lines 31-34.

Line 41-42: The statement could be strengthened by adding phraseing connecting the statements about Pickart et al (ref. 11) and Lozier et al (ref. 12). E.g. something like "Consistent with their findings, direct observations from OSNAP revealed…"

We have revised the text to more explicitly connect the findings of Pickart et al. (11) with the OSNAP observations by clarifying that the latter are consistent with and build upon the earlier hydrographic estimates. See lines 43-44.

Line 56-58: I don't' have a specific suggestion here, but I think the motivating questions could be phrased a bit better

We have substantially refocused and clarified the motivating questions. See lines 64-71.

Line 60-65: Again, I think this paragraph could be reworded a bit to better set up the rest of the study. For example, there's no need to start with "Motivated by these questions" after

the previous paragraph talked about "questions that motivate our study". Just saying "To address these questions…" would sound better

We have revised the paragraph to avoid repetitive phrasing (lines 72-74).

Line 102 (Fig. 1): I think this is a nice figure, but could be further improved with some changes:

- I can infer from the text that the figure shows CESM2 model data, but it would be good to explicitly mention that in the caption. It could also be useful to mention the model time frame (300 years?) to provide context for the LF and HF filtering

- I don't see any colorbar, or even a mention of what the colors show (salinity?), in panel A

- It could be confusing to some readers to have red/blue for northward/southward flow in panel A and using a slightly different shade of red and blue for O-West and O-east

- It could be helpful to add a line fo σ(SDmax) in panels C and E; but I also understand that it might make the figure too busy, so it's fine to leave it out

We have revised the figure caption according to these suggestions. We have also clarified in the caption that the shading in panel (a) represents salinity and we have added a colorbar. Regarding the color scheme, we agree that there is no need to indicate the inflow and outflow by different colors since they are already distinguished by circle with dots and circles with cross. We agree that adding σ_{SDmax} to panels (c) and (e) could be informative; however, we chose not to include it to avoid visual overcrowding.

Line 135-136: Are the "lagged downstream responses" shown somewhere in the SI, or is this just an extra calculation you did that is not shown?

We have now systematically examined lead–lag correlations, in particular between transports at the OSNAP section and downstream AMOC variability and revised the text accordingly. The associated lag times are now provided in the Supplementary Information.

Line 157 (Fig. 2): Add σ(MOC) to legend in panel C to be consistent with panel A. Also panel B and D should either be expanded to show a range from -1 to 1, or the zero line should be highlighted, to make interpretation more intuitive

In the revised manuscript, Fig. 2 has been substantially revised and now presents correlation-based diagnostics rather than the original transport time series. As a result, the specific issues raised here regarding the legend and axis ranges are no longer applicable.

In the revised Fig. 2, σ_{moc} and σ_{SDmax} are explicitly indicated in all panels, and the correlation coefficients and transport anomalies are shown on consistent and clearly labeled axes to facilitate comparison across density space and panels.

Line 167-168: Describe small black/gray circles too – presumably the correlation between O-Full and 50N for individual models

This point refers to the original version of Fig. 2, which has been replaced in the revised manuscript and no longer contains the symbols mentioned here.

Line 171-172: The first two sentences could be combined and shortened

We have combined and reworded the two sentences to improve clarity and be concise. See lines 174-178.

Line 184-185: “changes in local water mass transformation occurs” doesn’t sound right – do you mean either ““changes in local water mass transformation” or “changes in where local water mass transformation occurs”?

In the revised manuscript, this sentence has been removed and replaced with a more direct description based on the decomposition of transport anomalies.

6 Line 215: It would be good to provide some explanation here for why you chose to show GFDL data; is it just to show how things look different in a different model? Is it because the GFDL model is a unique case with most of the change apparently happening between OE-Out and OW-In, or is this representative of other models?

Also, is the implication here that GFDL has relatively little transformation both across OSNAP East and OSNAP-West, but a lot of transformation SE of Greenland?

In the original manuscript, we used the GFDL model simply to show how things looked in a different model. It was not meant to be representative of all models.

As for your second question, the implication for this model is that much of the transformation occurs between OE-Out and OW-In. However, this was not meant to suggest that transformation is weak across OSNAP East. In the revised manuscript, we no longer use the GFDL model alone for interpretation. Instead, we focus on results that are consistent across the multimodel ensemble and use CESM2, along with MPI-ESM1-2-HR, to show representative behavior.

Line 232 (Fig. 4):

- As for fig. 1, it could be useful to visually show σ(SDmax) somehow. Maybe a thin dashed line in the colors of each transport component in panels B and C?

- "Of" sounds odd in the phrases like "southward flow of O_East", "northward flow of O_West", could be replaced by something like "across" or "through"

- The colors in panel D are confusing since they are related to, but not quite the same as, panels A-C, so it would be good to add a legend. Alternatively you could add y tick labels on the right side of the axis to label each row of correlations, in which case you don't actually need to differentiate colors at all

In the revised manuscript, Fig. 4 has been substantially restructured and replaced with a new set of diagnostics focusing on transport– downstream MOC relationships. As a result, the specific presentation issues raised here are no longer directly applicable. Nevertheless, the revised figures have been designed with these comments in mind. In particular, the locations of both σ_{moc} and σ_{SDmax} are now explicitly indicated where relevant.

Line 259-261: Currently panel 5 D is not mentioned in the text, so it should be referenced here

In the revised manuscript, Fig. 5 has been restructured and the panel referred to here is no longer present. However, we have now ensured that all figure panels in the revised manuscript are explicitly referenced in the text.

Line 265: What are the 71 months shown in figure S7? Is this just one particular 71-month period out of the 300 years? If so how was it chosen? It's fine not to state this in the main text but it should be mentioned in the S7 caption

The 71-month time series shown in the original Fig. S7 corresponds to the length of the OSNAP record available at the time of our earlier analysis and represents a single continuous period, shown for illustrative purposes only. It is not a particular 71-month period, but rather a set of randomly selected 71-month periods over the 300-year model simulation. In the revised manuscript, we no longer use this monthly variability model–observation comparison, as it is not essential to the revised analysis. In this revision, we have focused the presentation on ensemble-robust features and observational consistency, rather than on single-model validation of monthly variability.

Line 278: I'd prefer mentioning what this shows instead of just saying "As in Fig. 4A"; i.e. "Time series of transport anomalies at σ(SDmax) as in Fig. 4A". Also since this is using observational data it would be good to state what time period the 71 months correspond to

In the revised manuscript, the figure referred to here has been replaced, and the corresponding issue no longer applies. However, per your comment, figure captions now explicitly describe what is shown, rather than referring only to other panels. Also, the observational time period is now clearly stated in the caption.

Line 287-289: Mention the value of the isopycnal in question

In the revised manuscript, this statement has been removed as part of a reorganization of the Discussion, and we no longer explicitly link σ_{moc} to a specific water-mass transformation density. We note, however, that the multimodel-mean value of σ_{SDmax} (≈ 27.75) is reported in the revised manuscript to provide quantitative context.

Line 310-325: As mentioned above, it would be good to put this paragraph into the context of some other studies like Buckley et al 2023 and Tooth et al 2025

We have revised this paragraph to explicitly place our results in the context of recent studies, including Buckley et al. (2023) and Tooth et al. (2025). See lines 389-393.

Line 329-330: Here too you could mention previous studies; e.g. that the dominant role of O-East variability further underscores the findings from Lozier et al 2019

We have revised the text to explicitly place this result in the context of previous studies, including Lozier et al. (2019), that emphasize the dominant role of the eastern subpolar basins in AMOC variability. See lines 368-371.

Line 322-323: "even at monthly timescales" – isn't it only at monthly time scales? Since the LF and HF variability as defined for the models isn't resolved at present by OSNAP

We thank the reviewer for pointing this out. We have revised the wording to clarify that the observational results refer to variability resolved at monthly timescales only.

Line 333-334: Could be phrased better "the OSNAP time series limits our ability to understand AMOC variability on longer timescales" makes it sound like OSNAP measurements are actively detrimental to understanding AMOC variability, which would be an unfortunate finding. I think it's better to say that OSNAP alone is insufficient to understand AMOC variability on these timescales, or simply that they are not (yet) resolved by OSNAP

We have revised the wording to clarify that OSNAP observations are limited in the timescales they resolve, rather than implying that they hinder understanding of longer-timescale AMOC variability.

Line 350: Should specify what σ0 is, i.e. potential density referenced to the surface

We have now made this specification.

Line 365: I think you should reference Table S1 again here, and it could also be useful to provide the names of all the models somewhere in this section.

We now explicitly refer to supplementary Table 1 in this section (lines 79-82, 532-535).

Line 378: It would be good to add here, or somewhere else in the materials and methods, some more info about the OSNAP data used, including the time period covered in the current version. I also think it would be good to add a reference either for the data product or for an earlier OSNAP paper describing how it is calculated since there are quite a few steps between the actual measurements and the gridded T/S/v product used here

We have expanded the Methods section to provide additional information on the OSNAP data used, including the period covered in the revised manuscript, and have added appropriate references describing the OSNAP data products and their processing. See lines 521-531.

Line 381-383: Is the 0.08 kg/m3 offset applied to the data shown in the study (or fig. S8)? If not, maybe it's better to phrase it differently than "after aligning the profiles", which sounds like an offset was applied to the data. Also, it's unclear what the "average coefficient of 0.83" refers to

We have revised the text to clarify that a small uniform density offset is explicitly applied only for this diagnostic comparison, and that it does not affect any analyses or figures elsewhere in the study. We have also clarified that the reported value of 0.83 refers to the mean pattern

correlation coefficient (rather than a time-series correlation), computed separately for O-Full, O-East, and O-West and then averaged across the three sections.

Line 387-389: Need some more context for “the modeled range of 71-month means”. Does this mean splitting the 300-year time series up into 71-month segments and calculating the mean for each? Is the range the lowest to highest value of those means? I’m also not sure what the “range of monthly SD” is, and in any case, there are no values provided either for it or the observed value it’s compared to, so the statement isn’t too useful.

I think it could be easier to follow if you just state the mean and SD of the 71-month means, compared to the mean and uncertainty of the observed value.

Yes—the “range” shown by the gray dashed curves corresponds to the minimum and maximum across these segment means.

In the revised manuscript, we reassessed the role of the model–observation comparison and concluded that it was not essential to the central aims of the study. We therefore removed this material in the revised manuscript and refocused the presentation on ensemble-robust features and observational consistency, rather than on single-model validation of monthly variability. As a result, the quantities referred to in the reviewer’s comment are no longer shown or discussed in the manuscript.

Line 388: No need to define Sverdrup again since you already did in line 353

We have removed the repeated definition of ‘Sverdrup’ in the revised manuscript.

Reviewer #1 (Remarks to the Author):

This is a much improved version of the manuscript and it is generally well written. The paper makes an important point that variability in dense water transport is not necessarily linked to the downstream AMOC strength variability because compensation can occur in density space.

However, much of the current discussion remains largely descriptive. In my view, the more fundamental issue is understanding what controls the density of maximum AMOC variability (sigma_SDmax) and why it differs from the density of maximum overturning (sigma_moc). The manuscript repeatedly demonstrates that compensation occurs in density space, but this essentially restates the result that sigma_SDmax > sigma_moc, rather than explaining why this relationship arises dynamically.

In addition, the discussion of how the present results reconcile with Yeager et al. (2021) is not sufficiently convincing. I am also not persuaded by the authors' conclusion that the Labrador Sea acts only as a conduit for AMOC variability, particularly for density classes close to sigma_SDmax (see specific comments below).

We thank the reviewer for noting the improvements to the manuscript. We attempt in this revision to explain more clearly what sets sigma_SDmax, why it differs from sigma_moc, and how these results relate to Yeager et al. (2021). We address each of these issues below.

Specific Comments

1. **Interpretation of sigma_SDmax vs. sigma_moc**: While it is useful to show that variability in dense water transport does not necessarily translate into downstream AMOC strength variability, the more important question is what controls sigma_SDmax and why it differs from sigma_moc. Given a perturbation in the water mass transformation, compensation of transport variability across density classes is expected in density space. Therefore, the discussion of compensation largely reiterates the finding that sigma_SDmax>sigma_moc, rather than addressing the dynamical reason why this occurs. The manuscript would be strengthened by a clearer explanation of the processes that set these two density scales and why they differ.

We thank the reviewer for raising this point. We agree that the original manuscript did not sufficiently discuss the dynamical reason why sigma_SDmax is denser than sigma_moc. We now explain that a plausible mechanism is linked to the behavior of the dense winter outcropping classes. We have examined surface-forced water mass transformation (SFWMT) in density space and found that the location of the variance maximum is linked to much stronger temporal variability in the winter outcrop area of dense density classes (Fig. R1). This interpretation is also consistent with our decomposition of the transport compensation pattern in Fig. 2c, d, which shows that opposing transport anomalies across densities are dominated by area-driven contributions in both the eastern and western subpolar basins (Lines 196–198).

While sigma_SDmax identifies where the variability is largest, sigma_moc reflects the density at which the cumulative overturning streamfunction reaches its maximum after compensation across adjacent density classes and redistribution by mixing. We have added this discussion to the revised manuscript (Lines 402–404).

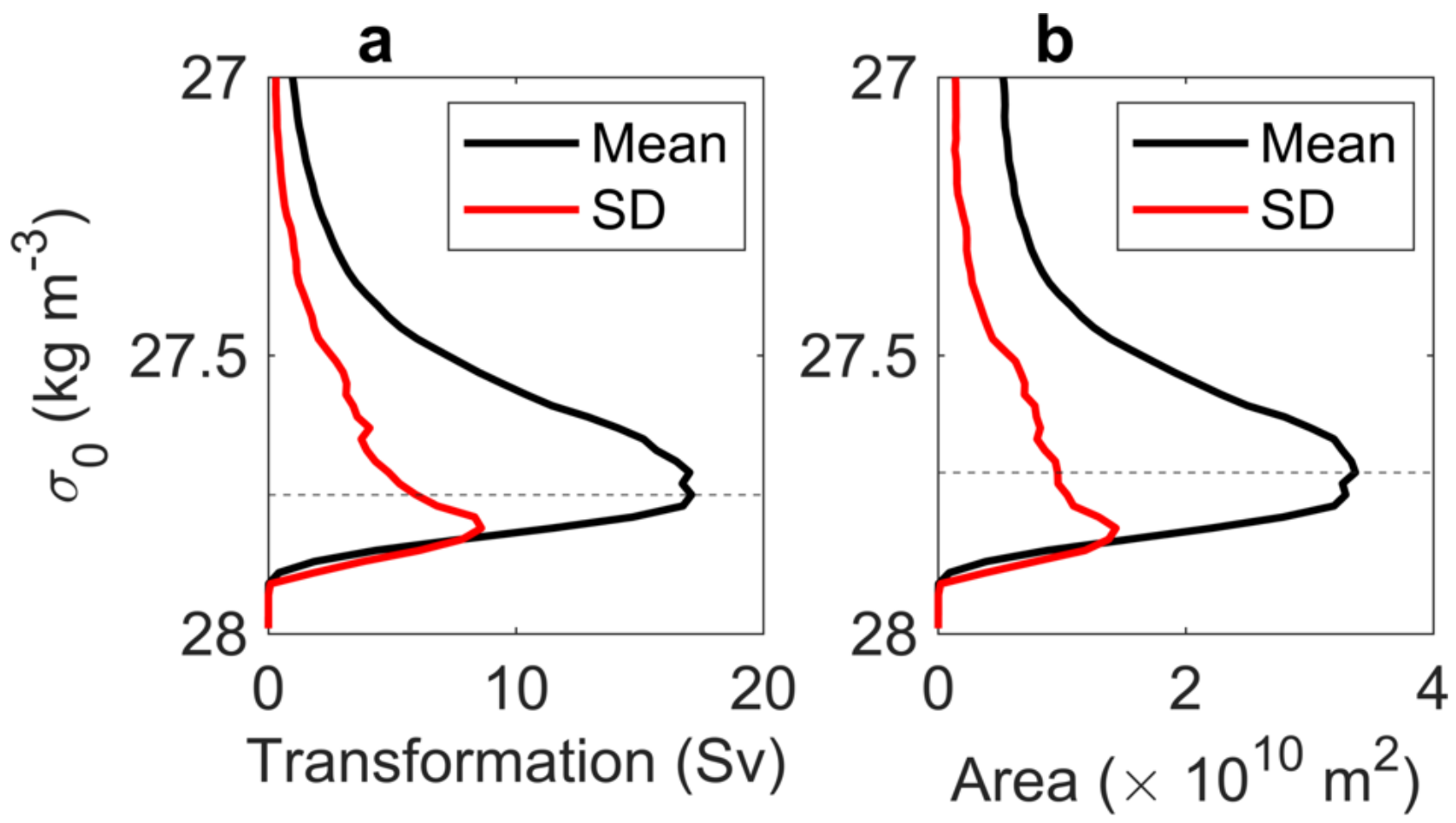

Fig. R2. Mean state and variability of surface-forced water mass transformation and outcrop area of Labrador Sea. **a** Surface-forced water mass transformation as a function of surface density. **b** Outcrop area as a function of surface density. Black curves show the time mean and red curves show the temporal standard deviation. For both the transformation and the outcrop area, the density of maximum variability is denser than the density of the maximum mean.

2. **Source of deep water transport variability**: To examine the origin of dense transport variability, the authors plotted transport anomalies near sigma_SDmax for three components in Figure 3a: southward transport exiting O-East, northward transport entering O-West, and southward transport exiting O-West. Based on the correlations among these three components, the authors suggest that the transport variability originates in OSNAP-E and is subsequently advected into the Labrador Sea, where further modifications occur.

My difficulty arises from comparing Figure 3a with Figure 1d,f. Figure 1d,f suggest that the transport variaiblity near sigma_SDmax associated with O-East is negligible compared to that in O-West. This implies that the AMOC variability near sigma_SDmax reflect water mass transformations within the Labrador Sea. It would be helpful to also include the northward transport entering O-East (OE-In) in Figure 3a. It is highly likely that substantial compensation also occur between OE-In and OE-Out. If such compensation exists, would this mean that the Irminger Sea is also a conduit for AMOC variability?

I agree that the downstream AMOC variability (in terms of its strength) is mainly associated with OSNAP-E, as shown in previous studies. However, the overturning variability near sigma_SDmax may still originate from Labrador Sea water mass transformations (surface plus interior). If this interpretation is correct, the authors should be more precise about the density range when discussing the origin of AMOC variability.

We thank the reviewer for this careful comment. Figure 1 and Figure 3 are not directly contradictory, because the O-East signal is weak at the density where O-West and O-Full reach their variance maximum, but O-East exhibits its own variance maximum at lighter densities. We now clarify this explicitly in the text. (Lines 107-109). Note that the pathway analysis in Figure 3 evaluates each branch in the density range near its own branch-specific sigma_SDmax, rather at the same sigma_SDmax.

We also followed the reviewer's suggestion to examine OE-In and OE-Out. O-East does show incoming–outgoing coherence when evaluated at its own sigma_SDmax, but this coherence is substantially weaker than in O-West (0.41 for O-East vs. 0.87 for O-West), indicating that the pathway-scale structure near sigma_SDmax is most strongly expressed in O-West. We have revised the manuscript to reflect this new analysis (Lines 227-231).

Finally, we address your concern about whether transport variability near sigma_SDmax may originate from Labrador Sea water mass transformations in our response to your concern #3. See below.

3. **Relationship to Yeager et al. (2021)**: The discussion of how these results relate to Yeager et al. (2021) is currently ambiguous. Yeager et al. (2021) showed a connection between surface forced dLSW formation and AMOC strength variability on multi-decadal timescales, while this paper finds little correlation between AMOC strength and dense water transport.

As the authors noted in the introduction, the dense water transport variability is not necessarily the same as surface forced water mass formation. However, it remains unclear why the surface forced water mass formation correlates well with the AMOC in Yeager et al. (2021).

This may be reconciled by combining the discussions in Yeager et al. (2021) with the results shown in Figure 4b of this study.

Your question about whether overturning variability near sigma_SDmax can be attributed (at least in part) to Labrador Sea water mass transformation prompted us to thoroughly examine the SFWMT–AMOC relationship across our model ensemble. Specifically, for each model we calculated the correlation between SFWMT variability across the Labrador Sea and OW-Out transport variability and compared that to the correlation between OW-In and OW-Out transport variability. As shown in a new Supplementary figure (7), OW-In explains substantially more variance in OW-Out than Labrador Sea SFWMT alone (multimodel-mean R^2 of 0.90 versus 0.46). Thus, these results show that while the dense signal near sigma_SDmax reflects both inherited upstream signals and subsequent modification within the Labrador Sea, OW-In has a stronger linkage.

In addition to evaluating the model-mean statistics, to answer your specific question we also looked at the statistics for the model used in Yeager et al. (2021). For this model, we find R^2 =0.54 for SFWMT with OW-Out versus R^2 =0.80 for OW-In with OW-Out, showing that while Labrador Sea surface forcing does explain *part* of the downstream AMOC signal, it is *weaker* than the corresponding pathway transport relationship. Fig. 4b shows the impact of OE-Out, OW-In, and OW-Out on the downstream AMOC, a testament to the strength of this pathway transport relationship.

In summary, we appreciate that Yeager et al. (2021) identified SWFMT as an upstream source mechanism for downstream AMOC variability. Our results show that SFWMT is indeed *one* source of variability, however the larger source of variability in the model used in Yeager et al. (2021) and in all other models stems from the advection of upstream signals.

We have revised the manuscript to reflect this new analysis (Lines 236-246).

References

Jackson, L.C. and Petit, T. (2023). North Atlantic overturning and water mass transformation in CMIP6 models. Clim Dyn 60, 2871–2891.

Megann, A., Blaker, A., Josey, S., New, A., & Sinha, B. (2021). Mechanisms for late 20th and early 21st century decadal AMOC variability. Journal of Geophysical Research: Oceans, 126, e2021JC017865.

Fu, Y., M. S. Lozier, S. Majumder, & T. Petit, Water mass transformation and its relationship with the overturning circulation in the eastern subpolar North Atlantic. J. Geophys. Res. Oceans 129, e2024JC021222 (2024).

Reviewer #2 (Remarks to the Author):

Please see the attached document: "Review of revised Chen and Lozier.pdf". Copied here:

Review of revised version of “The Labrador Sea as a conduit of AMOC variability on multiple time scales” by Chen and Lozier

The authors have addressed all the concerns raised. There are, however, a few points where the manuscript would benefit from further clarification or discussion:

The use of OE-Out, OW-In and OW-Out in the Discussion needs to be reviewed. As it stands, it is included in the discussion as if these quantities represent inflow and outflow contributions to the diapycnal overturning at OSNAP East, West & 50N. However, they are simply volume transport anomalies in a density range near sigma_SDmax. The description in the figure 3 caption (lines 236-237) states “Transport anomalies are computed by integrating over the two density bins just below $\sigma SDmax$.”. This range should be included in the text more explicitly rather than relying on Supplementary Information. It is not clear how this fits with lines 476-480, which describe the terms as “the total southward transport across O-East (OE-Out), the total northward transport into O-West (OW-In), and the total southward transport exiting O-West (OW-Out)”. By referencing equation (1) at the end of the paragraph it creates the impression that the terms are a cumulative integral from the maximum density to $\sigma SDmax$. Please review the text carefully to ensure that the reader is not misled.

We thank the reviewer for raising this point and agree that the previous wording could mislead the reader. In Fig. 3, OE-Out, OW-In, and OW-Out are not intended to represent inflow/outflow contributions to the overturning streamfunction. Rather, they are directional, section-integrated transport anomalies evaluated in a limited density interval near the branch-specific sigma_SDmax, used to trace the pathway-scale evolution of dense anomalies. We now clarify this explicitly in the main text, Methods, and Fig. 3 caption. We also added an analysis showing that the same pathway coherence is obtained when transport is cumulatively integrated from the bottom to the branch-specific sigma_SDmax.

2) Can you be more precise as to what extent this is boundary current vs. interior phenomenon (alluded to on line 222 but not elaborated on thereafter). Do these isopycnal layers outcrop at the sea surface in the boundary current, if not why would we expect the layer thickness to change significantly along-stream (mainly isopycnal mixing in the Labrador Sea boundary current - interior region)?

We agree that this needed to be stated more precisely. In the revised manuscript, OE-Out, OW-In, and OW-Out are defined as full-section directional transports within a limited dense range near the branch-specific sigma_SDmax, whereas the boundary-current version is treated

separately as an along-isobath diagnostic integrated within 200 km offshore of the 500-m isobath (Supplementary Fig. 8). We now clarify this distinction explicitly in the text. (Lines 253-256)

We do not interpret variability near sigma_SDmax as arising solely from local outcropping or transformation within the boundary current. Rather, layer-thickness changes can reflect both local boundary-current processes and anomalies formed in the Labrador Sea interior that are transferred into the boundary current. This interpretation is consistent with previous studies showing that dense anomalies formed in the Labrador Sea interior can be transferred to the boundary current and exported downstream along density surfaces (Zou et al. 2020), while boundary-current transformation and even convection in the boundary current itself can also contribute (Bebieva and Lozier 2023; Palter et al. 2008; Georgiou et al. 2020).

3) Are the transport anomalies present on outflow at OSNAP East (OE-Out) a reflection of surface buoyancy forcing in the Iceland and Irminger basins? Where do these layer thickness anomalies originate from? This is missing from the discussion currently.

We now clarify that these anomalies are consistent with upstream buoyancy-forced transformation in the Iceland and Irminger basins. We now refer to a previous study which has shown that surface forcing makes a major contribution to the OSNAP-East lower limb (Petit et al., 2020). We also refer to another recent study that shows that anomalies formed in the Irminger basin interior can be transferred into the boundary current and exported downstream within months (Le Bras et al., 2020). In summary: we now make clear that anomalies exiting OE-Out result from water mass transformation in the eastern subpolar basin (Lines 406-410).

Finally, we ask the reviewer to see our response to Reviewer 1's concern #2. For that response we followed that reviewer's suggestion to examine OE-In and OE-Out. As we write above: "*O-East does show incoming–outgoing coherence when evaluated at its own sigma_SDmax, but this coherence is substantially weaker than in O-West (0.41 for O-East vs. 0.87 for O-West), indicating that the pathway-scale structure near sigma_SDmax is most strongly expressed in O-West. We have revised the manuscript to reflect this new analysis (Lines 227-231).*"

4) Is one of the implications of lines 381-384, that quantifying basin-scale stream functions in density compensated regions is partially flawed, and that focusing on then outflow of the Labrador Sea (DWBC) may be a more useful indicator of downstream AMOC variability? This fits with the text on lines 414-416, but perhaps your results suggest that outflow is a better choice than lower-limb variability?

We agree that one implication of our results is that, in Labrador Sea, laterally integrated overturning metrics may obscure pathway-scale signals. However, we do not conclude that Labrador Sea outflow is the best overall indicator of downstream AMOC variability. When basin-wide section data are available, the strongest relationship with downstream AMOC variability is found for the O-East or O-Full overturning metrics. When only boundary-focused diagnostics are available, OW-In, OW-Out, and OE-Out provide useful and broadly comparable pathway information, without indicating a unique advantage for Labrador Sea outflow alone. We have revised the discussion accordingly (Lines 307-312, lines 442-446).

5) Eq 1 should use a dummy integration variable (σ') to be mathematically consistent:

$$\Psi(\sigma) = -\int_{x_w}^{x_e}\int_{\sigma_{max}}^{\sigma} v(x,\sigma')\,d\sigma'\,dx$$

We have revised Eq. (1) according to your suggestion. Thank you.

6) Lines 550-553: During this interpolation do you also rotate the velocity components to be normal to the section?

Yes, during the interpolation the velocity components are rotated so that they are normal to the section. We now state this explicitly in the Methods.

7) Figure 11 (suppl.): y-axis labels are missing

We have added the missing y-axis labels to Fig. 11 in the Supplementary Information.

References

Zou, S., Lozier, M.S. Li, F., Abernathey, R., and Jackson, L. (2020). Density-compensated overturning in the Labrador Sea, Nature Geoscience, doi: 10.1038/s41561-019-0517-1.

Bebieva, Y., and Lozier, M. S. (2023). Fresh Water and Atmospheric Cooling Control on Density-Compensated Overturning in the Labrador Sea. Journal of Physical Oceanography, 53(11), 2575-2589.

Palter, J.B., Lozier, M.S. and Lavender, K. (2008). How does Labrador Sea Water enter the Deep Western Boundary Current? Journal of Physical Oceanography, 38, 968-983.

Georgiou, S., Ypma, S. L., Brüggemann, N., Sayol, J. M., Pietrzak, J. D., & Katsman, C. A. (2020). Pathways of the water masses exiting the Labrador Sea: The importance of boundary–interior exchanges. Ocean Modelling, 150, 101623.

Petit, T., Lozier, M. S., Josey, S. A., & Cunningham, S. A. (2020). Atlantic deep water formation occurs primarily in the Iceland Basin and Irminger Sea by local buoyancy forcing. Geophysical Research Letters, 47, e2020GL091028.

Le Bras, I. A.-A.; Straneo, F., Holte, J., Jong, M. F., Holliday, N. P. (2020). Rapid export of waters formed by convection near the Irminger Sea's western boundary. Geophysical Research Letters, 47 (3), e2019GL085989.

Li, F., Lozier, M.S., Bacon, S. et al. (2021). Subpolar North Atlantic western boundary density anomalies and the Meridional Overturning Circulation. Nature Communication, 12, 3002.

Reviewer #3 (Remarks to the Author):

I co-reviewed this manuscript with one of the reviewers who provided the listed reports. This is part of the Nature Communications initiative to facilitate training in peer review and to provide appropriate recognition for Early Career Researchers who co-review manuscripts.

Reviewer #4 (Remarks to the Author):

REVIEW OF "The Labrador Sea as a conduit of AMOC variability on multiple time scales", Chen et al 2026, revision 1

I think the authors did a great job responding to the comments by myself and other reviewers, and the revised manuscript is much clearer in its motivation, scope, and key results. I don't have any significant comments in terms of content at this point, but did notice some areas where the text could be changed to further improve the clarity and readability of the paper, as outlined below.

We thank the reviewer for this positive assessment and for the helpful suggestions to further improve the clarity and readability of the manuscript. We respond to specific points below.

MINOR COMMENTS

L 44-45: "direct observations two decades later of the meridional overturning circulation via the Overturning in the Subpolar North Atlantic Program" - could improve flow of this sentence; maybe "two decades later" is not necessary

We agree and have revised the sentence for smoother flow by removing "two decades later".

L 81: Should reference be earlier? e.g. "(CMIP6 (17); Supplementary Table 1)" instead of "(CMIP6; Supplementary Table 1) (17)"

We have revised the citation placement accordingly.

L 86: It could be helpful for readers not familiar with OSNAP to say explicitly that O-West + O-East = O-full; for example, change the text to "across a full section (O-Full) *comprised of* two coast-to-coast subsections (O-West and O- East; Fig. 1a)."

We have revised the text accordingly.

L 100: "as with observations": Again, this could be changed to improve clarity for some readers; as far as I can tell the paper doesn't actually include the OSNAP observational streamfunction anywhere either in the main text or supplementary, and while the authors and many readers will be intimately familiar with it, it could be helpful to include for those that aren't. Alternatively, at least a reference to an OSNAP paper that shows the streamfunction would be useful

We agree that the original wording was too abbreviated. Rather than adding the observational streamfunction to the manuscript, we revised the sentence to refer explicitly to the OSNAP observational estimates and added the citation in the text.

"*Consistent with OSNAP observations (13), overturning at O-West is markedly weaker than at O-East, confined to a narrower density range, and reaches its maximum at a higher density*"

L 102: "Looking downstream" - It could be useful to add a nominal latitude for OSNAP, which of course might be difficult since it spans a relatively broad range. But some indication

on the figure could help put this in context; either with a symbol or line at a representative latitude, or showing the lat range covered by OSNAP

We now indicate the approximate latitudinal extent of the OSNAP array in Fig. 1 and clarify this in the caption. We also revised the text to replace “Looking downstream” by “*Turning to the zonally integrated overturning streamfunction*”.

L 116-117: “We next confirm this indication” - I think it would be better to say something like "we further explore this connection" instead to avoid already giving a conclusion to results that are only shown in the next section

We have revised the wording and the text now reads: “*We next test this more directly ...*”.

L 125/Fig 1a: I appreciate the additon of the colorbar but think a little more information is still needed: Is the potential density shown taken from observations or CESM2? And is it a time-mean over the whole period?

We agree and have clarified in the Fig. 1a caption that the shading shows time-mean potential density from CESM2.

L 141: “we examine where in density space we might find a strong relationship.” - could be phrased slightly better

We have revised the sentence for clarity. It now reads: “*To identify where in density space transport variability is most strongly linked to overall MOC variability, we calculate the correlation between the full OSNAP MOC and the overturning streamfunction integrated from the bottom to each density level across the OSNAP sections (Fig. 2a).*” (Lines 142-145)

L 143/Fig 2a: Why doesn't the black line equal 1 at sigma_moc? Is it because for OSNAP MOC the density is time variable and here a constant density is used?

Yes, the sigma_moc for OSNAP MOC is a time-varying sigma, whereas the correlations in Fig. 2a are evaluated at fixed density surfaces. In addition, Fig. 2a shows the multimodel mean. As a result, the O-Full correlation does not necessarily reach 1.0 at the multimodel-mean sigma_moc.

L 160/Fig 2: It would be good in the figure caption to note again that this is CESM2 data

We agree that this should be stated explicitly. Fig. 2 shows multimodel-mean results, and we have revised the caption to make this clear.

L 165/Fig 2: add panels in the description of c and d to improve clarity; e.g. "decomposed into contributions from area anomalies (c) and velocity anomalies (d)"

We have revised the caption to identify panels **c** and **d** explicitly as the area- and velocity-contribution decompositions, respectively.

L 207/Fig 3a: Reading this I was immediately wondering why the authors switched from showing the CESM2 model in fig 1/2 to MPI-ESM1-2-HR in fig 3. The reasoning is stated in the methods in L 555-557: "MPI-ESM1-2-HR is shown in Fig. 3a because the correlation between OW-Out and OW-In in this model is close to the multimodel mean, making it a representative example of the ensemble behavior." It might be a good idea to add some explanation along those lines in the main text to avoid readers thinking the model may be "cherry-picked" for this figure

We agree that this should be stated in the main text. We now clarify that MPI-ESM1-2-HR is shown in Fig. 3a because its OW-Out and OW-In correlation is close to the multimodel mean, making it a representative example rather than a selectively chosen case. Revised text: "*We show results from MPI-ESM1-2-HR (Fig. 3a) because the correlation between OW-In and OW-Out for this model is closest to the multimodel mean.*" (Lines 218-219)

L 239/fig 3b: Could be helpful to add a legend to the panel, or a description in the text, since the colors are not strictly the same as panel a; i.e. purple line is OE-out/OW-out correlation, and orange line is OW-in/OW-out correlation,

We have revised the caption to identify the purple line as the OE-Out–OW-Out correlation and the orange line as the OW-In–OW-Out correlation.

L 285/Fig 4: Should this be "black dash-dotted line" rather than "black solid line"?

We agree and have corrected "black solid line" to "black dash-dotted line" in the caption.

L 291-294/Fig 5: It could be useful to compare this shorter timescale variability to model data; in fact I believe the authors did so in the original manuscript (fig. S7 in original supplementary) but now took out that analysis. I think it would be valuable to see that models

and observations show similar patterns on short timescales, supporting the use of the models for the longer-term analysis in Fig 3.

Also, since panels b and c are similar to previous figures on the CESM2 data (fig 2a, b) it would be good to at least briefly relate these, even though the time scales may be different

We agree that the connection between the OSNAP monthly results and the shorter-timescale model variability should be stated more explicitly. We have therefore restored the CESM2 monthly variability comparison as Supplementary Fig. 12 and revised the main text to briefly link Fig. 5 more directly to both the CESM2 monthly results and the earlier model results (Fig. 1 and Supplementary Figs. 3, 12) (Lines 331-344).

L 368-369: I would remove the phrase “this is not unexpected”; just saying it's consistent with previous studies should be sufficient

We have removed “this is not unexpected.”

L L427-433: Could improve flow of this paragraph by moving up the sentence about model velocity/property fields before the mention of GSW/potential density calculation, and also make it clearer initially that model data is also extracted along OSNAP, as the phrasing "while for the streamfunction across zonal sections (which we use with model output)" could be interpreted to mean ONLY zonal sections are used in models. You could clarify this by adding a similar clause at the beginning of the sentence "For the streamfunction across each OSNAP section (used both with models output and observations)"

We agree that the original wording could be read as implying that model output is used only for zonal sections. We have revised this paragraph to make clear that model output is used for both the OSNAP sections and the zonal sections. We have also reordered the text. (Lines 484-488)

L 481-493: It could be useful to have separate subsections for this area/velocity decomposition and the preceding OE-Out/OW-In/OW-Out decomposition. Maybe this could also be merged with the earlier introduction of V (444 - 452)?

We agree that the transition between the definition of density-bin transport and its area/velocity decomposition could be clearer. Rather than introducing additional subsections, we merged the area/velocity decomposition with the introduction of density-bin transport. (Lines 510-519)

L 495: It could be helpful to mention the density offset for each model e.g. in a table; alternatively the multimodel sigma_SDmax could be marked in fig S1 so readers can visually see the offset

We agree that additional guidance on model-to-model density offsets is helpful. We now mark the multimodel mean sigma_SDmax in Supplementary Figs. 2 and 3, which allows the reader to see the offset of each model relative to the multimodel reference directly.

L 569/Table S1: For reproducibility it would help to add which CMIP6 variables were downloaded (T, S, v?) and which model variant is used for each model, i.e. identifiers with the format r1i1p1f1

We agree that these details are important for reproducibility. Because the same CMIP6 variables and variant label were used for all CMIP6 models, rather than adding new columns we clarified this directly in the Supplementary Table 1 caption. The caption now states that the downloaded CMIP6 variables are thetao, so, uo, and vo, and that the variant label used is r1i1p1f1 for all CMIP6 simulations.

Reviewer #1 (Remarks to the Author):

I appreciate the authors' effort to address my concerns, but I remain unconvinced that the AMOC variability at σ_{SDmax} originates primarily from the Iceland and Irminger basins.

Consider the isopycnal transport across OSNAP-Full, T_σ (e.g., between $[\sigma_{\text{SDmax}} - \delta\sigma]$ and σ_{SDmax}; positive southward)—this connects to lower-latitude AMOC variability (Fig. 1c). The AMOC variability at σ_{SDmax} in Figure 1d,f primarily reflects variations in T_σ, which is the sum over OSNAP-West and OSNAP-East:

$$T_\sigma = T_\sigma^W + T_\sigma^E,$$

where $T_\sigma^W = T_\sigma^{\text{W-Out}} - T_\sigma^{\text{W-In}}$ and $T_\sigma^E = T_\sigma^{\text{E-Out}} - T_\sigma^{\text{E-In}}$. These residuals are modulated by water mass formation, driven by surface forcing and interior mixing, and local volume changes between isopycnals within each basin.

The variance in T_σ decomposes as:

$$\text{Var}(T_\sigma) = \text{Var}(T_\sigma^W) + \text{Var}(T_\sigma^E) + 2\text{Cov}(T_\sigma^E, T_\sigma^W).$$

Figure 1d,f shows the AMOC variability near σ_{SDmax} is primarily associated with T_σ^W, with negligible contributions from T_σ^E and the covariance. This points to processes within the Labrador Sea, not the Iceland/Irminger basins, noting that it is the residual betwen inflow and outflow, not the outflow itself, that is related to the overturning.

The core issue: the authors appear to equate AMOC variability with variability in $T_\sigma^{\text{W-Out}}$ rather than T_σ. This is incorrect for OSNAP-Full. Across a constant latitude it is also incorrect, because $T_\sigma^{\text{W-Out}}$ exiting the Labrador Sea only partially accounts for the lower branch of the AMOC. This is easily testable: compare $T_\sigma^{\text{W-Out}}$ variability to the net AMOC transport variability at the same latitude or across OSNAP-Full. They will differ substantially.

I am also concerned that different models were used for Figure 1 vs. Figure 3—CESM2 has a stronger Labrador Sea overturning than MPI-ESM1-2-HR and OSNAP observations. However, this does not change the central point even if MPI-HR is used to reproduce Figure 1: the Labrador Sea makes a sizable contribution to AMOC variability in all models examined except for ACCESS and CanESM5 (Supplementary Figure 2).

In summary, the results do not support the conclusion, at least not the claim that the Labrador Sea acts as a conduit for AMOC variability. I would be happy to see rebuttals from the authors, but if they agree with the points above, the conclusion should be overhauled accordingly.

We thank the reviewer for clarifying this concern and for laying out the residual-budget interpretation so explicitly. We agree with the reviewer's framework: in some models, the O-West contribution near σ_{SDmax} is large and can dominate the OSNAP-Full variability at that density, as also noted in the original manuscript (Lines 110-111).

This comment helped us recognize that our previous wording/definitions did not sufficiently distinguish among three related but different quantities: (i) AMOC strength variability, (ii) streamfunction or integrated-transport variability at σ_{SDmax}, and (iii) Labrador

Sea OW-Out/In transport variability. In our manuscript, AMOC/MOC strength variability means variability of the maximum overturning streamfunction, ψ_{max}, at σ_{moc}. Therefore, "AMOC variability at σ_{SDmax}" should be called streamfunction/integrated transport variability at σ_{SDmax}, or overturning variability at σ_{SDmax}. We apologize for this confusion.

We also agree that $T^{\text{OW-Out}}$ is not equivalent to the net OSNAP-Full MOC metric and it was never our intent to make this equivalence. As the reviewer points out, we agree that the Labrador Sea contribution to the net overturning streamfunction at σ_{SDmax} is associated with the residual between inflow and outflow, and that this residual is shaped by local Labrador Sea processes, including water-mass transformation, mixing, storage, and density shifting. As such, we do not claim that local (i.e., Labrador Sea) *overturning* variability near σ_{SDmax} is primarily inherited from the Iceland and Irminger basins.

The question addressed by our study has a different focus: we are interested in whether the strong Labrador Sea variability identified near σ_{SDmax} is the component most closely linked to downstream MOC variability and to the OSNAP-Full MOC strength variability. The comparison suggested by the reviewer is already partly shown in the manuscript: Fig. 4 shows that transport anomalies exported out of the Labrador Sea are correlated with downstream MOC strength, with a maximum correlation of ~ 0.62. We have now stated explicitly that the same Labrador Sea exported transport anomalies are also correlated with OSNAP-Full MOC strength, with a comparable correlation of ~ 0.54 (Supplementary Fig. 6b). Thus, although Labrador Sea outflow is not the net MOC metric, it carries transport variability that is statistically linked to both downstream and OSNAP-Full MOC strength. In contrast, the Labrador Sea *local* overturning residual, despite its large variance near σ_{SDmax}, is only weakly related to OSNAP-Full MOC and the downstream MOC strength. Please see lines 297-305 of our manuscript for our description of this difference.

Therefore, our interpretation of the study results does not rely on equating Labrador Sea outflow with AMOC metric. Instead, our study shows that **Labrador Sea inflow/outflow transport variability provides a link between Iceland-Irminger transport variability and downstream AMOC/MOC strength variability, while Labrador Sea local overturning variability near $\boldsymbol{\sigma_{SDmax}}$ is largely shaped by local transformation processes**. Based on your concern:

1. We have revised the title to avoid implying that the Labrador Sea is a passive conduit for the *net* AMOC/MOC overturning anomaly. Our original use of "conduit" was motivated by the finding that Labrador Sea incoming and exported transport anomalies are significantly correlated with both downstream MOC and OSNAP-Full MOC strength, and that the exported Labrador Sea transport anomalies are themselves strongly linked to transport anomalies leaving OSNAP East and entering the Labrador Sea. In that sense, the Labrador Sea provides a pathway through which transport variability connected to the Iceland–Irminger basins is linked to

downstream AMOC/MOC strength variability. However, we now recognize that the word "conduit" could be misleading as we did not sufficiently distinguish Labrador Sea local overturning variability from Labrador Sea inflow/outflow transport variability. We have therefore revised the title.

2. We have also revised the text in order to clear up ambiguity noted above. Please see lines 433-438.

Finally, regarding the use of different models in Figs. 1 and 3: we agree that this should be clarified. CESM2 was used in Fig. 1 because the enhanced streamfunction variability below σ_{moc} is particularly clear in this simulation. However, we agree with the reviewer that CESM2 has stronger Labrador Sea overturning variability near σ_{SDmax} than MPI-ESM1-2-HR and stronger than many of the other CMIP6 models and OSNAP observations. As such, we agree that highlighting this model could be misleading, if not stated explicitly. We have therefore revised the text to clarify that CESM2 is an illustrative example rather than a representative case in terms of amplitude. We now write (lines 105-107): "*Because the amplitude of Labrador Sea variability in CESM2 is larger than in many other models, we use it here to illustrate the density-space structure of the variability, rather than as a representative case in terms of amplitude*."

The qualitative result does not depend on CESM2. Similar density-space structures are found in other models, including MPI-ESM1-2-HR, as shown in Supplementary Figs. 2-3. Importantly, even in CESM2, where Labrador Sea streamfunction variability near σ_{SDmax} is especially strong, the Labrador Sea local overturning residual does not stand out as being more strongly correlated with OSNAP-Full or downstream MOC strength. This supports our interpretation that large Labrador Sea local overturning variability near σ_{SDmax} should be distinguished from Labrador Sea transport anomalies that are linked to MOC strength variability.

As stated in the original manuscript, Fig. 3 uses MPI-ESM1-2-HR because its OW-Out/OW-In correlation is close to the multimodel mean, making it a representative example for illustrating the transport-variability relationship.

In closing, we would like to thank the reviewer again for the careful review of our paper. We believe that the clarifications we have added to address your concerns have strengthened the paper.

Reviewer #2 (Remarks to the Author):

I am happy that all the points I raised have been satisfactorily addressed.

Reviewer #3 (Remarks to the Author):

I co-reviewed this manuscript with one of the reviewers who provided the listed reports. This is part of the Nature Communications initiative to facilitate training in peer review and to provide appropriate recognition for Early Career Researchers who co-review manuscripts.

Reviewer #4 (Remarks to the Author):

I thank the authors for addressing my comments, and have no further suggestions at this time.

We thank Reviewers #2 and #4 for their positive assessments and are pleased that they have no further concerns or suggestions. We also thank Reviewer #3 for their contribution to the review process through the Nature Communications co-review initiative.

Reviewer #1 (Remarks to the Author):

I appreciate the authors' clarifications. This is the fourth round of review, and my only remaining requirement for acceptance is resolving a contradiction between Figure 1 and Figure 4–this can be achieved by replacing Figure 1 with a model that better aligns with the multi-model mean.

The paper's main conclusion is that the Labrador Sea acts as a passive conduit for anomalies originating in OSNAP-East. However, the choice of CESM for Figure 1 directly contradicts this narrative and the multi-model results in Figure 4.

By definition, OSNAP-Full = OSNAP-West + OSNAP-East. In Figure 1, the variance in OSNAP-West dominates the total budget. A straightforward mathematical derivation dictates that the component dominating the variance must yield the higher correlation with the total sum.

Therefore, the physics shown in Figure 1 for CESM2 demand that OSNAP-West is the primary driver of full AMOC variability. This completely contradicts Figure 4, where the ensemble mean shows OSNAP-East dominating the correlation.

Once Figure 1 is replaced to align with the text and Figure 4, the manuscript will be structurally sound and ready for acceptance.

We thank the reviewer for identifying this remaining concern. We understand that the CESM2 example in Fig. 1 visually emphasizes strong Labrador Sea variability near σ_{SDmax}, and that this could be misread as suggesting that O-West controls OSNAP-Full MOC strength variability.

To address this concern, we have revised the manuscript to make it clear that:

1) AMOC/MOC strength is defined as the maximum of the density-space overturning streamfunction, with the corresponding density denoted as σ_{moc}.

2) As such, variability near σ_{SDmax} , as shown in Fig. 1, is *not* equivalent to AMOC/MOC strength variability.

3) Thus, the strong O-West contribution near σ_{SDmax} in CESM2 does *not* imply that O-West is the primary driver of OSNAP-Full MOC strength variability. In fact, Fig. 1 shows that OSNAP East dominates the variability at σ_{moc}.

We have decided to retain CESM2 in Fig. 1 because it nicely illustrates the density-space structure of the variability, which is central to the manuscript: strong variability in deep waters exiting the Labrador Sea is not strongly linked to AMOC variability across the subpolar ocean

and downstream. At the same time, we now state explicitly that CESM2 is used as an illustrative case rather than as representative in terms of amplitude. To address the reviewer's concern and to show that the conclusions do not depend on this illustrative case, we have added an aligned multimodel composite as a new Supplementary Fig. 3.

We have also added the following text:

1. "Here and throughout the manuscript, MOC strength refers to the maximum of the density-space overturning streamfunction, with the corresponding density denoted as σ_{moc}. As such, the standard deviation shown in Fig. 1 represents variability of the overturning streamfunction at each density, not variability of the MOC strength."

2. "This peak is contributed primarily by O-West, while O-East reaches its own LF SD maximum of about 2.34 Sv at a lighter density. The dominance near σ_{SDmax} therefore reflects a strong Labrador Sea overturning variability at these dense densities rather than a dominant contribution of O-West to overall MOC strength variability."

3. "Having shown that strong variability near σ_{SDmax} does not by itself imply strong variability in the overall MOC strength, we next examine more closely the relationship between transport at σ_{SDmax} and MOC variability."

Finally, for the reviewer's consideration, we note that OSNAP-Full is not the sum of OSNAP-West and OSNAP-East MOC strengths. The MOC strength is a maximum of the streamfunction and the relevant density levels can differ among sections (e.g. 27.63 for OSNAP West, and 27.55 for OSNAP East; Lozier et al. 2017, Yao et al. 2023).

Reference:

Lozier, M. S., S. Bacon, A. S. Bower, S. A. Cunningham, M. F. De Jong, L. De Steur, et. al. Overturning in the Subpolar north Atlantic program: A new international ocean observing system. Bull. Am. Meteorol. Soc. 98, 737–752 (2017).

Fu, Y., M.S. Lozier, T.C. Biló, et al. Seasonality of the Meridional Overturning Circulation in the subpolar North Atlantic. Commun Earth Environ 4, 181 (2023).

Reviewer #1 (Remarks to the Author):

This is the fifth round of review. While the authors have provided additional clarifications, a fundamental misalignment remains between the primary figures and the main conclusion. Given that multiple rounds of review have not resolved this structural contradiction, I recommend that the manuscript be rejected as further revisions are no longer productive.

The manuscript retains key logical and structural contradictions that need to be addressed on a purely physical basis:

1. Misalignment of figures and conclusion

The authors are entirely correct in their response that the MOC strength does not simply add up across sections, as the maximum overturning occurs at different densities (σ_{moc}) for different regions. However, my previous comments were not referring to the peak MOC strength (Ψ_{max}). I was explicitly referring to the continuous, density-space overturning streamfunction, $\Psi(\sigma)$.

By fundamental definition, at any given density layer σ, the full array streamfunction is the exact sum of its components:

$$\Psi_{\text{Full}}(\sigma) = \Psi_{\text{West}}(\sigma) + \Psi_{\text{East}}(\sigma).$$

When evaluating the streamfunction profile rather than the peak strength, the add-up works exactly. In CESM2 (Figure 1), a variance decomposition demonstrates that OSNAP-West dominates the AMOC streamfunction variance at σ_{SDmax}. This mathematically requires that the Labrador Sea is the primary driver of the overturning variability at this dense layer in this model, which directly contradicts the study's main conclusion that the basin acts as a passive conduit.

In Figure 1, the authors acknowledge that OSNAP-West dominates the streamfunction variance at the dense lower-branch layer (σ_{SDmax}). This means the Labrador Sea is the primary generator of lower-branch streamfunction variability in this model, flatly contradicting the paper's core thesis that the basin is merely a passive "conduit."

Nevertheless, the authors insisted on keeping this figure–this is not acceptable.

2. Methodological Mismatch: Lower-Branch Transport vs. Maximum MOC Strength

There is a disconnect between the stated focus of the paper and the metrics used in the analysis. The title and abstract highlight the role of OSNAP-East in driving the *lower-branch transport* of the AMOC. However, in Figures 3 and 4, the analysis relies on correlations between the overall maximum AMOC strength (Ψ_{max}) and the subpolar overturning streamfunction at each individual density level.

If the goal is to evaluate lower-branch transport anomalies, these point-by-point correlations against the maximum MOC strength are unnecessary and introduce a methodological mismatch. The conclusions regarding the subpolar ocean's role would be more directly and transparently supported by simply plotting the variance of the overturning streamfunction across density levels for the ensemble.

We thank the reviewer for their continued persistence in this fifth round of review. We appreciate the opportunity to further clarify the physical and methodological framework of our study. We agree that at a fixed density, the density-space streamfunction can be examined in terms of regional contributions, and that in CESM2, the O-Full streamfunction variance near σ_{SDmax} is contributed primarily by O-West.

However, we respectfully but firmly clarify that a mathematical variance decomposition at a fixed density layer identifies "where" variability is expressed; it does not, by itself, identify "where or how" that variability is dynamically generated or originated.

To address the reviewer's concern directly on a purely physical basis, we respectfully direct the reviewer's attention to our sequential upstream-to-local breakdown already presented in Figure 3 of the manuscript. The O-West streamfunction is decomposed into two components OW-In and OW-Out, and any strong signal observed near σ_{SDmax} is intrinsically determined by the variations and interplay of the two individual components, regardless of which component happens to dominate at a given time or in a specific model. By tracking the multi-model ensemble-mean signals from the transport exiting OSNAP-East (OE-Out) to that entering the Labrador Sea (OW-In), and finally exiting it (OW-Out), the physical mechanisms become transparently clear:

1. Pre-existing Upstream Signatures (Figure 3c).

The prominent 'alternating positive/negative' compensating pattern in density space is not locally manufactured by the Labrador Sea. Instead, this exact vertical structural fingerprint is already fully imprinted and pre-existing in the upstream OE-Out transport. As the anomalies transit through the O-West domain, this pre-existing upstream signal is either locally amplified, weakened, or slightly shifted in density space by localized processes (reflected in the high temporal correlation of *R*=0.88 between OW-In and OW-Out in Figure 3a and Figure 3c).

Therefore, the large O-West variance at σ_{SDmax} in CESM2 (Figure 1) is a downstream expression and modification of AMOC lower-limb anomaly pathway originating upstream. This is precisely why we describe the basin as a "*conduit*" – meaning a vital transit and modification corridor, rather than an isolated, primary generator of the anomaly itself.

2. Scientific Rationale for Retaining CESM2 in Figure 1.

CESM2 was retained in Figure 1 because it represents a conservative, "heavy-modification" upper-bound case among the models where localized O-West variance is pronounced. Our scientific logic is robust: if the connection to upstream anomalies and the density-space compensation mechanism remain clearly identifiable even in a model with such dominant local variance expression, our thesis holds true across the board. Replacing CESM2 with a model where O-West variance is inherently faint would constitute an artificial "cherry-picking" of a favorable example, which we intentionally avoided to maintain scientific integrity.

Regarding Point 2:

The reviewer suggests that our point-by-point correlation analyses in Figures 3 and 4 introduce a "methodological mismatch", arguing that our scientific focus would be better and more directly supported by simply displaying the variance of the streamfunction across density levels.

We respectfully but fundamentally disagree with this assessment, which heavily oversimplifies the dynamic complexity of the subpolar AMOC and overlooks the core scientific questions posed in our introduction. Plotting the variance structure across density levels, which we already present in Figure 1 and Supplementary Figures, only answers a baseline descriptive question: "where are the density-space anomalies largest?"

Crucially, a specific density class can exhibit huge variance while remaining completely weak correlation with the large-scale AMOC overturning. Relying solely on a variance metric, as the reviewer suggests, would falsely misinform the reader that these strong local signals are the primary drivers or components of the large-scale AMOC variability.

The fundamental objective of our manuscript is to look beyond mere variance magnitudes and explicitly test "which" specific density classes actively carry, transfer, or modulate signals that are related to the integrated OSNAP-Full and downstream MOC strength (Ψ_{max}).

Prior to our study, it was entirely unknown whether the dense water classes exhibiting the highest localized variance would also exhibit the strongest correlation with the AMOC. Our correlation analyses in Figures 3 and 4 yield a major, novel conclusion of this work: the density layers with the largest variability are *not* necessarily those with the strongest coherence to the large-scale MOC.

Figures 1 and 4 are deliberately complementary rather than contradictory: Figure 1 maps the magnitude of the signal, while Figures 3 and 4 diagnose its causal and coherent link to the broader AMOC system. As these critical physical and methodological distinctions are already thoroughly laid out and fully integrated across the existing results sections of our manuscript, no further modifications are required.

Reviewer #5 (Remarks to the Author):

After reading the full manuscript and the supporting material in detail I find that the manuscript's central conclusions are supported by the analyses and that the remaining disagreement reflects a misunderstanding of Figure 1 by the reviewer. My brief remarks below further explain how I came to this conclusion.

The introduction poses a very specific unresolved question raised by earlier published papers – how does transport variability in the density classes of the lower limb of the AMOC relate to the variability of the AMOC itself. The manuscript carries out a clear and careful analysis of couple climate models at different timescales – Low Frequency meaning multi-decadal, and High Frequency meaning Interannual. This is important because it has been argued that the mechanism relating transport in the deep waters to the strength of the AMOC may differ at longer and shorter timescales. Ultimately, they show the same signals in the HF timescales as the LF which is very interesting and possibly not what we might expect to see – but on both timescales the central conclusion of the paper is supported by the evidence shown by the analysis. They use OSNAP observations to test the conclusions from the model analysis and find that they are too support the findings.

I find that the authors take the reader through the steps of the analysis very well. The logic is straightforward, understandable and valid. The figures are clever – the reader needs to take the time to understand what they show by reading the text and studying the captions to understand the data and the colors, but on doing that they are startlingly clear in leading the reader to the same conclusion as the authors.

Turning to the remaining disagreement after a thorough reading of the paper, I conclude that the reviewer concern is centered on two minor misunderstandings by the reviewer, both of which are addressed in the rebuttal statements.

Firstly, the reviewer asserts that the full OSNAP MOC is the sum of OSNAP W and OSNAP E, which is incorrect. This concept was explained in the first OSNAP papers (Lozier et al 2017, Lozier et al 2019) and has been re-explained in every OSNAP paper since then.

The main concern is the second point however – the reviewer misinterpreted the high variability in the streamfunction at around 27.8 in OSNAP West as demonstrating the highest variability in the MOC is found in OSNAP W. This is not correct as is now explained in the version of manuscript that I am reading. It is a key point in the analysis that high variability in the overturning streamfunction in density space below the maximum of the streamfunction, ie in a class denser than the definition of the MOC, does not relate to the variability in the strength of the MOC itself. When one understands that distinction, the suitability of the

model as an illustration of the analysis is no longer a cause for objection. The authors have added to text to ensure other readers find it easier to see what the figure is showing, and the additional figure in Supplementary Material helps allay any concerns that this chosen model might not be representative of the multi-model mean.

In conclusion I find that manuscript's "Labrador Sea as a conduit" conclusion is consistent with Figure 1.

We are grateful to Reviewer #5 for their thorough, insightful, and constructive evaluation of our manuscript. We very much appreciate the reviewer's strong endorsement that our logic is "straightforward, understandable and valid," and that our central conclusions regarding the Labrador Sea as a passive conduit are robustly supported by both the multi-model analysis and the OSNAP observations.

In particular, we would like to express our respect for the reviewer’s sharp insights and scientific clarity. Reviewer #5 identified and clarified the persistent conceptual misunderstandings regarding the OSNAP MOC definitions and density-space streamfunction variability. Following the reviewer's comments, we have ensured that these key distinctions remain transparent in the text to maximize clarity for the broader community.

Reviewer #1
(Remarks to the Author)

This study examined the overturning circulation variability in the North Atlantic, focusing on connections between the OSNAP sections and the downstream AMOC. They found that (i) overturnings in the Irminger and Iceland basins, rather than the Labrador Sea, show great correlations with the AMOC. They also suggested that (ii) the Labrador sea overturning variability may be inherited from the upstream Irminger/Iceland basins. While I agree with their key point #(i), which is not surprising based on previous observational/modelling studies, I don't think their point #(ii) is correct (Or at least it is not supported by the current analysis; See major concern #3 below).

Major Concerns

[1] This study is partly motivated by the discussion of Yeager et al. (2021), but the authors appear to misunderstand the key messages in that paper, which by no means speculated a better correlation between OSNAP-West and AMOC than OSNAP-East. Yeager et al. (2021) suggested that deep convections in the Labrador Sea precede the multidecadai variability of the AMOC by roughly a decade (their Figure 7) -- this is achived by modifying the gyre circulation in the subpolar North Atlantic. It does not imply a more significant role of the Labrador Sea in driving the AMOC if this is quantified using correlation. If a similar analysis (Figure 7) were carried out in these coarse-resolution models, how does it compare with Yeager et al. (2021)?

[2] Based on previous observational/modelling studies (e.g., Lozier et al., 2019; Menary et al., 2020), it is not surpring that the highest correlation is between the AMOC and O-East (σ_{moc}), especially on multi-decadal timescales, given that most of the transformation occurs in the Iceland and Irminger Seas (Petit et al., 2020). The variability of overturning circulation streamfunction in the Labrador Sea, peaking at a higher density than σ_{moc}, primarily represents a redistribution of deep water transport between water masses denser than σ_{moc}. Why should we expect a direct correlation between O-West (σ_{SDmax}) and the AMOC? The key message of Yeager et al. (2021) is that deep convections in the Labrador Sea have merits for predictions of the AMOC, with respect to its internal variability on multi-decadal timescales. What do these in-phase correlations, discussed in this paper, tell us? What is responsible for the large inter-model spread? Can we trust all these models?

[3] The authors suggested that the variability in the Labrador Sea was advected from the upstream Irminger/Iceland basins. This is true for input/output volume transport across O-West, but how does this relate to the overturning criculation variability? The transports (OW-In, Ow-Out, and OE-Out) are part of the subpolar gyre circulation -- it would be surprising if they were not correlated with each other. The overturning circulation across O-West, by definition, is the residual between OW-In and OW-Out, and must be balanced by water mass transformation or volume tendency terms (e.g., expansion/contraction of LSW) in the Labrador Sea. If no transformation occurs and the water mass volume does not evolve in the Labrador Sea, OW-In will be exactly balanced by OW-Out. Thus, the overturning circulation variability in the Labrador Sea or the compensation does not "reflect the influence of upstream signals". At least, this is not supported by the current analysis (Lines 199-231).

Minor Comments

- [Lines 68-70]: Why are these 9 models selected? How do these 9 models reproduce the results of OSNAP regarding the mean overturning circulation?
- [Figure 1]: There appears to be some compensation between O-West and O-East around 27.7 kg m^{-3} (the variability of O-Full is much smaller than O-West and O-East). Any suggestions on what processes are causing this?

Review of revised version of “The Labrador Sea as a conduit of AMOC variability on multiple time scales” by Chen and Lozier

The authors have addressed all the concerns raised. There are, however, a few points where the manuscript would benefit from further clarification or discussion:

- The use of OE-Out, OW-In and OW-Out in the Discussion needs to be reviewed. As it stands, it is included in the discussion as if these quantities represent inflow and outflow contributions to the diapycnal overturning at OSNAP East, West & 50N. However, they are simply volume transport anomalies in a density range near sigma_SDmax. The description in the figure 3 caption (lines 236-237) states “Transport anomalies are computed by integrating over the two density bins just below $\sigma SDmax$.”. This range should be included in the text more explicitly rather than relying on Supplementary Information. It is not clear how this fits with lines 476-480, which describe the terms as “the total southward transport across O-East (OE-Out), the total northward transport into O-West (OW-In), and the total southward transport exiting O-West (OW-Out)”. By referencing equation (1) at the end of the paragraph it creates the impression that the terms are a cumulative integral from the maximum density to $\sigma SDmax$. Please review the text carefully to ensure that the reader is not misled.

- Can you be more precise as to what extent this is boundary current vs. interior phenomenon (alluded to on line 222 but not elaborated on thereafter). Do these isopycnal layers outcrop at the sea surface in the boundary current, if not why would we expect the layer thickness to change significantly along-stream (mainly isopycnal mixing in the Labrador Sea boundary current - interior region)?

- Are the transport anomalies present on outflow at OSNAP East (OE-Out) a reflection of surface buoyancy forcing in the Iceland and Irminger basins? Where do these layer thickness anomalies originate from? This is missing from the discussion currently.

- Is one of the implications of lines 381-384, that quantifying basin-scale stream functions in density compensated regions is partially flawed, and that focusing on the outflow of the Labrador Sea (DWBC) may be a more useful indicator of downstream AMOC variability? This fits with the text on lines 414-416, but perhaps your results suggest that outflow is a better choice than lower-limb variability?

- Eq 1 should use a dummy integration variable (σ') to be mathematically consistent:

$$\Psi(\sigma) = -\int_{x_w}^{x_e}\int_{\sigma_{max}}^{\sigma} v(x,\sigma')\, d\sigma'\, dx$$

- Lines 550-553: During this interpolation do you also rotate the velocity components to be normal to the section?

- Figure 11 (suppl.): y-axis labels are missing

I appreciate the authors' effort to address my concerns, but I remain unconvinced that the AMOC variability at σ_{SDmax} originates primarily from the Iceland and Irminger basins.

Consider the isopycnal transport across OSNAP-Full, T_σ (e.g., between $[\sigma_{\mathrm{SDmax}} - \delta\sigma]$ and σ_{SDmax}; positive southward)—this connects to lower-latitude AMOC variability (Fig. 1c). The AMOC variability at σ_{SDmax} in Figure 1d,f primarily reflects variations in T_σ, which is the sum over OSNAP-West and OSNAP-East:

$$T_\sigma = T_\sigma^W + T_\sigma^E,$$

where $T_\sigma^W = T_\sigma^{\mathrm{W-Out}} - T_\sigma^{\mathrm{W-In}}$ and $T_\sigma^E = T_\sigma^{\mathrm{E-Out}} - T_\sigma^{\mathrm{E-In}}$. These residuals are modulated by water mass formation, driven by surface forcing and interior mixing, and local volume changes between isopycnals within each basin.

The variance in T_σ decomposes as:

$$\mathrm{Var}(T_\sigma) = \mathrm{Var}(T_\sigma^W) + \mathrm{Var}(T_\sigma^E) + 2\mathrm{Cov}(T_\sigma^E, T_\sigma^W).$$

Figure 1d,f shows the AMOC variability near σ_{SDmax} is primarily associated with T_σ^W, with negligible contributions from T_σ^E and the covariance. This points to processes within the Labrador Sea, not the Iceland/Irminger basins, noting that it is the residual betwen inflow and outflow, not the outflow itself, that is related to the overturning.

The core issue: the authors appear to equate AMOC variability with variability in $T_\sigma^{\mathrm{W-Out}}$ rather than T_σ. This is incorrect for OSNAP-Full. Across a constant latitude it is also incorrect, because $T_\sigma^{\mathrm{W-Out}}$ exiting the Labrador Sea only partially accounts for the lower branch of the AMOC. This is easily testable: compare $T_\sigma^{\mathrm{W-Out}}$ variability to the net AMOC transport variability at the same latitude or across OSNAP-Full. They will differ substantially.

I am also concerned that different models were used for Figure 1 vs. Figure 3—CESM2 has a stronger Labrador Sea overturning than MPI-ESM1-2-HR and OSNAP observations. However, this does not change the central point even if MPI-HR is used to reproduce Figure 1: the Labrador Sea makes a sizable contribution to AMOC variability in all models examined except for ACCESS and CanESM5 (Supplementary Figure 2).

In summary, the results do not support the conclusion, at least not the claim that the Labrador Sea acts as a conduit for AMOC variability. I would be happy to see rebuttals from the authors, but if they agree with the points above, the conclusion should be overhauled accordingly.

This is the fifth round of review. While the authors have provided additional clarifications, a fundamental misalignment remains between the primary figures and the main conclusion. Given that multiple rounds of review have not resolved this structural contradiction, I recommend that the manuscript be rejected as further revisions are no longer productive.

The manuscript retains key logical and structural contradictions that need to be addressed on a purely physical basis:

1. Misalignment of figures and conclusion

The authors are entirely correct in their response that the MOC strength does not simply add up across sections, as the maximum overturning occurs at different densities (σ_{moc}) for different regions. However, my previous comments were not referring to the peak MOC strength (Ψ_{max}). I was explicitly referring to the continuous, density-space overturning streamfunction, $\Psi(\sigma)$.

By fundamental definition, at any given density layer σ, the full array streamfunction is the exact sum of its components:

$$\Psi_{\text{Full}}(\sigma) = \Psi_{\text{West}}(\sigma) + \Psi_{\text{East}}(\sigma).$$

When evaluating the streamfunction profile rather than the peak strength, the add-up works exactly. In CESM2 (Figure 1), a variance decomposition demonstrates that OSNAP-West dominates the AMOC streamfunction variance at σ_{SDmax}. This mathematically requires that the Labrador Sea is the primary driver of the overturning variability at this dense layer in this model, which directly contradicts the study's main conclusion that the basin acts as a passive conduit.

In Figure 1, the authors acknowledge that OSNAP-West dominates the streamfunction variance at the dense lower-branch layer (σ_{SDmax}). This means the Labrador Sea is the primary generator of lower-branch streamfunction variability in this model, flatly contradicting the paper's core thesis that the basin is merely a passive "conduit."

Nevertheless, the authors insisted on keeping this figure–this is not acceptable.

2. Methodological Mismatch: Lower-Branch Transport vs. Maximum MOC Strength

There is a disconnect between the stated focus of the paper and the metrics used in the analysis. The title and abstract highlight the role of OSNAP-East in driving the *lower-branch transport* of the AMOC. However, in Figures 3 and 4, the analysis relies on correlations between the overall maximum AMOC strength (Ψ_{max}) and the subpolar overturning streamfunction at each individual density level.

If the goal is to evaluate lower-branch transport anomalies, these point-by-point correlations against the maximum MOC strength are unnecessary and introduce a methodological mismatch. The conclusions regarding the subpolar ocean's role would be more directly and transparently supported by simply plotting the variance of the overturning streamfunction across

density levels for the ensemble.
